# Biological impact of mutually exclusive exon switching

**DOI:** 10.1371/journal.pcbi.1008708

**Published:** 2021-03-02

**Authors:** Su Datt Lam, M. Madan Babu, Jonathan Lees, Christine A. Orengo

**Affiliations:** 1 Institute of Structural and Molecular Biology, University College London, Darwin Building, Gower Street, London, United Kingdom; 2 Department of Applied Physics, Faculty of Science and Technology, Universiti Kebangsaan Malaysia, Bangi, Malaysia; 3 MRC Laboratory of Molecular Biology, Francis Crick Avenue, Cambridge Biomedical Campus, Cambridge, United Kingdom; 4 Department of Structural Biology and Center for Data Driven Discovery, St Jude Children’s Research Hospital, Memphis, Tennessee, United States of America; 5 Faculty of Health and Life Sciences, Oxford Brookes University, Oxford, United Kingdom; San Raffaele Hospital: IRCCS Ospedale San Raffaele, ITALY

## Abstract

Alternative splicing can expand the diversity of proteomes. Homologous mutually exclusive exons (MXEs) originate from the same ancestral exon and result in polypeptides with similar structural properties but altered sequence. Why would some genes switch homologous exons and what are their biological impact? Here, we analyse the extent of sequence, structural and functional variability in MXEs and report the first large scale, structure-based analysis of the biological impact of MXE events from different genomes. MXE-specific residues tend to map to single domains, are highly enriched in surface exposed residues and cluster at or near protein functional sites. Thus, MXE events are likely to maintain the protein fold, but alter specificity and selectivity of protein function. This comprehensive resource of MXE events and their annotations is available at: http://gene3d.biochem.ucl.ac.uk/mxemod/. These findings highlight how small, but significant changes at critical positions on a protein surface are exploited in evolution to alter function.

## Author summary

A single gene can produce more than one protein through processes such as Alternative Splicing. Mutually exclusive splicing is one type of splicing by which cells produce isoforms that are often very similar in their three-dimensional shape. Different transcript isoforms are usually produced in different cell tissues and cell environments. The structural impacts of this splicing are still not well-studied. In this study, we compare proteins generated from mutually exclusive exon switching from 5 organisms. Most of these proteins do not have their three-dimensional shape solved by experimental methods. We therefore calculate the most probable three-dimensional structural models. We found small regions that are different between the proteins and occur near important residue sites that determine the functions of the proteins. We speculate mutually exclusive splicing may be involved in tuning the protein function. We have built a webserver to present information on these exon switching events: http://gene3d.biochem.ucl.ac.uk/mxemod/.

## Introduction

Alternative splicing (AS) refers to the assembly and rearrangement of different exons of a gene during pre-mRNA splicing such that different mRNAs and thus proteins are produced from the same gene. Alternative splicing is common in humans and other animals producing different transcripts in different developmental stages, tissues or disease states [1,2]. Alternative splicing events have been linked to various diseases and cancers [3–6] and may lead to the tissue-specific rewiring of protein-protein networks [7–9]. A biologically meaningful role for many AS events has been demonstrated [10–12], but the extent to which AS extends the functional repertoire is a subject of considerable active debate in the literature [13,14].

Amongst the most important exon-switching events are those that generate homologous mutually exclusive exons (MXEs), such that only one out of the two exons is retained while the other one is always spliced out. This type of exon switching is less likely to be disruptive to highly organised globular protein structure [14,15] compared to AS events such as cassette exon removal. Consistent with this notion, homologous MXEs have been found to be highly enriched in proteomics experiments [16] and to be more conserved between species [17] compared to alternative transcripts generated by AS in general. MXEs have been shown to be enriched with muscle and membrane functions (such as transporter, signal transduction) [18,19].

**Fig 1:**
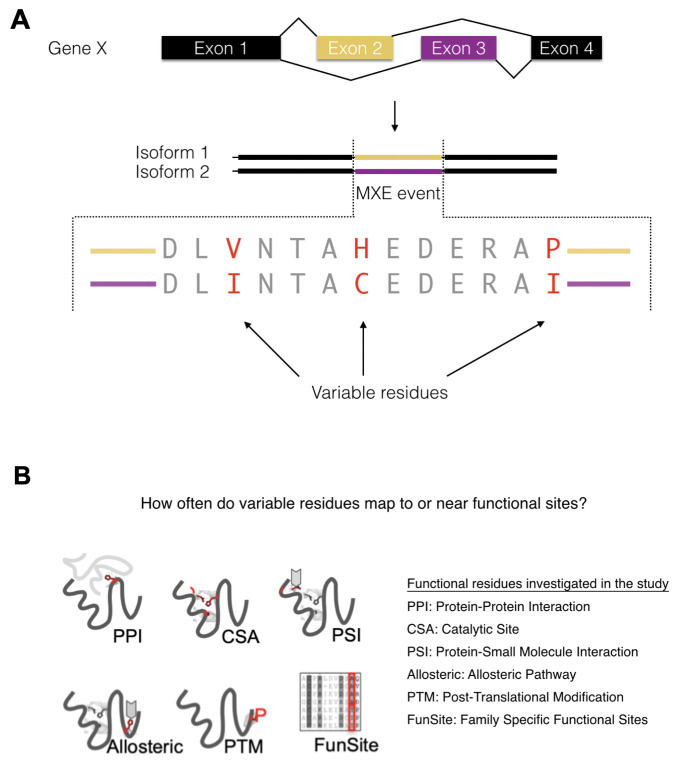
Identification of variable residues and possible functional effects of variable residues. (A) Identifying the variable residues for an MXE event (MXE-specific residues). The same colour code is used throughout the paper. The MXE region from the pair of proteins generated from the splicing are shown in purple or yellow. Variable residues from the MXE event are shown in red. (B) Possible functional effects of variable residue switching assessed in this paper include altering PPI (Protein-Protein interactions), CSA (catalytic residues from the Catalytic Site Atlas[21]), PSI (Protein-Small molecule Interactions), Allosteric, PTM (Post-Translational Modification) and FunSites (predicted functional sites from functional families (FunFams)).

It is possible to align a pair of homologous MXE events and identify conserved and variable amino acid residues, the latter of which could be considered responsible for any functional shifts of the MXE (Fig 1A). That is, changes in amino acids could contribute to changes in specific functions and there are several examples of this in the literature (Fig 1B). For instance, specific examples have been found for which MXE events modulate the binding of the protein to a substrate (e.g. protein, ion) [14,17] and have been shown to alter the voltage dependencies of ion channels [20].

Given that MXEs are likely to affect protein function, how often and what types of functions are altered? Here, we analyse multiple genomes and use large scale protein structure modelling data to assess the functional impact of these processes and their likely biological role. We describe the mechanisms by which MXE events trigger dynamic switching of protein surface patches associated with key solvent exposed functions (i.e. binding to other biomolecules such as proteins, small molecules, etc.). We subsequently illustrate how aberrations in the same functional switching regions altered by MXE events may be mutated leading to deregulation in cancer. Unlike MXE events, many AS events such as cassette exon removal in globular regions, are likely to lead to significant disruption of the protein structure. However, for a subset of cassette exon splicing events where there is less likely to be structural disruption, and microexons in general we do not identify the same functional enrichments as for MXE events. Finally, we present a comprehensive online resource of the structural and functional annotations of MXE events to allow users to search for MXEs of interest to investigate the structural/functional consequences of these events.

## Results

### Comprehensive protein structure modelling of MXE events from multiple species

**Fig 2:**
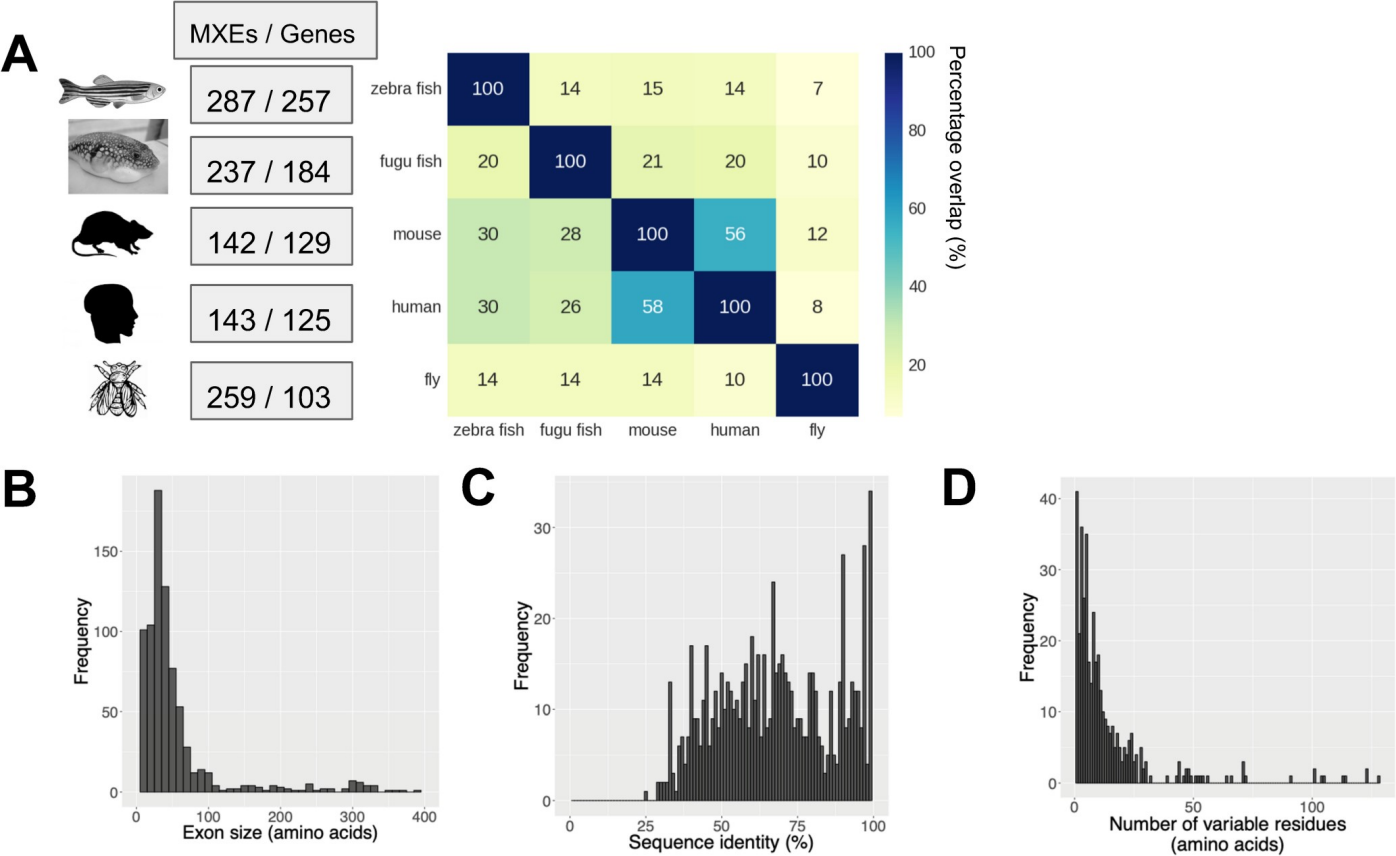
General statistics of our MXE dataset. (A) Table showing the number of MXE events and MXE genes in each organism and a matrix with the percentage overlap of MXE gene orthologs between species. The percentage overlap refers to percentages of overlap between gene orthologs between species (e.g. 58% of human MXE genes have an ortholog in mouse). (B) Distributions of MXE exon sizes. MXE sizes were calculated by calculating the length of the MXE. The length of the longer isoforms were plotted. (C) Distribution of sequence identity between MXE pairs identifies one main distribution with its main peak between 50% and 70% sequence revealing likely conserved structure and function. A second much smaller peak around 90% indicates a subset of MXE events that are more recently evolved. Sequence identities were calculated by BLASTing two isoforms against each other. (D) Distribution of number of variable residues between MXE pairs demonstrates that the majority of MXEs have only a limited number of variable residues with almost all events <10 variable residues. Variable residues are amino acid residues that are variable in the alignment between MXE pairs.

Through a comprehensive genome-scale analysis pipeline, we first identified homologous MXEs in 5 high-quality Metazoan genomes (human, fly, mouse, fugu fish and zebrafish) (see **Materials and Methods**). In this way we could compare results across genomes to examine any potential similarities and variations in the different species. We find that in our dataset, each organism has over one hundred MXE containing genes (Fig 2A, see S1 Table for list of MXE genes from each species). The MXE protein sequence pairs in our dataset typically have high sequence identity with a mean of 67% (Fig 2C) and a median length of 36 amino acids (Fig 2B). Apart from the major peak there is a second smaller peak at higher sequence similarity, which could arise from a number of factors including biases in protein evolutionary rate [22]. Functional differences in protein sequences containing the MXE will arise from the variable residues (i.e. amino acid residues that are variable in the alignment between MXE exons, Fig 1). There are usually less than 10 variable residues (median = 6 residues) (Fig 2D).

An analysis of the evolutionary conservation of MXE events revealed that 58% of human MXE genes have an ortholog in mouse that is also an MXE gene, but for other organisms the gene overlaps were considerably lower (Fig 2A). This relatively low overlap of MXE genes in our dataset, does not mean the MXEs are absent from these other organisms, since more targeted studies point to general conservation of MXEs, but does illustrate that our multi-species approach considerably extends the scope of our analysis by giving many additional MXE events, to better reveal the general principles of MXEs.

CATH superfamilies are groups of homologous protein domains (i.e. having significant structural and/or sequence similarity) [23]. Each CATH superfamily can be further subdivided into functional families (called FunFams) providing more structurally and functionally coherent sets of protein domain homologues. FunFams have been used for predicting protein functions and for analysing the possible functional impacts of disease variations (i.e. residue mutations) in proteins [23,24]. We analysed the structural features of our MXE events i.e. their location in the protein structure and their proximity to known and predicted functional sites, using the FunMod pipeline (derived from CATH FunFams, see **Materials and Methods** and cited references [25,26]) and we supplemented this using direct structural mapping guided by sequence alignments (see **Materials and Methods**). The results showed that whilst only a very small number of MXE events can be mapped to known structures, we can massively expand our structural coverage using our structural modelling strategy, from 14 to 691 MXE events.

**Fig 3:**
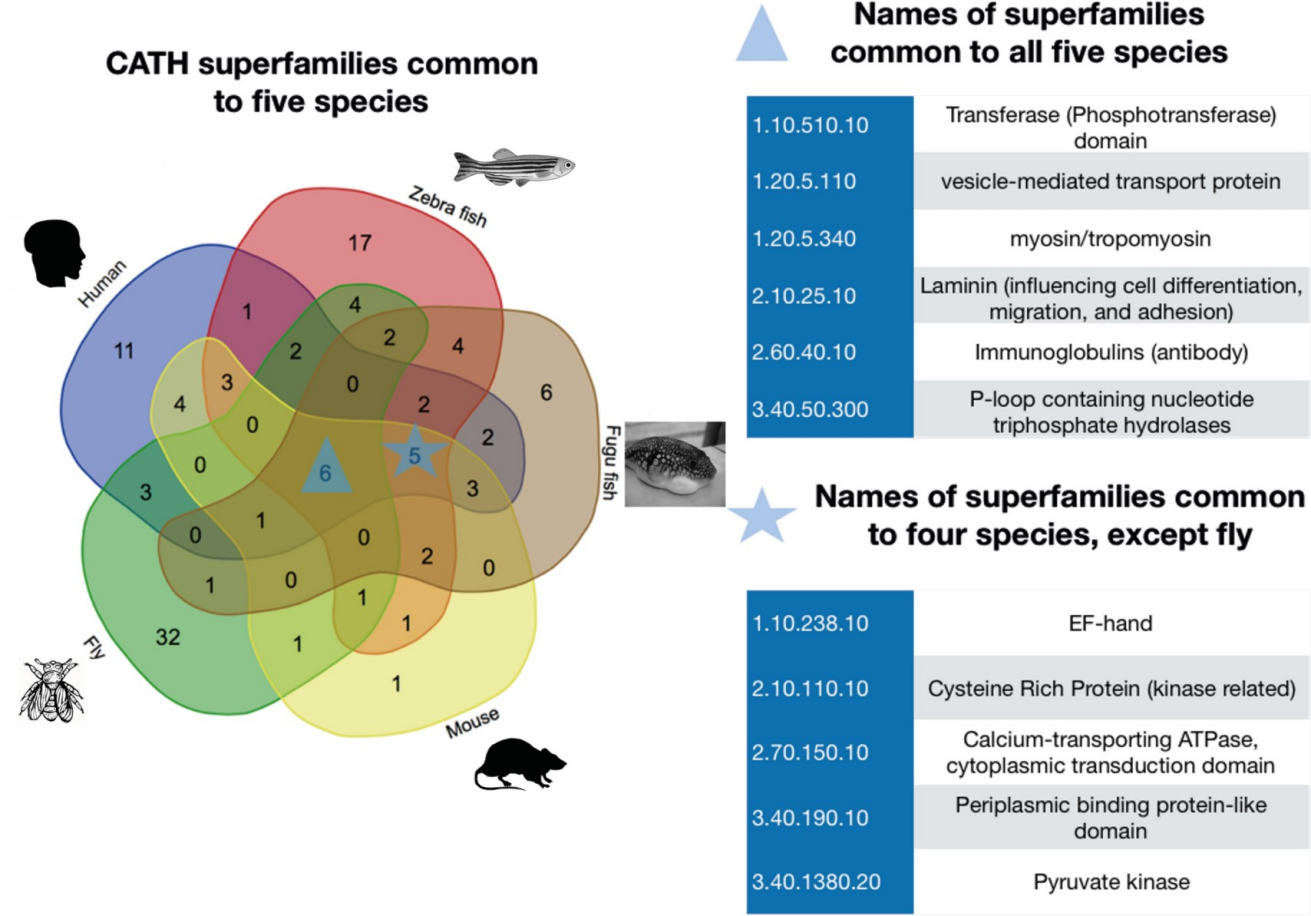
Number and names of CATH superfamilies common to multiple species analysed in this study. MXEs from different species were mapped to CATH domain superfamilies. CATH superfamilies are groups of protein domains with clear evidence of homology. The CATH superfamily code is denoted by four numbers corresponding to each level in the CATH classification (i.e. 3.20.20.120). At the top of the hierarchy is the class level where structural domains are classified based on their secondary structure content. The second level of the hierarchy is the architecture level given by the global arrangement of secondary structures in 3D space. This is followed by the topology level where domains with similar folds (which takes into account the 3D arrangement, orientations and connections between the secondary structures) are grouped together. The fourth level is the homologous superfamily level where domains are deemed homologous. A website tool (**http://bioinformatics.psb.ugent.be/webtools/Venn/**) was used to draw this Venn diagram.

The MXE regions structurally mapped to a relatively small number (115 out of a possible 2700) of CATH superfamilies (Fig 3). Preliminary analysis of the CATH superfamily functions revealed that the MXE domain families are enriched in important Metazoan functions such as membrane proteins involved in cell-cell adhesion and signal transduction (see S1 Fig). Functional analysis by the PANTHER pipeline [27] showed additional functional enrichments from MXE genes, in membrane proteins (e.g. ion channels, synaptic vesicles and receptors, FDR level <0.01; See S2–S6 Figs for details). Our analyses show similar enrichments as other previous MXE studies [18,19].

Why have some proteins, such as ion channels, synaptic vesicles and receptors evolved by expanding their functional repertoire through MXE events? There could be some high level of association between these MXE containing genes that has not yet been considered. For example, one might suspect that MXE functional expansion is somehow advantageous for genes expressed in certain tissues since rewiring of tissue specific networks is thought to be a function of other types of protein isoform switching [7]. TopAnat provides a method to detect anatomical terms that are enriched in a list of genes (in terms of their expression in different anatomical regions of human tissues) [28,29]. Using the TopAnat website, we found a significant enrichment for human MXE genes to be expressed in various brain anatomies, with the highest significance for the anterior prefrontal cortex (Brodmann (1909) area 10) (FDR = 2e^-6^, Fold enrichment = 2.05). Brain and neural tissue enrichments can also be found for the other classes of splicing (cassette exon and microexon, discussed in S2 and S3 Texts). Other studies have shown enrichment of AS events in neuronal tissue [30]. Here we are simply capturing the tissue gene expression enrichment (rather than the occurrence of splicing in these tissues). Our analysis, therefore, implies that MXE genes tend to have functions associated with neuronal tissues.

Finally, using a set of FDA druggable proteins from the Human Protein Atlas (HPA) [31], we found significant enrichment of druggable proteins in our human MXE genes (fishers-test = p-value 7.8e^-5^, odds-ratio = 3.9). In total 13 of the 672 FDA approved genes available from HPA, could be mapped to one of the 125 human MXE genes (see S2 Table and MXE website for list of druggable targets). This indicates that MXE genes are in general likely to be interesting targets for disease therapies. An example of a known drug targeting an MXE gene is a voltage gated Calcium channel CACNA1D which is targeted by a number of drugs for hypertension such as Amlodipine (DrugBank:DB00381). We saw a similar enrichment for druggable genes using another dataset of MXE events compiled by Hatje and co-workers and described in S1 Text (p-value < 1.2e^-6^, odds-ratio = 2.8). We also analysed single exon loss events (see **Materials and Methods**) and saw a significant enrichment (p-value = 9.7e^-6^) but with a substantially lower odds-ratio of 1.6, whilst microexons showed no enrichment.

### Large-scale structural analysis using 3D structures and models shows that MXE events predominantly modulate surface residues and protein binding functions

**Fig 4:**
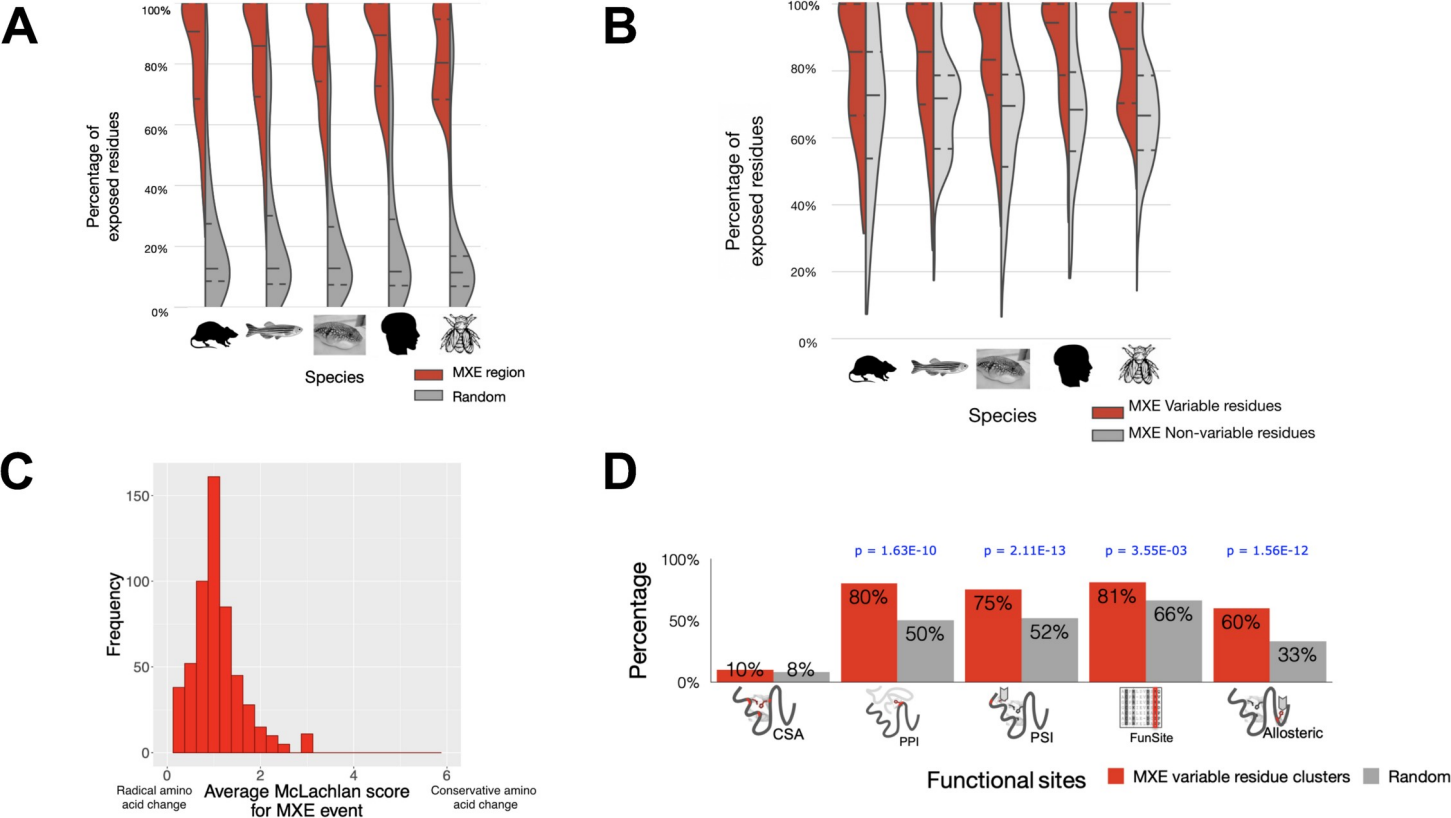
Structural and functional analysis of the MXE splicing dataset. (A) The surface exposure of the MXE events compared to random expectation. The first quartile, median, and the third quartile of the population were indicated with lines. NACCESS was used to calculate the relative accessible surface (rASA) of amino acids. Amino acid residues were considered to be exposed if the rASA value was above 10%. (B) The surface exposure of the MXE variable residues compared to the MXE non-variable residues. We compared the solvent exposure of the variable residues versus the solvent exposure of the MXE conserved residues for each MXE event. NACCESS was used to calculate the relative accessible surface (rASA) of amino acids. Amino acid residues were considered to be exposed if the rASA value was above 10%. (C) Distribution of McLachlan scores for the variable residues. For each MXE, we summed up all the McLachlan similarity scores for a set of variable residues and divided by the total number of mapped variable residues to normalise the score. (D) Proximity of MXE variable residue clusters to amino acids assigned to different functional classes (indicated by Icons) CSA = catalytic residues from the Catalytic Site Atlas, PPI = protein interaction sites and PSI = protein-small molecule interactions). A breakdown of this Fig by individual species is available in S10 Fig. We used the Z-score test to compute the statistical significance. Where appropriate the level of statistical significance is shown above each type of functional site.

The function of a protein domain is largely dictated by the shape and nature of the residues on the protein surface. Enrichment of MXE events on surface residues would indicate absence of an effect on the protein fold but an effect on function such as altered protein interactions, protein-small molecule binding, etc. Our structural mapping approach (see **Materials and Methods**) provided structural information to assess this (S7 Fig) for 691 MXE events. We observed that the percentage of exposed residues for an MXE region is significantly higher than what one would expect by chance (Fig 4A) (p-value = 1.9e^-144^, Wilcoxon signed-rank test). The level of the difference was striking and indicates that MXE events are strongly biased to altering residues with surface exposure. This picture is consistent across multiple organisms and indicates this is a prominent and consistent feature of MXE splicing.

Are the variable residues involved in MXE events more exposed to the solvent than the MXE region itself? A more detailed analysis, involving buried surface area calculations for all the MXEs revealed that variable (i.e. MXE-specific) residues are much more significantly exposed than non-variable MXE residues (Fig 4B; p-value = 2.3e^-37^, Wilcoxon signed-rank test). These observations further demonstrate that the amino acid changes in MXE events consistently alter surface exposed residues on the protein, much more than we would expect by chance. Together these findings collectively suggest a role for these surface variable regions in modulating protein functional sites.

How drastic are the amino acid changes in the variable sites and do such changes happen at or near functionally relevant sites on the structure? Alterations in the variable residues for an MXE event can be classed as conservative or radical using established residue substitution scoring matrices, with the latter more likely to have functional consequences. We compared the physicochemical properties of the equivalent MXE variable residues using the McLachlan physicochemical matrix [32] which captures chemical similarity between residues. Typically, McLachlan scores < 2 for an amino acid change are used to indicate a significant change in physicochemical properties [32]. For all the organism datasets, more than 90% of the MXE variable residues have a McLachlan score ≤2 (Fig 4C). These results indicate that in general the MXE events produce significant changes in physicochemical properties of the MXE regions. We also compared the physicochemical changes observed for the MXE variable residues using a random model which randomly samples residues within the same threshold distance of 4Å from a particular class of functional residue (catalytic, ligand binding, PPI). We find the tendency for chemical change in the MXE variable residues to be significantly different to random (all with p-values ≤ 6.35e^-125^, See S8 Fig). Such considerable changes suggest that MXE events are likely to cause functional shifts between protein isoforms, particularly if they lie on or close to functional sites.

Do the MXE variable residues cluster together on the protein structure (or are they dispersed) and do they tend to affect functionally relevant sites? A detailed analysis of the positioning of the MXE variable residues revealed that they tend to form a single cluster in space in over 70% of the MXE events. For those MXE events that formed multiple clusters, they usually formed 2–3 clusters (S9 Fig). Next, we determined whether these clustered variable residues map to known functional sites or lie close to them. We first identified well-established functional sites such as catalytic residues, positions that are known to mediate protein-protein and protein-small molecule interactions (PSI) based on crystal structures of available homologs within the functional family to which the MXE event has been mapped (Fig 1B). We also identified positions that are likely to confer functional selectivity using the FunSites approach (See **Materials and Methods**). Briefly, we mapped the MXE region onto CATH functional families (FunFams). Positions that are conserved within and unique to a particular FunFam but not conserved across the superfamily are likely to be specificity-determining positions and responsible for a specific function that is distinct from other superfamily members. Finally, we also identified putative allosteric sites using the A-SITE approach (see **Materials and Methods**).

Having annotated the residue positions, for every pair of splice isoform compared, we determined if the centre of mass of the cluster of MXE variable residues was close to any of the above-defined functional sites (within 6Å). We calculated the minimum distance between the atoms of the residues involved. A comparison with a random model (**Materials and Methods**) shows that there is a significant tendency for clusters of MXE variable residues to lie close to protein-protein interaction, protein-small molecule, specificity determining positions and allosteric sites for all species (Fig 4D) (all with p-values ≤ 3.55e^-03^, please refer to Fig 4D for details of these values). The signal was strongest for protein-small molecule interaction (PSI) and protein-protein interaction (PPI) functional sites. We also performed the analysis without clustering the variable residues and obtained similar results (see S11 Fig for details).

Through our structural modelling pipeline, integrated with functional site datasets, a clear picture emerges for a consistent role of MXE events modulating functional regions in domains. In line with previous work, our modelling suggests that the sequence variations in MXEs are typically small and although chemically quite extreme (as judged by the McLachlan index), they are usually surface exposed and therefore unlikely to affect the ability to produce stable structures but more likely to alter surface functions.

To confirm our observations, we performed a second independent analysis using the more comprehensive validated human MXE-splicing events from the Kassiopeia resource which we refer to as the Hatje dataset (N.B. this dataset was filtered to only include homologous exons). We found similar properties with the Hatje dataset further supporting the generality of our observations (See S1 Text and S19–S24 Figs). We also studied the structural and functional effects of MXEs with cassette exon splicing. We identified single exon loss events (see **Materials and Methods**) and found no enrichment of cassette exon near functional sites (i.e. PPIs, PSIs etc, See S2 Text and S25–S33 Figs). This along with enrichment in proteomics highlights an important and distinct functional role for MXEs. A sub-class of cassette exons are microexons which are small, surface exposed and therefore less likely to damage globular protein structure than cassette exons in general. We also found that they were surface exposed but did not find they were enriched in any of the functional locations we identified for MXEs (S3 Text and S34–S40 Figs).

### Variable regions in MXE events lie at or close to cancer mutation sites

How often do variable regions lie at or close to cancer mutation sites? Recent studies have suggested an association between human MXE events and inherited pathogenic mutations [18]. Furthermore, many studies have found that cancer mutation sites tend to lie in the vicinity of functional residues (i.e. protein and small molecule binding sites) [33,34]. MutFams are CATH functional families found to be significantly enriched in cancer mutations (see **Materials and Methods**). We therefore investigated whether MutFam cancer mutation residues coincide with both MXE variable residues and functional residues. We mapped putative cancer driver genes from MutFams to the 143 human MXE events. We found carcinoma (e.g. adenocariconoma, squamous cell carcinoma) and melanoma to be the common cancers that have mutations coinciding with MXE events (by analysing cancer annotations in the MutFam). Functional analysis by the PANTHER pipeline [27] on cancer-related MXE showed additional functional enrichments, in signalling process (e.g. oxytocin, adipocytokine and prolactin) and regulatory pathway (e.g. insulin secretion, transcription) (FDR level <0.01; See S12 Fig for details).

We could annotate 43 of these events with structures and we then determined if the variable residues/clusters in these MXE events were close to MutFam mutation residues. We found that 40 of the 43 MXE events had variable residues that were significantly closer (in terms of distance) to cancer mutations than expected by random (p-value <0.0001, see **Materials and Methods**). We obtained the same results for both per-residue and per-cluster analyses. We checked if these MXE events were close to functional residues and found 35 out of 40 of the events were close to functional residues. Similarly, there is a significant tendency for cancer mutation and variable residues to lie close to protein-protein interaction, protein-small molecule, specificity determining positions and allosteric sites. Hence, MutFam cancer mutations target a similar set of functional sites as are altered by MXE events, suggesting some advantage to cancer in modifying the same set of specific functional residues on proteins that are dynamically regulated by MXE events. We found similar properties with the Hatje dataset further supporting the generality of our observations (See S1 Text for more information).

### Molecular principles of the impact of MXE events

Results from the global analyses of MXE events using the approaches described above have suggested general principles and mechanisms by which MXE events influence protein function and how cancer mutations tend to affect MXE variable positions or fall near them, thereby influencing disease states. In this section, we present four examples that illustrate the above described findings (Further details of these examples are available in the S4 Text).

#### Protein interaction

**Fig 5:**
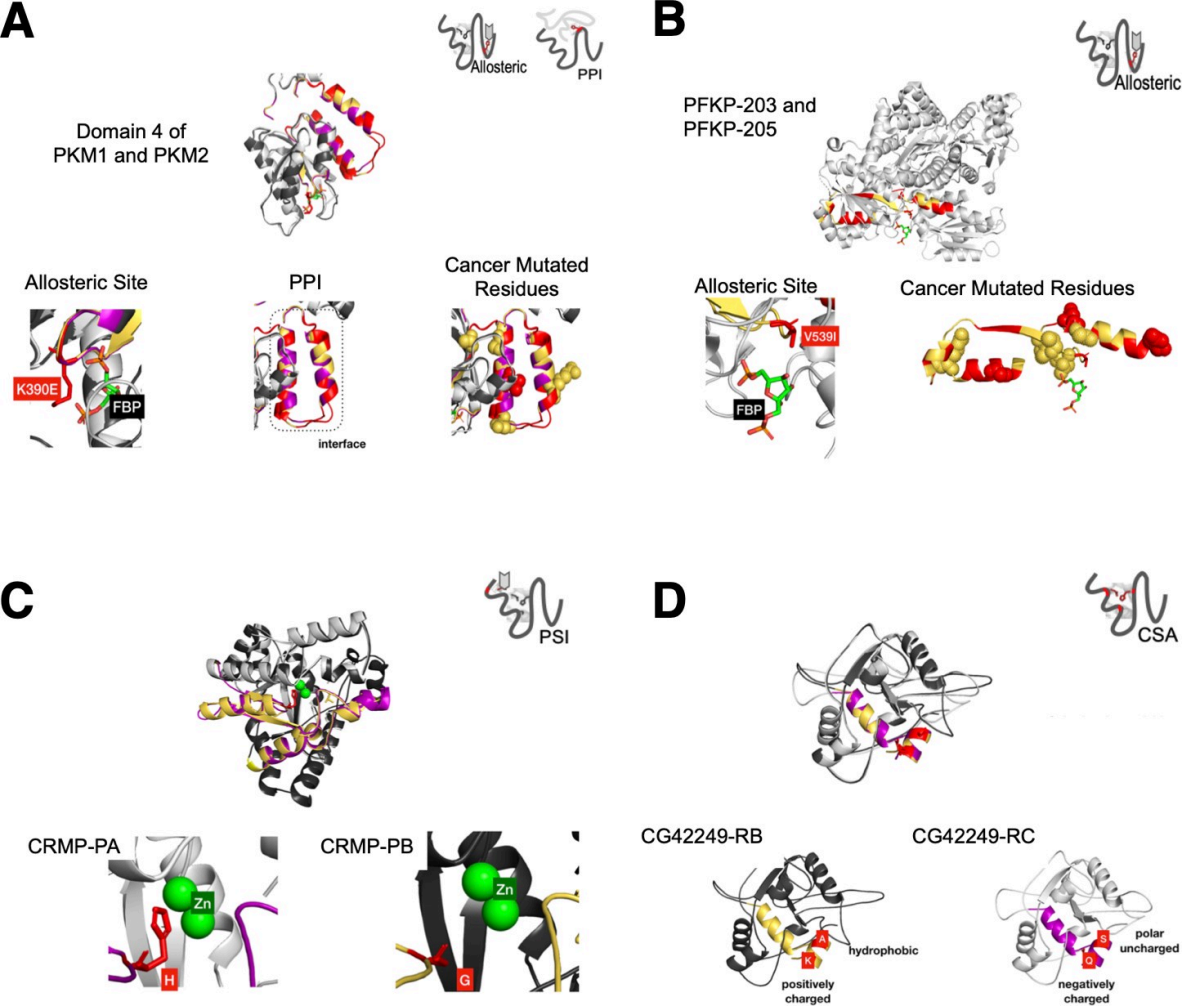
Examples of MXE events affecting amongst other things. (A) Protein-protein interaction of the PKM gene. Cancer mutated residues are shown in spacefill. (B) Allosteric regulation of the PFKP gene. Cancer mutated residues are shown in spacefill. (C) Small molecule interaction (PSI) sites in the CRMP gene. We are only showing variable binding zinc/catalytic residues. Zinc molecules are shown green spacefill. (D) Catalytic site residues of gene CG42249 from drosophila. We are only showing variable binding zinc/catalytic residues.

A total of 260 MXE events are associated with PPI functions. For example, the PKM gene is a pyruvate kinase enzyme with an essential role in metabolism and is important in the growth of cancer cells [35]. Although other isoforms are also present, the two main PKM isoforms generated by MXE splicing (known as PKM1 and PKM2 isoforms) and the MXE event alters variable residues of its fourth domain (S13 Fig), mostly on the protein surface (S13 Fig), associated with allosteric (by FBP small molecule interaction) and protein interaction (tetramerisation) functions (Fig 5A). PKM2 dimers and tetramers possess low and high levels of enzyme activity, respectively, and this functionality (oligomerisation state switching) is important for proliferating cells (when PKM is in its inactive dimeric state, preceding pathway intermediates build up and are driven down alternative pathways to generate biomolecules important for growth). Further details on this are given in S4 Text. Our analysis (see **Materials and Methods**, S14 Fig and S4 Text) show that the effect of the MXE variable residues is to reduce the complementarity of the tetramerisation interaction surface in PKM2 compared to PKM1, thus favouring PKM2 to be in the inactive dimeric state (this prediction was based on the Adaptive Poisson-Boltzmann Solver [36] to calculate surface potentials of MXE regions S14 Fig and S4 Text for values). We also used mCSM-PPI [37] to assess the effect of the MXE variable residue changes on protein-protein affinity and found most of the residues to be destabilising (See S15 and S16 Figs).

At the same time this allows PKM2 to be dynamically switched to the tetrameric active state depending on a cell’s requirements, which could allow cancer cells to more rapidly respond to changes in its environment. The dynamic switching to the active tetramer is provided by an allosteric effector, FBP, the binding of which is also provided by the MXE variable residues (discussed below).

#### Allostery

A total of 391 MXE events were associated with allosteric functions in our analysis. Analysis of PKM MXE structures (along with mutation data [38–40]) identified a key variable residue (Lysine 433) in PKM2 which modulates FBP binding ability (FBP does not bind PKM1). Previous studies have demonstrated that allosteric binding of FBP in PKM2 induces movement/rotations of the helices in the interface and the FBP activating loop that brings the tetramer binding residues into correct alignment for forming the tetramer [41,42]. Therefore, the ability to increase interface complementarity by binding FBP in PKM2 (switching from an inactive dimer to active tetramer) is encoded by the MXE variable residues (Fig 5A).

Interestingly, we find another tightly regulated glycolytic enzyme, that is also allosterically regulated, called Phospho Fructo Kinase-platelet (PFKP) (Fig 5B) and again in which the MXE event likely alters the allosteric regulation (See S17 Fig and S4 Text for detailed analysis).

Furthermore, the MXE regions of PKM1/PKM2 and of PFKP were found to overlap with pan-cancer mutations in structural regions close to functional sites (Fig 5A and 5B), reinforcing the idea that mutations in these regions impact on the function of the proteins and that cancer mutations tend to have similar effects on function as the effects mediated by MXE variable positions.

#### Small molecule ligand binding

A total of 235 MXE events were associated with protein-small molecule interaction functions. An example of this is the Collapsin Response Mediator Protein CRMP gene (FBgn0023023), a dihydropyrimidinase enzyme (EC 3.5.2.2) that has been implicated in biological processes including Notch signalling [43]. An MXE variable residue having zinc-binding function (residue 192), switches from a histidine in isoform CRMP-PA to a glycine in isoform CRMP-PB disrupting the binding of zinc (Fig 5C). Since zinc molecules act as cofactors in enzymes this is likely to reduce enzyme activity for the zinc free isoform. Interestingly, other variable residues in the CRMP MXE produce dramatic changes in the surface charge distribution (S18 Fig). Given the extensive inferred protein interactions for this protein from the IBIS resource (see **Materials and Methods**), some of which overlap the MXE variable regions, it suggests that MXE switching could also switch CRMP protein interactions.

#### Catalytic sites

We identify 9 MXE events that affect catalytic sites (CSA residues) in our dataset. For example, CG42249 a poorly functionally characterised fly gene is likely to be a nucleotidase (based on domain assignments and M-CSA conserved catalytic residues (PDB:1USH)). We identify switches in key catalytic residues between its MXE isoforms with major differences in physicochemical properties (Fig 5D). Such changes in physicochemical properties could produce enzymes with very different or even opposing functions and shows the utility of pipeline for identifying interesting candidates for further experimental studies.

### PTMs

#### Phosphorylation

Integrins are an important class of transmembrane proteins in animals mediating cell interactions and signalling in many processes (e.g. embryonic development). We identified an MXE in integrin-B1, where one isoform (ENSMUSP00000119699) has MXE variable residues substituting two residues (Thr788-Thr789 in ENSMUSP00000087457) from Thr to Asn, abolishing a known phosphorylation site. Hence even though the kinase may be expressed and active, only one isoform is capable of recruiting the kinase and so switching isoforms can produce changes in downstream signalling.

#### Glycosylation

KLRC2 is a transmembrane protein important for regulating Natural Killer (NK) cell-mediated immunity and has an MXE event that switches exon 3, changing 2 amino acids in the extracellular region of the protein. The substitution of Serine 102 to Phenylalanine by this MXE event would remove the glycosylation of amino acid N100 (identified from a ‘manual assertion’ in UniProt [44] and the N-glycosylation consensus motif). Searching for this MXE in KCLR2 primate orthologs, reveals that the MXE is absent from closely related species such as Orangutan, Bolivian squirrel monkey and Macaques suggesting this a highly dynamic MXE which could help tune the immune response in specific lineages by altering PTM states.

### MXE-MOD website

**Fig 6:**
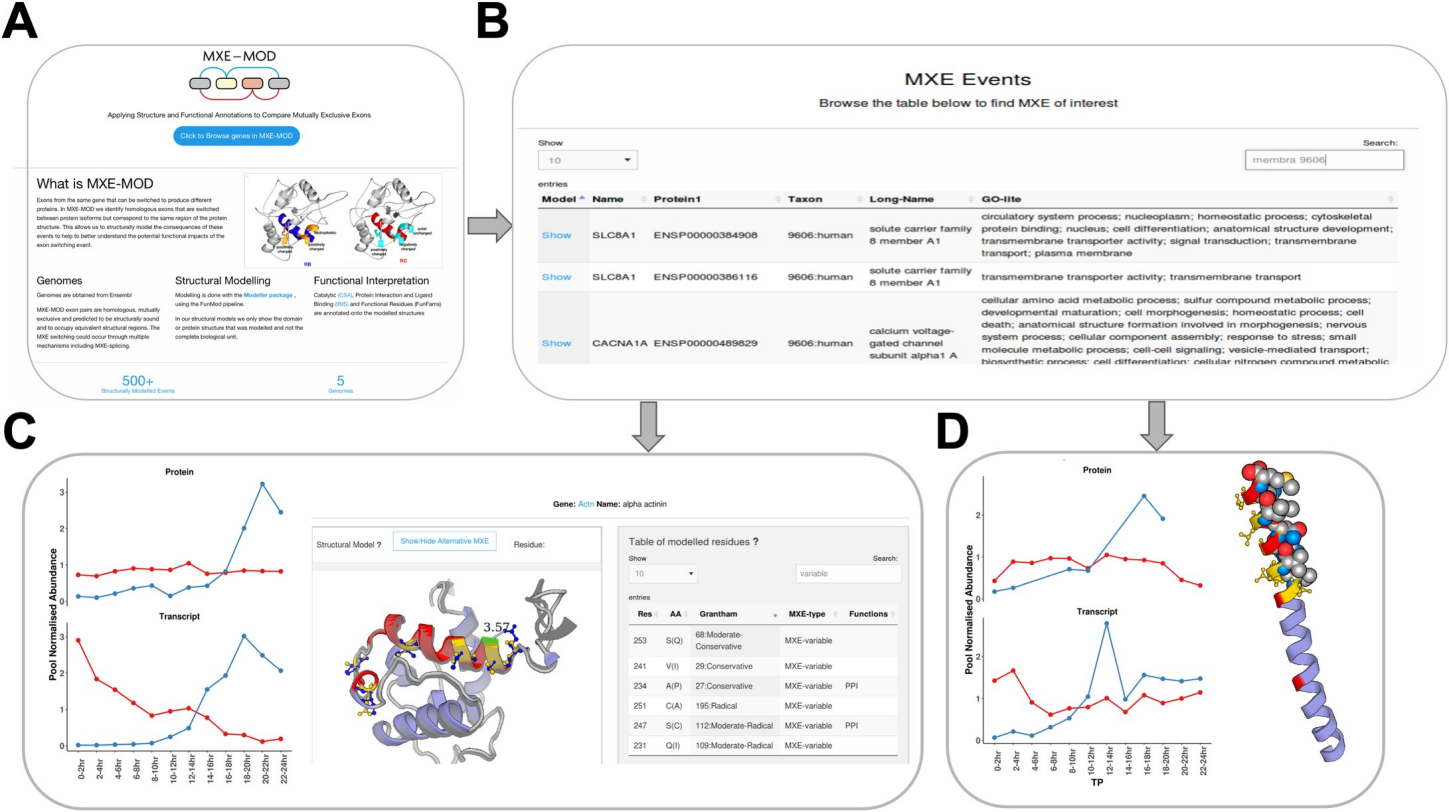
MXE-MOD Website. (A) The home page of MXE-MOD website. (B) The browser view allows the user to search different organisms and find a particular gene of interest. Annotations for the gene and associated MXE event have been integrated from different public resources: CATH, GENEONTOLOGY, Human Protein Atlas, DRUGBANK and Ensembl [31,46–49].(C-D) Detailed ‘MXE model’ pages for 2 different examples in the website. We can see the alternative MXE structure superposed in (C). We can see the variable PPI residues shown in space fill in the website in (D). MXE-MOD utilises the PV javascript viewer [50]. For both examples we show the expression patterns for the MXE isoform groups at different stages of development from RNA-seq and proteomics derived datasets. RNA-seq data is obtained from ModEncode resource [51] and proteomics data from the DDIP consortium (https://ddip-proteome.org) led by Simon Hubbard, Manchester University, UK.

To aid further research into MXE events and guide experimental studies, we developed a webserver called MXE-MOD available at http://gene3d.biochem.ucl.ac.uk/mxemod/. The website displays the MXE events on their modelled structures and shows the locations of functional residues in a simple and intuitive way (Fig 6A). From the website it is possible to browse the list of structurally modelled MXE events and view functional annotations from a number of data sources such as the Gene Ontology database and obtain information on the potential for druggability from the human proteome atlas [31] (Fig 6B).

The website can also be used to identify those isoform pairs in Drosophila, that switch gene expression strongly (i.e. having different RNA-seq expression patterns), via a previously published metric called the Transcript Switch score [45] which can help to identify further biological signatures of interest. In the following section, we highlight two MXE events with high Transcript Switch scores in Drosophila development. The first one alpha-actinin has a role in cytoskeleton protein binding and shows strong RNA-seq transcript isoform switching after dorsal closure (the proteome data shows a further lag in time for the switching) (Fig 6C). Clicking the superpose MXE button in the website shows that the alternative MXE has a highly similar structure but the physicochemical features of the variable side chains change (see contrasting gold and blue side chain colours). For this protein we can see there are six variable residues (coloured), two of which are involved in protein interactions of which one undergoes a non-conservative change (S247->C) and would likely alter PPI binding function. In a second example, Tropomyosin-1, a protein involved in actin binding, we see a strong transcript isoform switch around dorsal closure again resulting in remodelling of the PPI interface of the protein (Fig 6D).

## Discussion

Homologous mutually exclusive exons are enriched in proteomics data, suggesting a functional role. In this study, we used a large-scale structural modelling analysis to investigate how the homologous exons could be altering function. Previous analyses by other groups have demonstrated that MXE events are evolutionary conserved among vertebrates [17,18] and tend not to disrupt structural domains [17,18]. However, to date, there are no reported large-scale, structure-based studies in the literature exploring the likely structural, molecular, mechanistic and functional consequences of homologous MXE events that maintain structural integrity. In this work, we identified the homologous MXE events in human, mouse, fugu fish and zebrafish genomes using an in-house computational protocol. Most of these MXE genes are associated with membrane proteins (associated with cell-cell adhesion, signal transduction and molecule/ion transport). We annotated about 50% of the MXE events with structural information (i.e. known structures, structural models built using the CATH functional family based FunMod modelling platform and structural mapping) massively increasing the structural annotations available (i.e. by several hundred-fold). We also exploited our in-house CATH functional family resource to identify putative functional sites for proteins involved in MXE events, which significantly increased the amount of functional site data available thereby enabling a much greater, in-depth structure/function analysis than reported to date.

The fact that so many of the 3D modelled domains for MXE events produced reliable structural models (as judged by the structural quality scores such as the normalised DOPE score) suggests that the MXE sequence changes generally do not affect the stable folding of the proteins, but rather tune the functions of the proteins. Although this lack of disruption to the fold has been shown for individual structures previously [16], this is the first time it has been demonstrated using a large-scale structural modelling analysis. Previous structural analyses have also shown that MXE events tend to lie within structural domains (as opposed to being in e.g. intrinsically disordered regions) [18]. We want to highlight again that all the structures for the analysis are either complete structures from the PDB (though possibly protein subunits in their un-complexed form) or high quality well-packed 3D-models built from a 3D-template which overlaps significantly with the query sequence. Our analysis shows that the MXE events although being within domains, have average surface exposure far above what would be expected by chance. We would expect this phenomenon if the MXE events are associated with a change in function, as switching surface exposed residues can induce changes in protein functions (e.g. shifts in protein interfaces and binding pockets, etc.). Some MXE events are not so exposed and may affect buried allosteric sites or destabilise the protein. Furthermore, the variable residues tended to form clusters and generate large changes in physicochemical properties, demonstrating that MXE events can induce functional shifts in proteins.

We examined cancer mutations in an MXE structural context and focused on specific so-called cancer-driver domain families (i.e. MutFams, CATH functional families), which can be statistically associated with cancer mutations. We find a significant proximity (in structure) between cancer mutations in these families and the MXE variable residues, showing that cancer may dysregulate the same functional regions altered by MXE events.

Examination of the recently published MXE-splicing dataset (the Hatje dataset) from human (See S1 Text) showed similar trends. GO annotations enriched in both the Hatje and our main human MXE dataset are similar, both capturing largely membrane associated functions. The Hatje dataset is larger (it uses RNA-seq data to identify further transcripts) and is able to capture GO terms deeper in the hierarchy more easily, but the terms are similar to those for our main dataset at a higher level in the GO hierarchy. Regardless of the MXE dataset used, our structural modelling of the MXE events ensures that only MXEs that occupy equivalent regions of the structure and produce good structures are used in the analysis. Hence our dataset identifies MXE pairs that are homologous and occupy equivalent regions of the protein whilst maintaining the structural integrity of the protein.

Analysis of cassette exons and microexons showed none of the functional enrichments seen for homologous MXE datasets (See S2 and S3 Texts) highlighting a distinct functional role for MXEs. The case of microexons is interesting since they show several indicators of functionality including being on the surfaces of proteins, and as previously reported, they are under selective pressure to preserve the reading frame [8]. One caveat to our study to be kept in mind is that our analysis will necessarily be biased towards globular structural regions of proteins which are enriched in the PDB. In this analysis, we only analyse exons that map to ordered globular domain structures. MXE regions are more frequently (65%) found in ordered regions than cassette exons (18%). However, that does not exclude other possible functional effects of cassette exons. For example, cassette events that truncate the domain will effectively disrupt the protein structure and function.

Why MXE events have evolved in some proteins, altering their regulation or functional specificity, to give genes with separate functionalities, remains an open question, but for whatever reason it appears to be particularly important for functional innovations in membrane proteins. Similar mechanisms to those we describe for MXE events can occur in the functional divergence of paralogs [52] i.e. with mutation of residues lying in or near to existing functional sites. It could be argued that MXEs provide a conceptually simpler model for functional switching than would be needed to switch two functionally diverse paralogs, since for the latter, it would be necessary to fully stop transcription of the first paralog and transcribe the second paralog to obtain the alternative function whilst the MXE event is by definition a binary event. However, it must be noted that MXE events are far less frequent than gene duplication events and therefore will have a much smaller impact on the functional repertoire of the organism.

A detailed characterisation of MXE events can help us to better understand cell regulation, various diseases and possibly develop highly specific therapeutics. For example, one reported MXE event that could be targeted therapeutically is the MXE switch between 8a and 8 in the CaV1.2 Calcium Channel [53], which is implicated in multiple diseases and where there appear to be different sensitivities to the same drug, between isoforms. The fact that MXE regions are often in membrane proteins and/or surface exposed identifies them as promising targets for druggable genes. Unlike cassette exons where extensive structural disruption is likely (when occurring in a globular region) the MXE pairs maintain similar overall structures but with altered sidechains, which is ideal for facilitating targeted structure guided drug design to one isoform. Examples of druggable membrane proteins in our dataset includes important targets such as the sodium and calcium voltage-gated channels. Furthermore, there are additional benefits specific to targeting a splice variant. For example, if a drug binds efficiently to its target but the inhibition is too general (e.g. affecting many tissues), unwanted side effects can emerge. However, targeting a minor MXE splice variant could be advantageous since its expression will be much more (e.g. tissue) specific. A splice variant specific drug could target a subset of tissues/functions of the gene whilst leaving the more general function of the gene intact. A promising class of compounds for this are PROTACs which could be specifically engineered to target one splice variant for degradation [54].

A further advantage of MXE splice variants as drug targets is that, as we have demonstrated here, we can obtain reliable structural models for a considerable number of the MXE events, providing a good starting point for rational drug design. Furthermore, since the physicochemical properties of the variable residues show strong shifts between MXE events this should also help in designing isoform specific drugs. Finally, much of the above discussion for therapeutic drug development could equally be applied to developing more targeted insecticides, especially if some of the putative lineage-specific MXEs were validated (e.g. Multidrug-Resistance like Protein 1 in drosophila is one such MXE we identify).

## Materials and methods

### Identifying MXEs and the amino acid sequence region affected by MXE events

We downloaded the annotations of predicted MXEs for *Drosophila melanogaster* from the Kassiopeia resource [55] which corresponded to FlyBase 5.36 [56] and stored the MXE events and associated protein sequences that we could map to this genome. For example, we did not make use of non-reference genome exons and only used examples where the translations from chromosome sequence exactly matched the FlyBase translation. To help ensure we identified homologous MXE events, we filtered the fly MXEs to only include those with detectable homology (i.e. we compared the sequences against each other using BLAST [57], setting an e-value threshold of 0.005 and a minimum sequence identity of 25%). We also removed MXEs which involved nonsense-mediated mRNA decay transcripts. Note that, the fly DSCAM genes have a total of 4 variable exon clusters giving 38,016 potential splicing isoforms. Because of the dominance of the DSCAM gene in the drosophila dataset we removed it from further analysis so as not to bias the results too heavily towards one protein.

At the time of data preparation, the Kassiopeia resource did not provide MXE download annotations for the other four species (human, mouse, zebra fish and fugu fish). In order to identify MXEs for these species, we did the following. Based on Ensembl version 87 [49], we obtained the mRNA transcripts and identified sets of genes with MXEs. For each coding exon, we identified the longest transcript it was contained in and then compared this against all the other transcripts from the same gene to identify potential MXE. We made sure that the exons were consecutive in the DNA sequence, but never occurred together in the same transcript and couldn’t be linked together in a graph where edges were derived from any overlaps between exons in the gene. We restricted the exons to be similar in length (by checking that the absolute value of the log ratio of the MXE sequence lengths was > 0.25) and that they occurred in the same reading frame. After that, we translated all the MXE exons into amino acid sequences and compared the sequences against each other with BLAST, setting an e-value threshold of 0.005 and minimum sequence identity of 25%. Because we wanted to be more confident of the homology, we filtered out instances where either MXE was < 8 residues. Having at least 8 residues helped us to assess homology by BLAST more easily (i.e. with only a few residues this would not be possible). We allowed terminal exons amongst our MXE dataset and we did not enforce identical flanking exons, but 93% of our events had equivalent flanking exons (precise boundaries of these exons was allowed to vary). The structural modelling step ensures that the MXEs occupy equivalent regions of the structure and are valid structurally. Hence, our dataset focuses on the set of MXE pairs that are homologous and occupy equivalent regions of the protein whilst maintaining the structural integrity, rather than the exon switching mechanism and therefore differs from MXE splicing events defined in the Kassiopeia resource. However, during the preparation of the manuscript, a large and extensive set of MXE-splicing annotations became available for human (human validated-MXEs downloaded from the Kassiopeia website Hs_MXE_validated.gtf). We tested this additional dataset of RNA-seq validated MXE-splicing events (that we refer to as the Hatje dataset). For each of the MXE clusters in this file we identified one exon that mapped to a known coding exon (Using the gff3 file GRCh37.87 from Ensembl) and identified the longest protein sequence this exon was found in. We then picked an alternative exon from the MXE cluster and substituted this for its MXE partner to provide our alternative protein sequence. This step was carried out to be confident that the mapping of exons was done correctly and to obtain the full protein sequence. Furthermore, we sampled only one MXE-pair for each MXE cluster (to prevent biases from large MXE clusters affecting our analysis). Note as part of our pipeline we filtered the Hatje dataset using the BLAST criterion defined above since we are only reliably able to do the homology model comparisons by enforcing this. The Hatje dataset with RNA-seq and more MXE-splicing splicing filters [18] therefore provides an important comparison as to our dataset. Applying our structural modelling pipeline ensures, in both datasets, the MXE pairs are homologous and occupy equivalent regions of the protein whilst maintaining the structural integrity of the protein.

### Orthologs

Orthologs were obtained using Ensembl Compara [58] including all orthology relationship types. This was to make sure we were not being overly conservative, since the main purpose of this step was to check for any potential redundancy (at the ortholog level) between genomes.

### Predicting the function of MXE genes

We used the PANTHER functional annotation tool, last updated in February 2020, to identify functional enrichments of the MXE genes. We used 5 different background sets (organism whole genome/ genes that have no paralogs/ multi-exon genes/ multi-protein isoform genes/ MXE or CE genes) for testing the enrichment.

### Annotating MXE events with structural information

We mapped the splice isoforms to CATH functional families (FunFams). FunFams are a sub-classification of CATH protein domain superfamilies [46]. Each FunFam groups together relatives likely to have very similar structures and functions. They are generated using the FunFHMMer protocol [25] that detects similarities in sequence patterns (highly conserved positions and specificity determining positions). Positions that are highly conserved across a superfamily are generally important for the stability, folding or function of the protein domain. Specificity-determining positions are positions that are conserved within and unique to a particular FunFam, responsible for a specific function and usually involved in functional divergence from other FunFams. The FunFam protocol uses agglomerative clustering to iteratively merge clusters of relatives. This is done by starting with very close homologues (>90% sequence identity) and building multiple sequence alignments (Katoh and Standley, 2013) and Hidden Markov Models (using HMMER [59]) for each cluster. Subsequently all vs all, HMM-HMM comparisons are performed across all the clusters using HHpred [60] and the most similar clusters merged. A new MSA and HMM are built for the merged cluster and another round of HMM-HMM comparisons performed. This is repeated until all clusters have been merged giving a hierarchical tree for the superfamily. Finally, the tree is used to guide the identification of distinct functional family clusters having different specificity determining residues from other clusters in the tree. More comprehensive details of the functional family generation protocol can be found in [25].

The domains within a given FunFam have been demonstrated to be structurally coherent [26,61]. The functional purity of the FunFams has been demonstrated by validating against experimentally determined proteins from the Enzyme Commission and also by checking whether known functional sites coincide with highly conserved residues in the Multiple Sequence Alignments (MSAs) of FunFams [25]. CATH FunFams have been shown to be more functionally pure than Pfam domain families [25,26]. Functional predictions based on FunFams were ranked amongst the top five methods for the "Molecular Function" category and the "Biological Process" category in the most recent CAFA International Function Prediction experiments (CAFA2, [62], CAFA3, [63]).

All the MXE events were scanned against the library of CATH v4.1 FunFam HMMs [46,64] using HMMER [59]. DomainFinder3 [65] was used to determine which CATH-Gene3D FunFams they belonged to. We only considered matches with a HMMER E-value of less than 0.001. For FunFams that had a known domain structure, we annotated the MXE events using the FlyBase/Ensembl to PDB mapping. For FunFams that had no relative of known structure, we used the FunMod modelling pipeline [61,66] which exploits the MODELLER [67] algorithm to build structural models. We ensured that the sequence to be modelled overlapped with 80% of the residues in the representative sequence for the FunFam. We used normalised DOPE [68] and GA341 [69] to assess the quality of the models. Only good quality models with a negative normalised DOPE score and a GA341 score of more than 0.7 were included in this analysis. For those MXE events where we failed to build a model, we mapped them to the structural representative of the respective FunFam. To ensure that we chose a structural representative that represents the FunFam well, the structural domain with the highest cumulative SSAP structural similarity score and the best X-ray resolution was used. SSAP is a well-established structural comparison method [70]. The SSAP score ranges from 0 to 100, with a score of 100 for identical structures.

The two splice isoforms for an MXE event were aligned to other relatives in the FunFam using the multiple sequence alignment tool MAFFT [71] and the function mafft-add. We extracted the alignment between the structural representative of the FunFam and the two splice isoforms. Based on this alignment, we extracted the variable residues between the two isoforms (See Fig 1).

For those MXE events which are not included in FunFams, we scanned the isoforms against the libraries of HMMs built from non-redundant structures in various resources, CATH v4.1 HMMs (i.e. including non-FunFam CATH domains), SCOPe 2.06 HMMs [72] and PDB70 June 2017 HMMs [73]) using either HMMER or HHsearch [60]. Similarly, we only considered matches with an E-value of less than 0.001. We used the FunMod modelling platform to build structural models. For those events where we failed to build a good model, we mapped them to the best structural matches (based either on the HHsearch or the HMMER result). We only analyse MXE events where both isoforms map to the same structural template. We aligned the isoforms with the structural template using MAFFT and extracted the variable residues between the two isoforms.

For those MXE events which we failed to annotate with structure information, we predicted if they were intrinsically disordered using IUPRED [74]. We used the long-disorder option of IUPRED and residues with IUPRED score above 0.5 were considered disordered. We defined a splice MXE isoform as intrinsically disordered if more than 50% of the residues were predicted to be disordered.

### Identifying single and minimal sequence loss Cassette exon events

To compare the MXE events with other types of alternative splicing we examined the impacts of single cassette exon events and microexon events, both of which like MXE events would be expected to have a relatively small effect on the protein structure compared to other AS events.

Loss of exons is likely to disrupt protein structure, especially if the loss is in the globular folded region (which is mainly the region that our structural modelling is restricted to). By restricting to single exon events, we hoped to identify cassette exons that were least likely to alter protein structure and should therefore be easier to analyse from a structural point of view. Conversely, we can imagine that in general more extreme splicing events leading to greater losses would be more disruptive to structure and more difficult to analyse from a homology modelling point of view. To minimise this, for each protein coding gene we took the longest protein isoform and found the next longest isoform where the only difference was the loss of a single coding, non-terminal exon. In this way, we were more likely to identify a minimally disruptive cassette exon event. We used this set of minimally disruptive cassette exons for the domain/ protein function enrichments and the cassette exon structural models. We only analysed cassette exon events that could be mapped to a globular domain in one of our FunFams.

### Microexons

Microexons have been defined previously as having ≤27 nucelotides (nt). The most comprehensive set of microexons can be found in the VASTDB database [75]. We obtained our microexon sequences by extracting exons with “EX” in the VASTDB identifier (i.e. HsaEX*) and with length ≤ 27 nt (September 2019). Where possible we mapped the nucleotide sequences using microexon and flanking exons to identify corresponding proteins from CDS data in Ensembl genomes allowing us to place the microexon in the full-length protein sequence.

### Quantifying the residue changes using a physicochemical score

We compared the physicochemical properties of variable residues using the McLachlan physicochemical similarity matrix [32]. A pair of amino acids was given a similarity score ranging from 0 to 6. A score of 0 indicates no similarity or a deletion. The score for a pair of identical amino acids is typically 5 or 6 [32]. For each MXE, we summed up all the McLachlan similarity scores for a set of variable residues and divided by the total number of mapped variable residues to normalise the score.

### Analysis to determine if variable residues in the splice event are exposed to solvent

NACCESS [76] is a stand-alone program that calculates the relative accessible surface (rASA) of amino acids in a PDB structure. For each MXE event, we calculated the rASA for all the corresponding variable residues of the two isoforms. Amino acid residues were considered to be exposed if the rASA value was above 10%. We compared the solvent exposure of the variable residues versus the solvent exposure of the MXE conserved residues for each MXE event. The solvent exposure was calculated as:

$$solvent\;exposure=\frac{Number\;of\;exposed\;residues}{Total\;number\;of\;residues}$$

We also investigated random models to examine the solvent exposure for the splice region itself relative to background. For every MXE pair, we randomly selected from the same structure regions that have the same size as the splice region, 10,000 times, and determined the percentage of exposed residues in this region. This percentage is then compared with the splice region solvent exposure. We used the Wilcoxon signed-rank test to compute the statistical significance.

### Analysis to determine if variable residues in MXE regions are clustered in 3D and are close to functional sites

We investigated whether exposed variable residues in MXE events lie on or in the vicinity of functional residues. To reduce the noise and identify those variable residues likely to be having similar impacts, we clustered variable residues into structural clusters. For each pair of MXE events, we calculated the all-atom-versus-all-atom distances of the variable residue atoms of the structural representative and determined the minimum distance between residues. We used an in-house multi-linkage clustering program which clusters residues that are within 8Å distance of each other (i.e. between the centres of the residues). We made sure there are at least three residues present in a cluster.

Subsequently, we calculated the distance from the structural clusters to known functional residues of the structural representative. We calculated the centre-of-mass of the clusters. The minimum distance from the centre-of-mass of the cluster to the closest functional residue was calculated. If the functional residue was in the cluster the distance was simply set to zero. We used a distance cut-off of 6Å to define if the structural cluster lies close to a functional site.

We also investigated random models to examine the proximity of randomly selected residue regions to functional sites, to evaluate statistical significance. For every MXE pair, we randomly selected structural clusters that have the same size as the largest MXE structural cluster, 10,000 times, and determined the percentage of clusters that lie close to functional sites. This was done by randomly selecting a residue and then identifying the residues that are close (less than or equal to 2Å) to the selected residue, and each other, until they made up the same volume as the largest MXE cluster. Then, the overall percentage for all random events was computed. This percentage is then compared with the actual MXE percentage. We used the Z-score test to compute the statistical significance.

The functional sites considered were known experimentally characterised sites: catalytic residues taken from CSA [21], protein-protein interaction residues and protein-small molecule interaction residues (small molecule binding) taken from IBIS [77]. We also used our in-house predicted functional sites, FunSites [25], and predicted allosteric sites. FunSites are highly conserved positions in multiple sequence alignments of the FunFam relatives, not conserved across the whole superfamily. For the prediction of FunSites, we only perform this analysis for FunFams that have a diverse set of relatives making it possible to distinguish conserved positions from variable positions. The Scorecons method [78] was used to calculate the sequence diversity of a FunFam alignment by generating a diversity of positions score (DOPS score). FunFams with a DOPS score of above 70 (out of 100) are deemed sufficiently sequence diverse for analysis (i.e. having a lower probability of predicting false positives for FunSites [26]).

We also used the A-SITE allosteric site predictor (Dr Aurelio Moya Garcia, personal communication), based on the centrality of nodes (in a protein i.e. residue nodes) in terms of their capability to transfer signals through the protein, to predict allosteric sites in the protein, in order to determine whether variable residues lay on or close to allosteric sites. The program identifies residues with high betweenness centrality. The depletion of residues having high betweenness centrality values would be expected to interrupt the allosteric communication among regions of the protein that lie far apart. For each structure, we defined its allosteric residues as the top 5 percentile residues (ranked by the A-SITE program using the betweenness centrality measure). The source code for A-SITE program is available at https://github.com/amoyag/protein-structure-network.

### Analysis to determine if variable regions in human MXE pairs are close to COSMIC cancer mutation residues

COSMIC is a database which collects somatic mutations from human cancer patients [79]. Accumulation of these mutations may alter cellular functions and contribute to cancer [80]. A number of studies have found that cancer mutation sites lie in the vicinity of functional residues (i.e. protein and small molecule binding sites) [33,34].

Recently, mutationally enriched domain functional families (MutFams) have been identified by in-house studies [24]. MutFams are CATH functional families that have been found to be statistically significantly enriched in somatic missense cancer mutations reported in COSMIC. They are identified using a protocol developed by Miller et al. [81] that searches for significant enrichment of cancer mutations in a specific domain, compared to the protein background, for the set of proteins containing the specific domain family. A total of 541 pan-cancer MutFams were identified and they indicate important cancer domain families such as P53 and PTEN [24]. MutFam putative driver genes were found to have reasonable overlap with driver genes identified from Cancer Gene Census and genes identified by other methods based on Pfam families. MutFam genes are enriched in survival and cell-motility cancer hallmark processes and additionally identify proteins with G1/S checkpoint function in DNA repair [24]. Pan-cancer mutations clustered on representative domains in MutFams with available structural domains, have been found to be significantly closer to key functional sites than un-clustered cancer or germline disease mutations.

Therefore, we determined if MutFam cancer mutation residues lie close to MXE variable residues. We mapped all the MutFam mutation residues onto the human MXE events. For those where we have structural information, we determined if the MXE variable residue or residue clusters are close to MutFam mutation residues using a distance cut-off of 4Å (between any atoms in the residue pairs) or 6Å (to the centre of the MXE cluster).

### Druggable / Tissue gene enrichments

The set of FDA druggable proteins downloaded from the Human Protein Atlas (HPA) and we checked for enrichment against the gene lists from the MXE datasets. The gene background for the Fisher Exact test was set to the set of protein coding genes. The TopAnat enrichments were calculated through the website using the default parameters (node size = 20, all-datasets (gold and silver)) but using only protein coding genes (from GRCh38) as the genomic background and we report the FDR scores from the website, as described in the methods publication [28,29].

### Additional tools used to analyse specific examples

We used Adaptive Poisson-Boltzmann Solver (APBS) [36] to determine the surface potentials of two MXE regions. APBS is a software package which models biomolecular solvation by solving the Poisson-Boltzmann equation (a model used to describe electrostatics interactions between solutes in salty, aqueous media). We used mCSM [37] and mCSM-lig [82] to assess the effect of the MXE variable residue changes on protein-protein affinity and protein-small molecule binding affinity. Both methods use graph-based signatures (distance patterns between atoms and are used to represent the protein residue environment) to train a predictive model from a representative dataset.

## Supporting information

S1 Fig MXE statistically enriched CATH superfamilies. For every CATH superfamily identified in each species, we obtained the number of MXE genes mapped to the superfamily, the number of MXE genes that do not map to the superfamily, the number of non-MXE genes mapped to the superfamily and the number of non-MXE genes that do not map to this superfamily. This information was then used to compute a 2x2 contingency table. We then performed the Fisher exact test using the contingency table. We adjusted the p-value using Benjamini-Hochberg correction to account for multiple hypothesis testing. We check the odds ratio is > 1 and p-value < 0.05 for significance. MXE domain families are enriched in important Metazoan functions such as membrane proteins involved in cell-cell adhesion and signal transduction.
(TIF)

S2 Fig Enriched biological annotations identified by PANTHER for all MXE genes. We used organism whole genome as background. We were unable to include Fugu fish because PANTHER does not include Fugu fish in their analysis datasets.
(TIF)

S3 Fig Enriched biological annotations identified by PANTHER for MXE genes that have no paralogs. For our background dataset, we only used genes that have no paralogs. Removing the paralogs was to counter potential effects from MXE events being retained after a gene duplication, which could lead to overestimates of functional coherence of the gene set. We were unable to include Fugu fish because PANTHER does not include Fugu fish in their analysis datasets.
(TIF)

S4 Fig Enriched biological annotations identified by PANTHER for MXE multiexon genes. We used only multi-exon genes as background. Removing single exon genes was to counter potential effects of any functional bias, since by definition our MXE genes require more than one exon. We were unable to include Fugu fish because PANTHER does not include Fugu fish in their analysis datasets.
(TIF)

S5 Fig Enriched biological annotations identified by PANTHER for MXE multi-protein isoform genes. We used only multi-protein isoform genes as background. Removing single protein isoform genes was to counter potential effects of any functional bias. We were unable to include Fugu fish because PANTHER does not include Fugu fish in their analysis datasets.
(TIF)

S6 Fig Enriched biological annotations identified by PANTHER for MXE genes. We used only MXE or CE genes as background to counter potential effects of any functional bias. We were unable to include Fugu fish because PANTHER does not include Fugu fish in their analysis datasets.
(TIF)

S7 Fig Splice isoforms with structural information. (TIF)

S8 Fig Physicochemical properties of MXE variable residues versus randomly sampled residues within the same threshold distance of 4Å from a particular class of functional residue (catalytic, ligand binding, PPI). For each FunFam, with high DOPS, and a known structure we took all known functional sites (CSA (catalytic residues), PPI (protein-protein interactions), PSI (protein-small molecule Interactions)) and identified other residues within 4Å from the functional site. We then determined the residue usage and calculated the chemical changes using McLachlan score. We used Mann-Whitney U test to compute the statistical significance.
(TIF)

S9 Fig Number of structural clusters formed by MXE variable residues. (TIF)

S10 Fig Proportion of variable region clusters in MXE events that lie close to functional sites compared to the number of random clusters that lie close to functional sites, for the 5 model organisms analysed. (TIF)

S11 Fig Proportion of variable residues in MXE events that lie close to functional sites compared to the number of random residues that lie close to functional sites for the 5 model organisms analysed. For every pair of MXE events, we determined if the variable residues within the splice regions lie close to any functional sites. We calculated the minimum distance between the atoms of the residues involved. We used a distance cut-off of 4Å to determine if residues are close. For every pair of MXE events, we created 10000 random models (based on the patterns of location of variable residues (i.e. X-X---X-X-X, X, variable residues, -, non-variable residues), and determined the percentage of highlighted residues that lie close to functional sites. We used the z-score test to compute the statistical significance. This reports a p-value for the level of significance. There is a statistically significant tendency for MXE events with variable residues to lie close to protein-protein interaction, protein-small molecule and FunSites for both fly and human datasets. There is also a tendency for variable residues to be in the vicinity of allosteric sites for the fly dataset. We had less than 10 events annotated with catalytic sites but these did not usually lie close to a MXE region using a 4Å distance cut-off.
(TIF)

S12 Fig Enriched biological annotations for cancer-related human MXE genes identified by PANTHER. We used organism whole genome as background.
(TIF)

S13 Fig PKM2 and PKM1. (A) Exon structures of PKM isoforms. (B) Sequences from domain 4 for the 2 isoforms. The splice regions are coloured purple or yellow. (C)The sequence alignment of the splice region. Variable residues are coloured as red.
(TIF)

S14 Fig The splice region and surface potential of PKM1 and PKM2 (with FBP bound). The FBP molecule is coloured in cyan.
(TIF)

S15 Fig Summary of the mCSM predicted changes in stability for MXE residue changes (from PKM1 isoform to PKM2 isoform) located near the protein interface using PKM1 structure (PDB id 3SRF). (TIF)

S16 Fig Summary of the mCSM predicted changes in stability for MXE residue changes (from PKM2 isoform to PKM1 isoform) located near the protein interface using PKM2 structure with FBP bound (PDB id 1T5A). (TIF)

S17 Fig mCSM-lig predicted the ligand binding affinity for FBP binding residue 539 (from valine to isoleucine) to be more stabilising. The analysis was done using PFKP structure (PDB id 4XZ2).
(TIF)

S18 Fig The change in surface potential between CRMP MXE splice isoforms. Positively charged residues are coloured as blue, negatively charged residues are coloured as red.
(TIF)

S19 Fig General statistics of the Hatje dataset. (A) Distributions of MXE sizes. (B) Sequence identity between MXE pairs. (C) Number of variable residues between MXE pairs.
(TIF)

S20 Fig Enriched biological annotations identified by PANTHER for Hatje MXE genes. We used organism whole genome as background.
(TIF)

S21 Fig Enriched biological annotations identified by PANTHER for Hatje MXE genes that have no paralogs. For our background dataset, we only used genes that have no paralogs. Removing the paralogs was to counter potential effects from MXE events being retained after a gene duplication, which could lead to overestimates of functional coherence of the gene set.
(TIF)

S22 Fig Enriched biological annotations identified by PANTHER for Hatje multiexon genes. We used only multi-exon genes as background. Removing single exon genes was to counter potential effects of any functional bias, since by definition our MXE genes require more than one exon.
(TIF)

S23 Fig Enriched biological annotations identified by PANTHER for Hatje multi-protein-isoform genes. We used only multi-protein-isoform genes as background. Removing single protein-isoform exon genes was to counter potential effects of any functional bias, since by definition our MXE genes require more than one exon.
(TIF)

S24 Fig Enriched biological annotations identified by PANTHER for Hatje genes. We used only MXE or CE genes as background to counter potential effects of any functional bias.
(TIF)

S25 Fig Structural and functional analysis of the Hatje MXE splicing dataset. (A) Proportion of splice isoforms with structural information. (B) Distribution of McLachlan scores for the variable MXE residues. (C) The surface exposure of the MXE events compared to random expectation. (D)The surface exposure of the MXE variable residues compared to the MXE event as a whole. (E) Proximity of MXE variable residue clusters to amino acids assigned to different functional classes (indicated by Icons) CSA = catalytic residues from the Catalytic Site Atlas, PPI = protein interaction sites and PSI = protein-small molecule interactions).
(TIF)

S26 Fig General statistics of cassette exon dataset. (A) Number of cassette exons identified (B) Distribution of cassette exon sizes. (C) Distribution of cassette exon coverage (whole sequence).
(TIF)

S27 Fig Enriched biological annotations for CE genes identified by PANTHER. We used organism whole genome as background.
(TIF)

S28 Fig Enriched biological annotations for CE genes that have no paralogs. For our background dataset, we only used genes that have no paralogs. Removing the paralogs was to counter potential effects from MXE events being retained after a gene duplication, which could lead to overestimates of functional coherence of the gene set.
(TIF)

S29 Fig Enriched biological annotations for CE multiexon genes identified by PANTHER. We used only multi-exon genes as background. Removing single exon genes was to counter potential effects of any functional bias, since by definition our MXE genes require more than one exon.
(TIF)

S30 Fig Percentage of the CATH-Gene3D functional family domain deleted in the shorter isoform. (TIF)

S31 Fig Model quality difference between the longer isoform and the shorter isoform. We only show cases where an acceptable quality model can be built for the longer isoform.
(TIF)

S32 Fig nDOPE score difference of CE and MXE structural models. The difference in nDOPE score is given by a ΔnDOPE value (by subtracting the nDOPEs of the two isoforms and then taking the absolute value).
(TIF)

S33 Fig Proportion of deleted regions in CE events that lie close to functional sites, compared to the number of random clusters that lie close to functional sites. (TIF)

S34 Fig General statistics of MIC dataset. (A) Number of MIC events identified (B) Distributions of MIC size.
(TIF)

S35 Fig Enriched biological annotations for MIC genes identified by PANTHER. We used organism whole genome as background.
(TIF)

S36 Fig Enriched biological annotations for MIC genes that have no paralogs. For our background dataset, we only used genes that have no paralogs. Removing the paralogs was to counter potential effects from MXE events being retained after a gene duplication, which could lead to overestimates of functional coherence of the gene set.
(TIF)

S37 Fig Enriched biological annotations for MIC multiexon genes identified by PANTHER. We used only multi-exon genes as background. Removing single exon genes was to counter potential effects of any functional bias, since by definition our MXE genes require more than one exon.
(TIF)

S38 Fig Structural and functional analysis of MIC splicing dataset. (A) Percentage of splice isoforms with structural information. (B) The surface exposure of the MIC residues compared to non-MIC residues.
(TIF)

S39 Fig Proportion of MIC regions that lie close to functional sites, compared to the number of random clusters that lie close to functional sites. (TIF)

S40 Fig Proportion of neural-regulated MIC regions that lie close to functional sites, compared to the number of random clusters that lie close to functional sites. (TIF)

S1 Text The following section summarises the results analysing MXE events from the Hatje dataset. (DOCX)

S2 Text The following section reports results obtained for the cassette exon analysis. (DOCX)

S3 Text The following section reports results obtained for the microexon analysis. (DOCX)

S4 Text The following sections give expanded details on the structural analysis of examples presented in the main text with some more details on methods. (DOCX)

S1 Table List of MXE genes. (XLSX)

S2 Table List of druggable MXE targets. (XLSX)
