# Supplementary figures and images for: Biological impact of mutually exclusive exon switching
Source: PLoS Comput Biol. 2021 Mar 2;17(3):e1008708. doi: 10.1371/journal.pcbi.1008708 (PMC7954323; doi:10.1371/journal.pcbi.1008708)

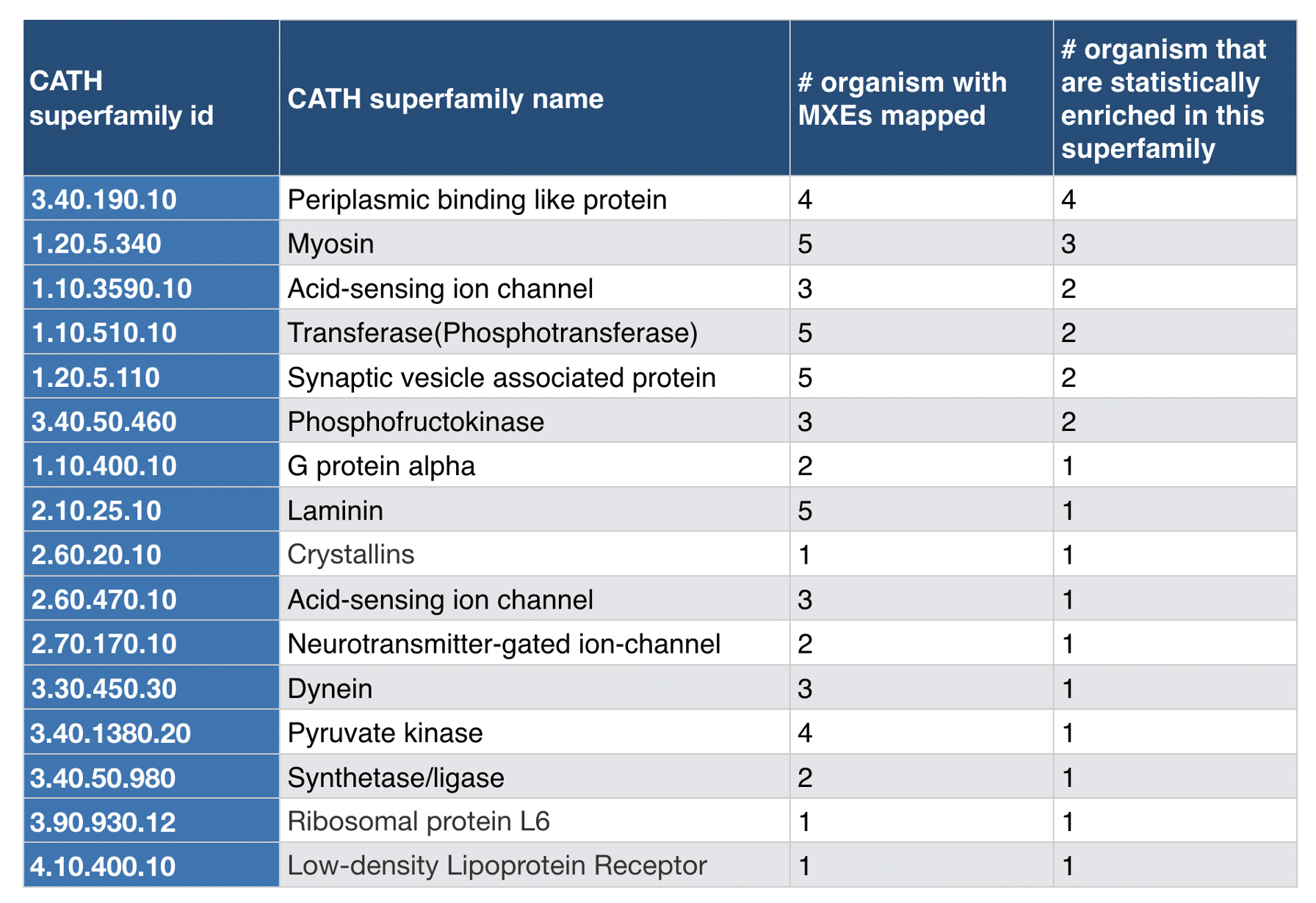

Supplement: S1 Fig — For every CATH superfamily identified in each species, we obtained the number of MXE genes mapped to the superfamily, the number of MXE genes that do not map to the superfamily, the number of non-MXE genes mapped to the superfamily and the number of non-MXE genes that do not map to this superfamily. This information was then used to compute a 2x2 contingency table. We then performed the Fisher exact test using the contingency table. We adjusted the p-value using Benjamini-Hochberg correction to account for multiple hypothesis testing. We check the odds ratio is > 1 and p-value < 0.05 for significance. MXE domain families are enriched in important Metazoan functions such as membrane proteins involved in cell-cell adhesion and signal transduction. (TIF) [file pcbi.1008708.s001.tif]

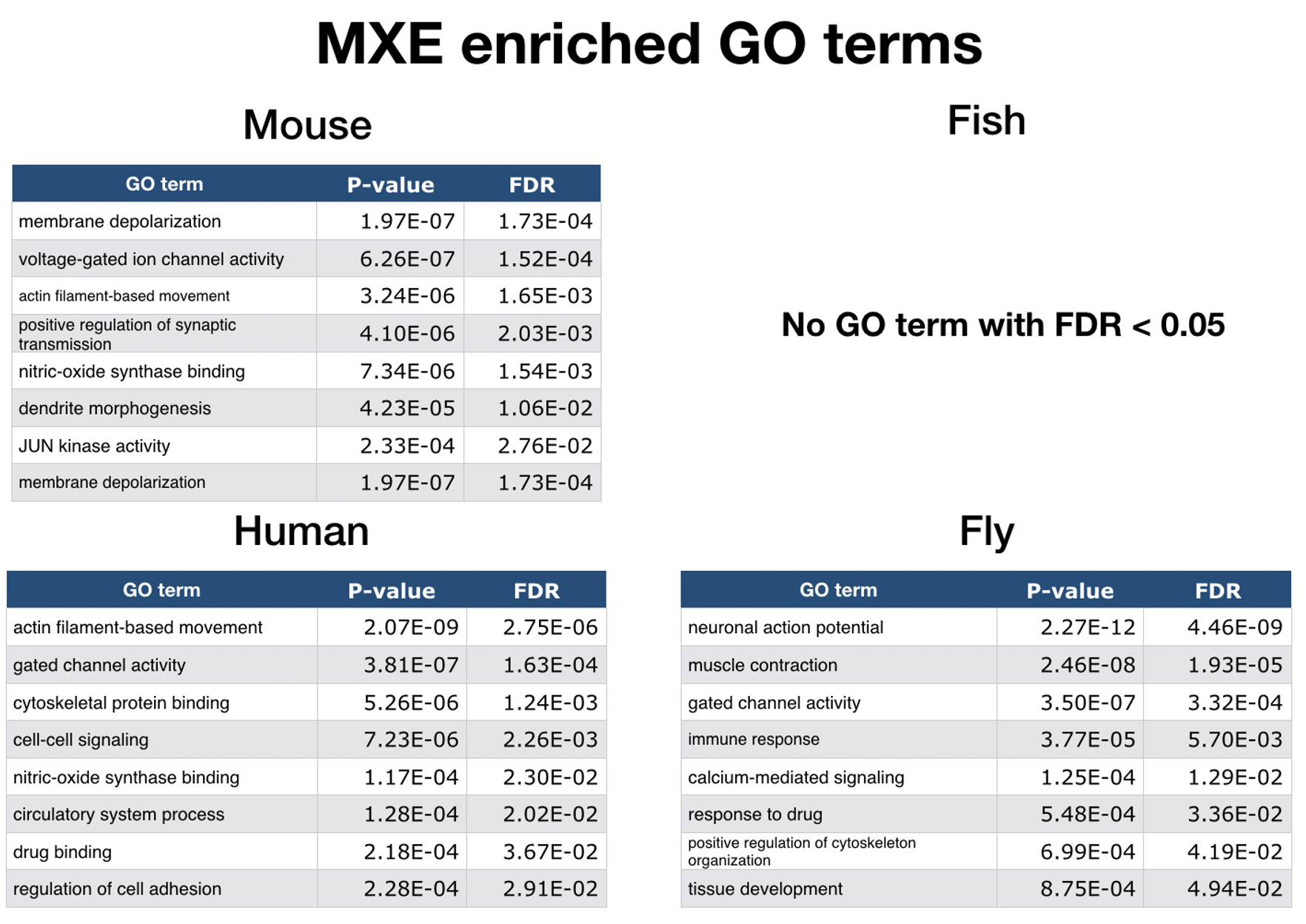

Supplement: S2 Fig — We used organism whole genome as background. We were unable to include Fugu fish because PANTHER does not include Fugu fish in their analysis datasets. (TIF) [file pcbi.1008708.s002.tif]

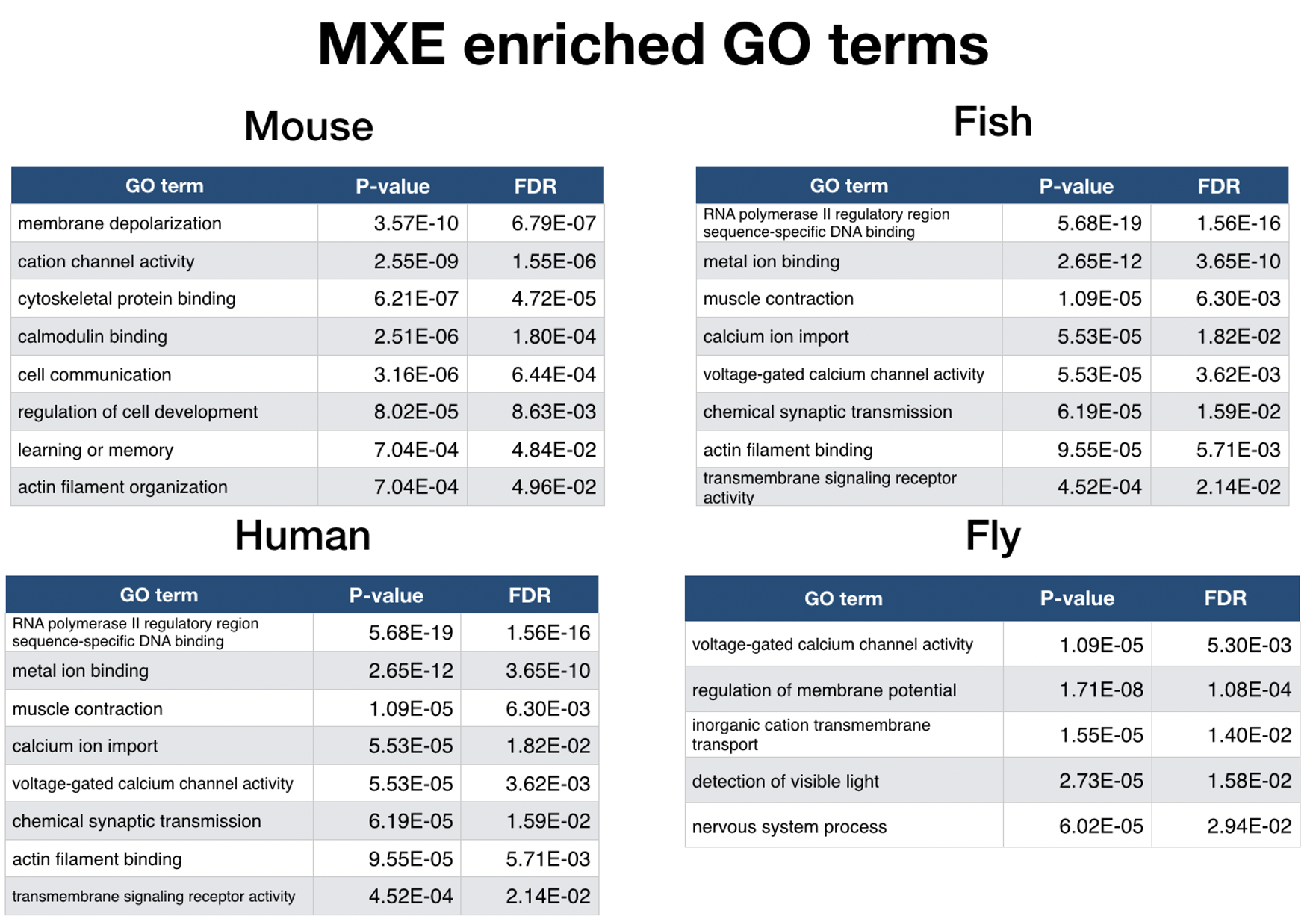

Supplement: S3 Fig — For our background dataset, we only used genes that have no paralogs. Removing the paralogs was to counter potential effects from MXE events being retained after a gene duplication, which could lead to overestimates of functional coherence of the gene set. We were unable to include Fugu fish because PANTHER does not include Fugu fish in their analysis datasets. (TIF) [file pcbi.1008708.s003.tif]

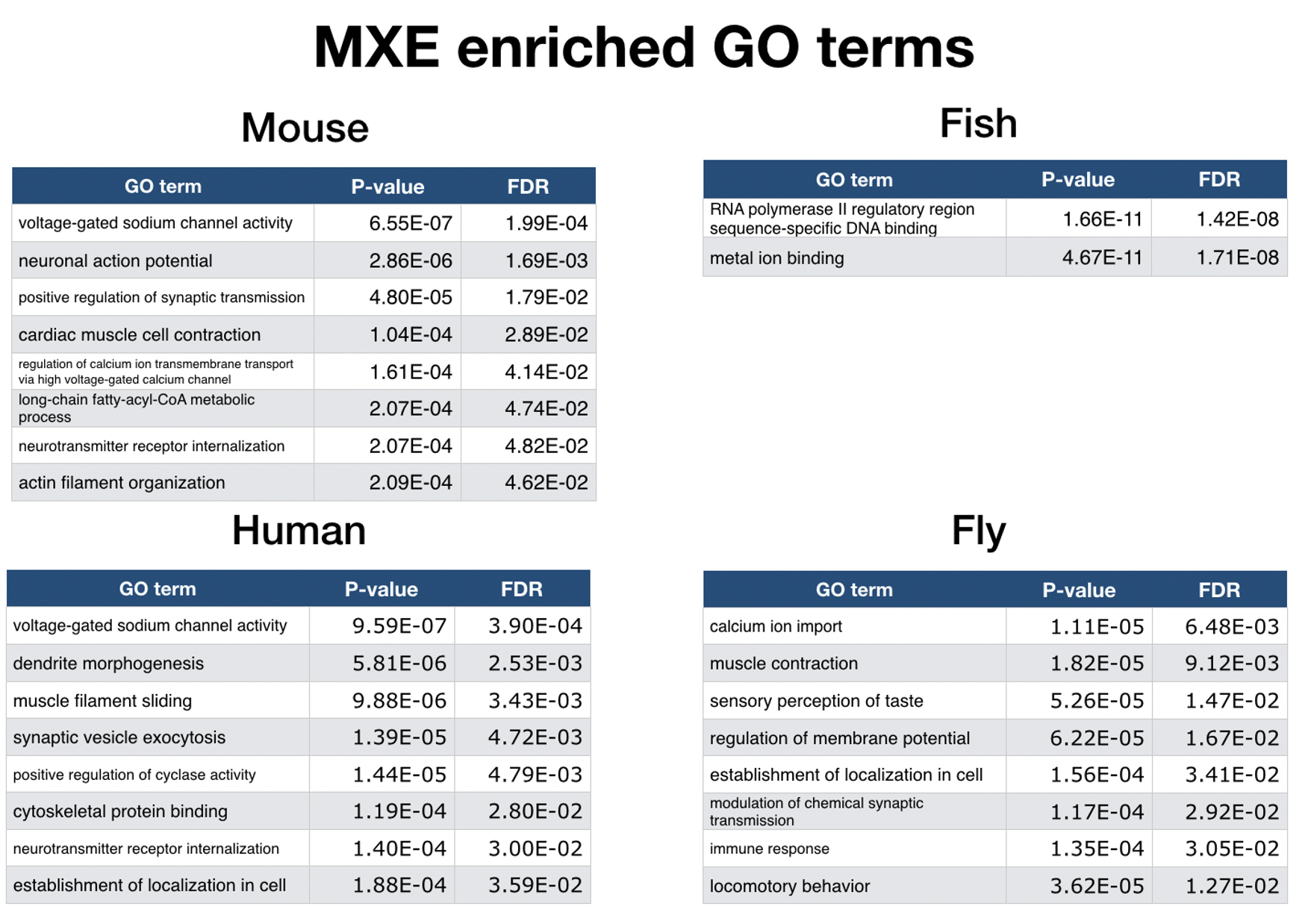

Supplement: S4 Fig — We used only multi-exon genes as background. Removing single exon genes was to counter potential effects of any functional bias, since by definition our MXE genes require more than one exon. We were unable to include Fugu fish because PANTHER does not include Fugu fish in their analysis datasets. (TIF) [file pcbi.1008708.s004.tif]

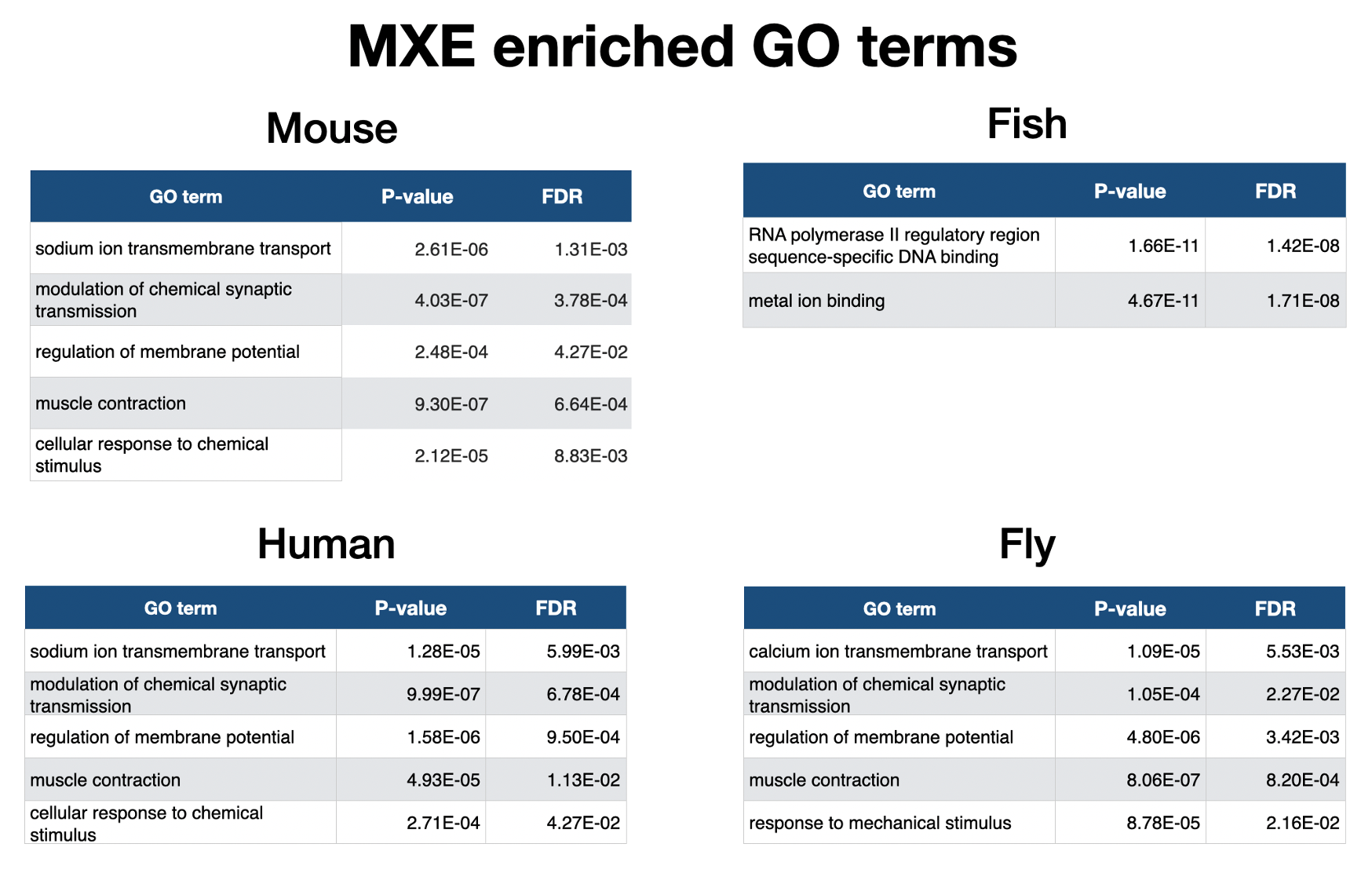

Supplement: S5 Fig — We used only multi-protein isoform genes as background. Removing single protein isoform genes was to counter potential effects of any functional bias. We were unable to include Fugu fish because PANTHER does not include Fugu fish in their analysis datasets. (TIF) [file pcbi.1008708.s005.tif]

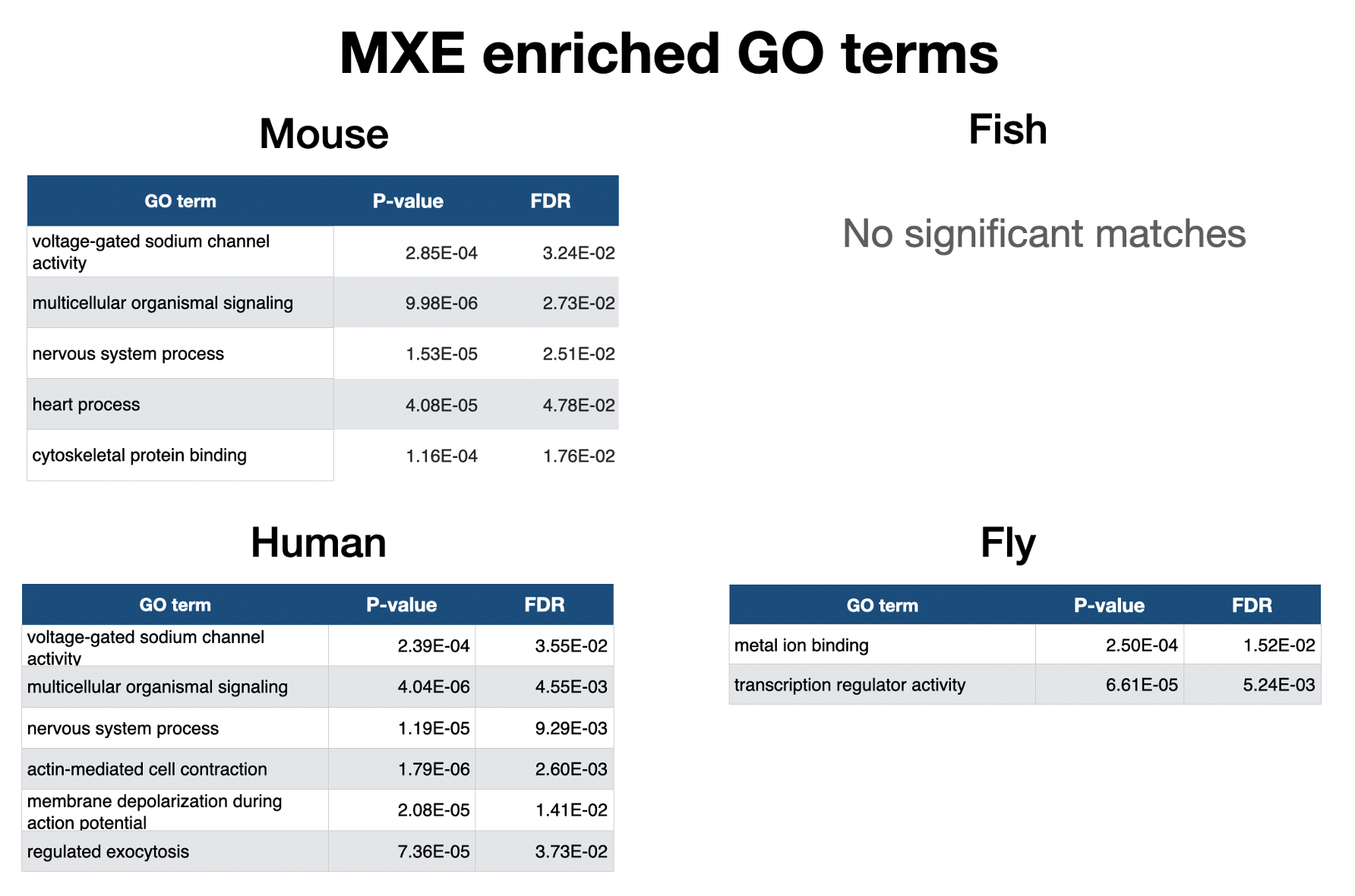

Supplement: S6 Fig — We used only MXE or CE genes as background to counter potential effects of any functional bias. We were unable to include Fugu fish because PANTHER does not include Fugu fish in their analysis datasets. (TIF) [file pcbi.1008708.s006.tif]

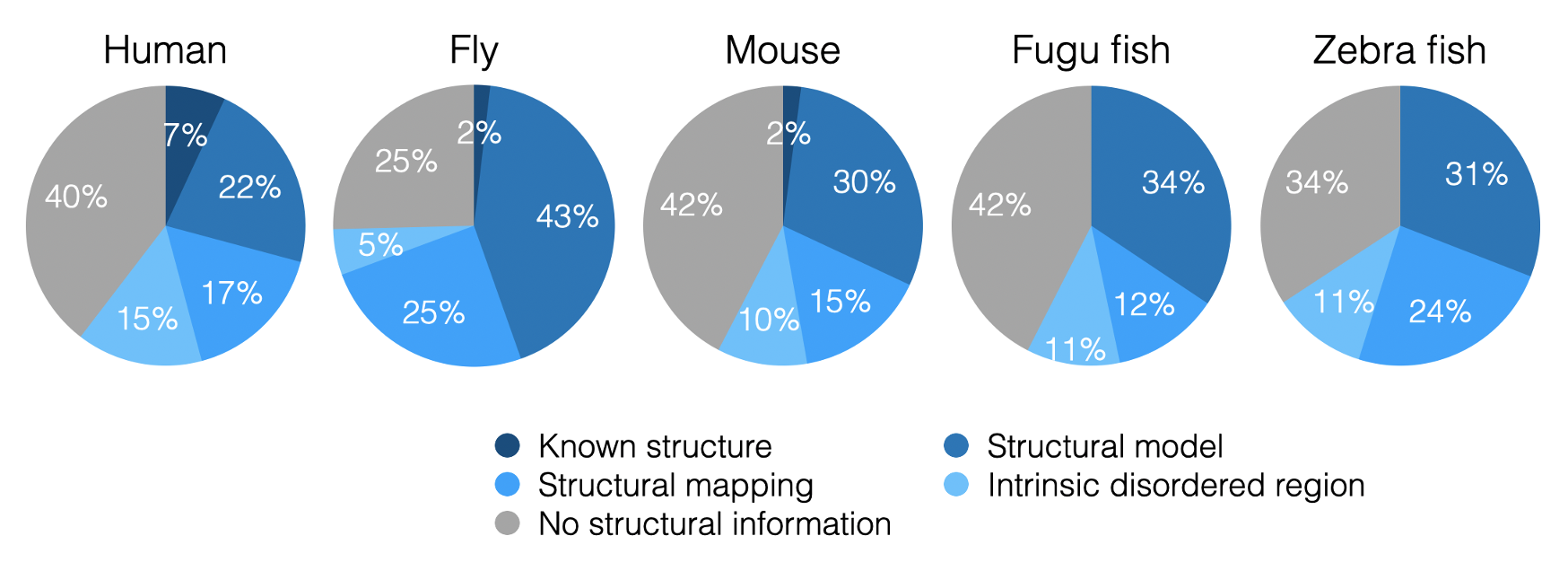

Supplement: S7 Fig — (TIF) [file pcbi.1008708.s007.tif]

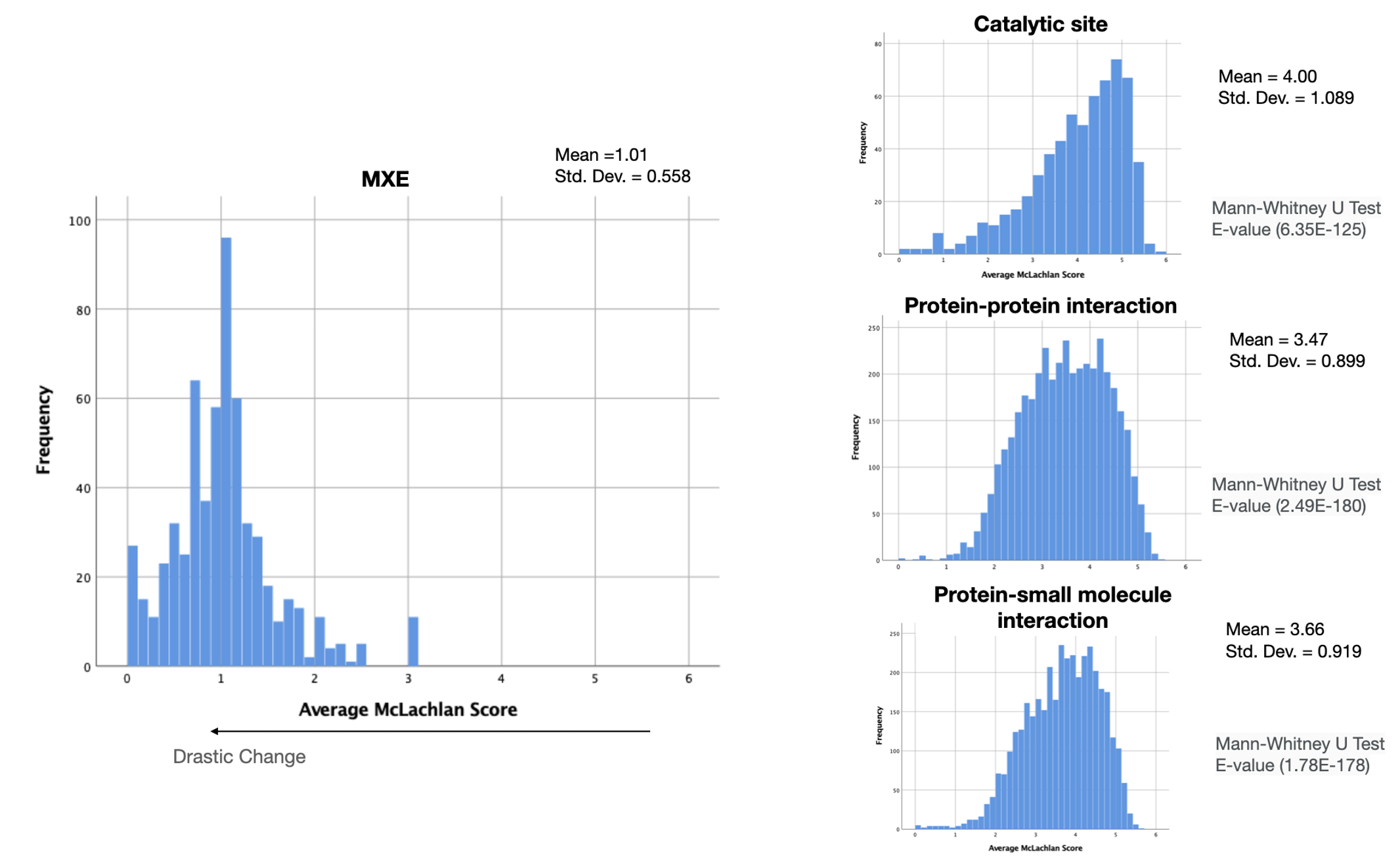

Supplement: S8 Fig — For each FunFam, with high DOPS, and a known structure we took all known functional sites (CSA (catalytic residues), PPI (protein-protein interactions), PSI (protein-small molecule Interactions)) and identified other residues within 4Å from the functional site. We then determined the residue usage and calculated the chemical changes using McLachlan score. We used Mann-Whitney U test to compute the statistical significance. (TIF) [file pcbi.1008708.s008.tif]

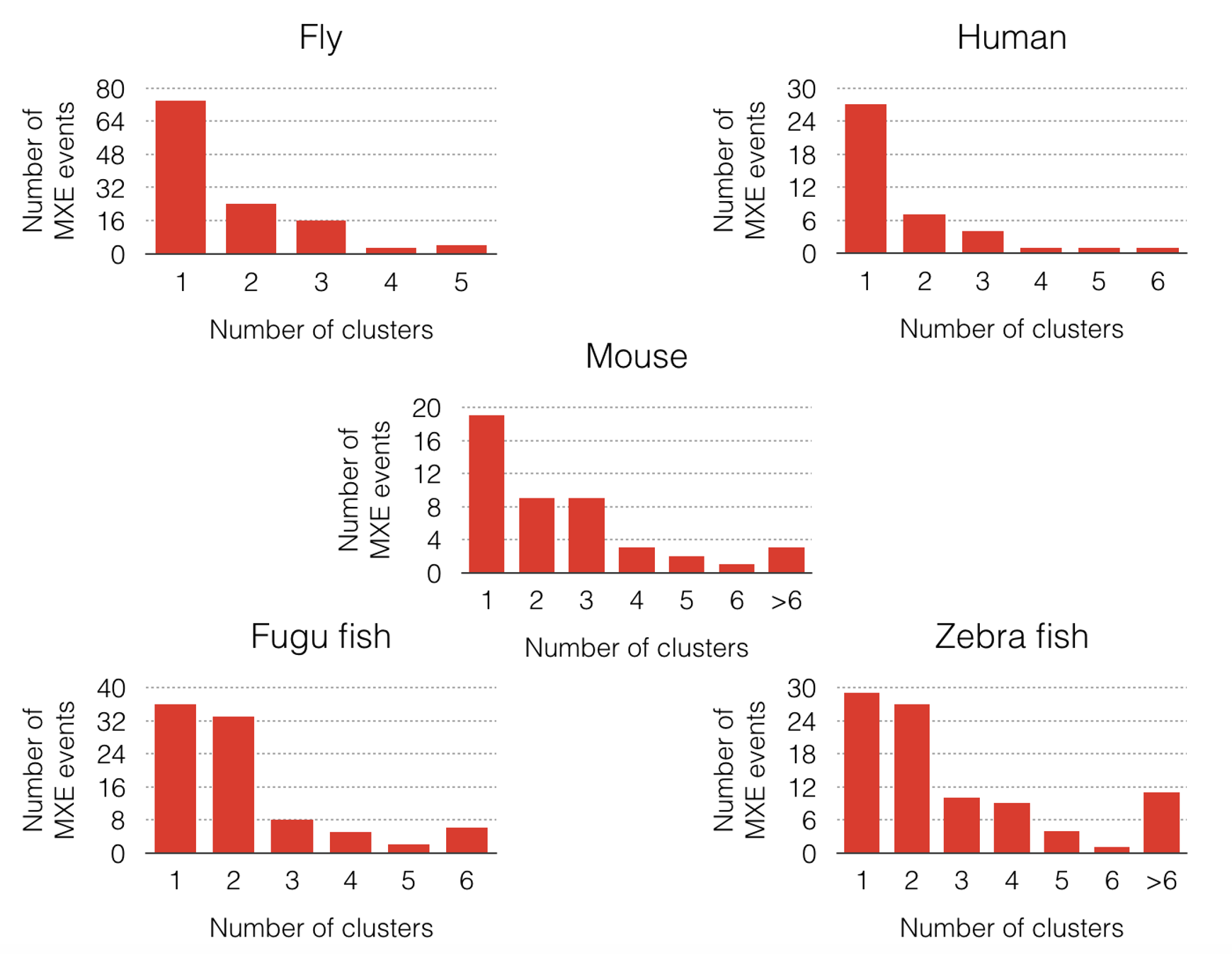

Supplement: S9 Fig — (TIF) [file pcbi.1008708.s009.tif]

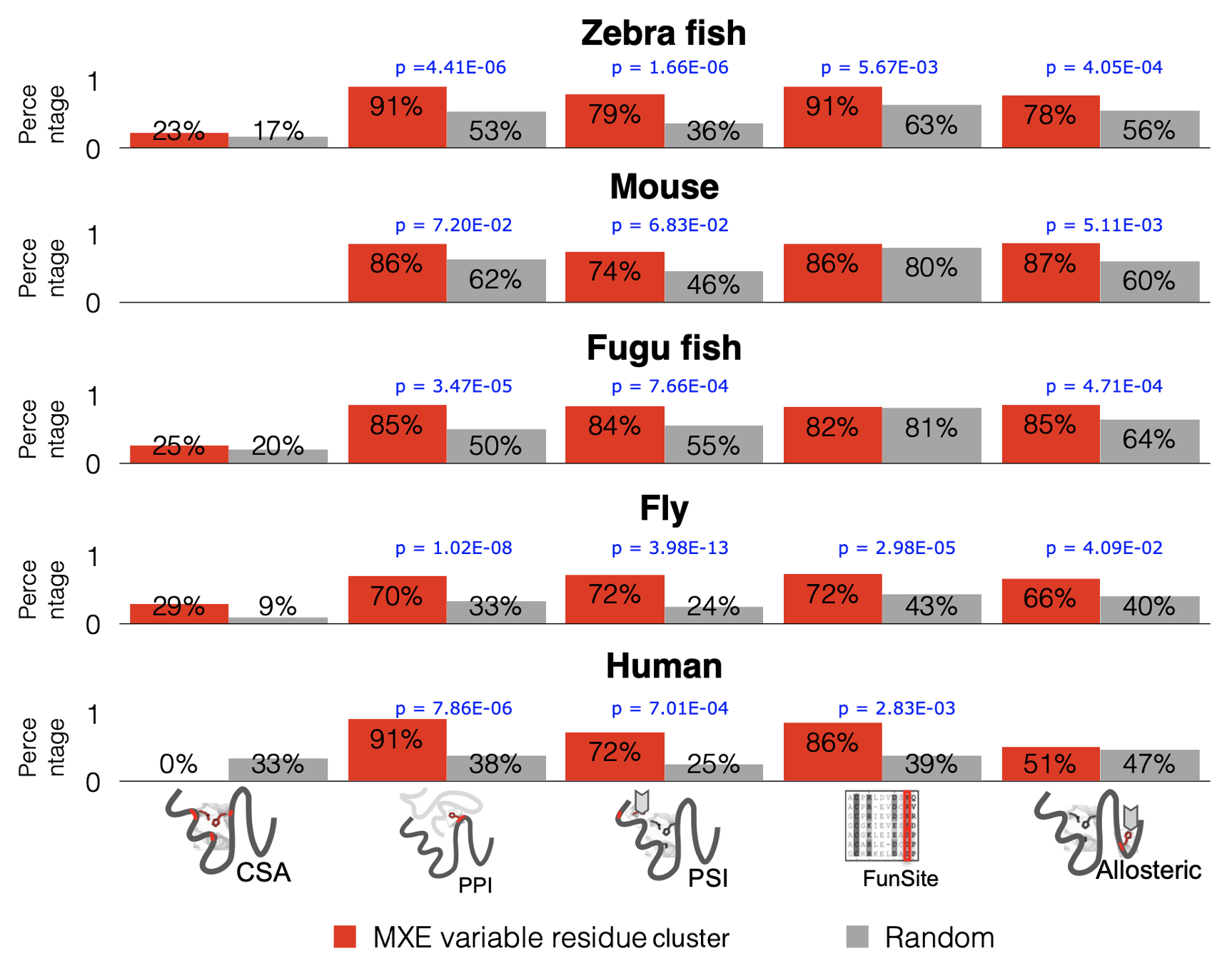

Supplement: S10 Fig — (TIF) [file pcbi.1008708.s010.tif]

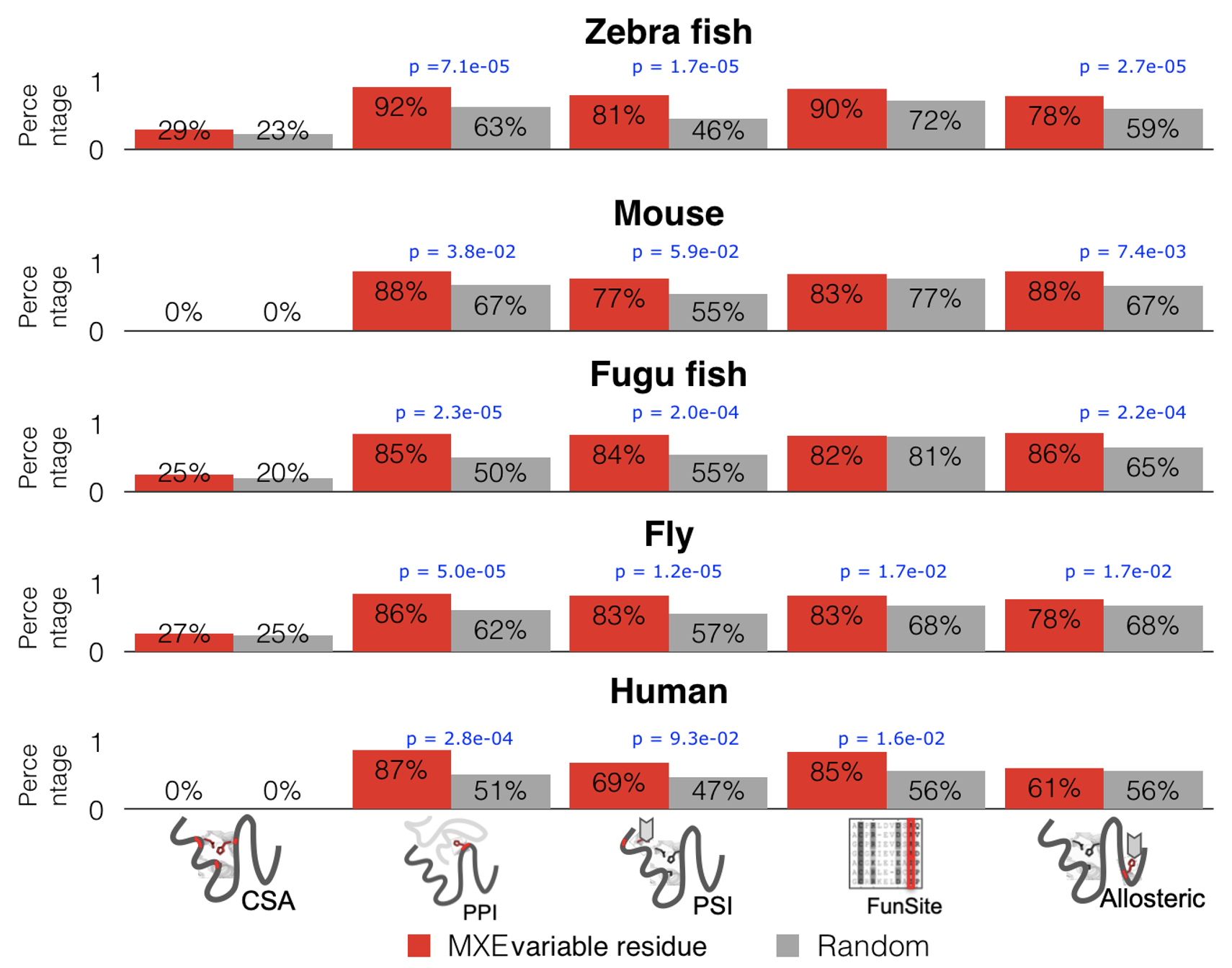

Supplement: S11 Fig — For every pair of MXE events, we determined if the variable residues within the splice regions lie close to any functional sites. We calculated the minimum distance between the atoms of the residues involved. We used a distance cut-off of 4Å to determine if residues are close. For every pair of MXE events, we created 10000 random models (based on the patterns of location of variable residues (i.e. X-X---X-X-X, X, variable residues, -, non-variable residues), and determined the percentage of highlighted residues that lie close to functional sites. We used the z-score test to compute the statistical significance. This reports a p-value for the level of significance. There is a statistically significant tendency for MXE events with variable residues to lie close to protein-protein interaction, protein-small molecule and FunSites for both fly and human datasets. There is also a tendency for variable residues to be in the vicinity of allosteric sites for the fly dataset. We had less than 10 events annotated with catalytic sites but these did not usually lie close to a MXE region using a 4Å distance cut-off. (TIF) [file pcbi.1008708.s011.tif]

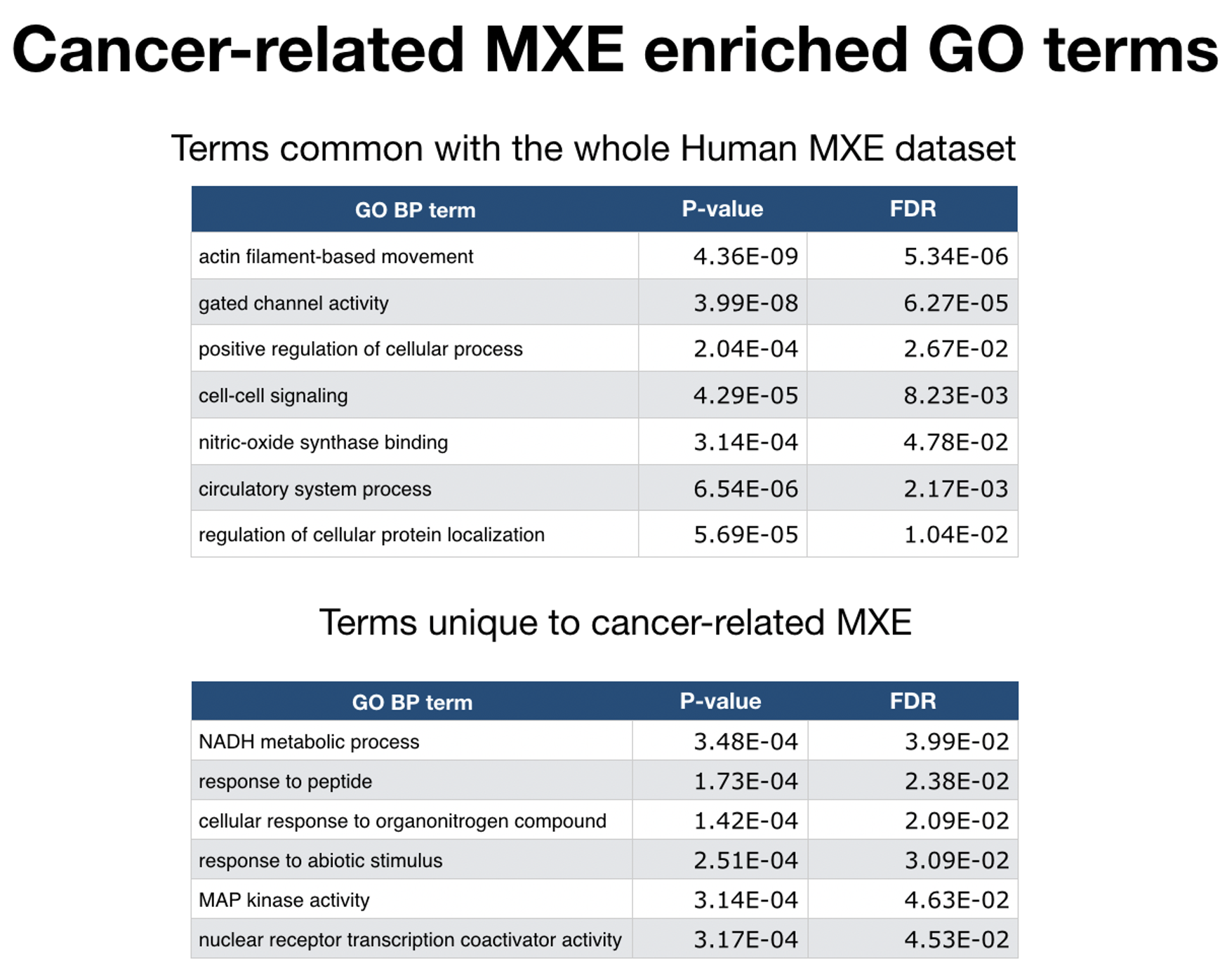

Supplement: S12 Fig — We used organism whole genome as background. (TIF) [file pcbi.1008708.s012.tif]

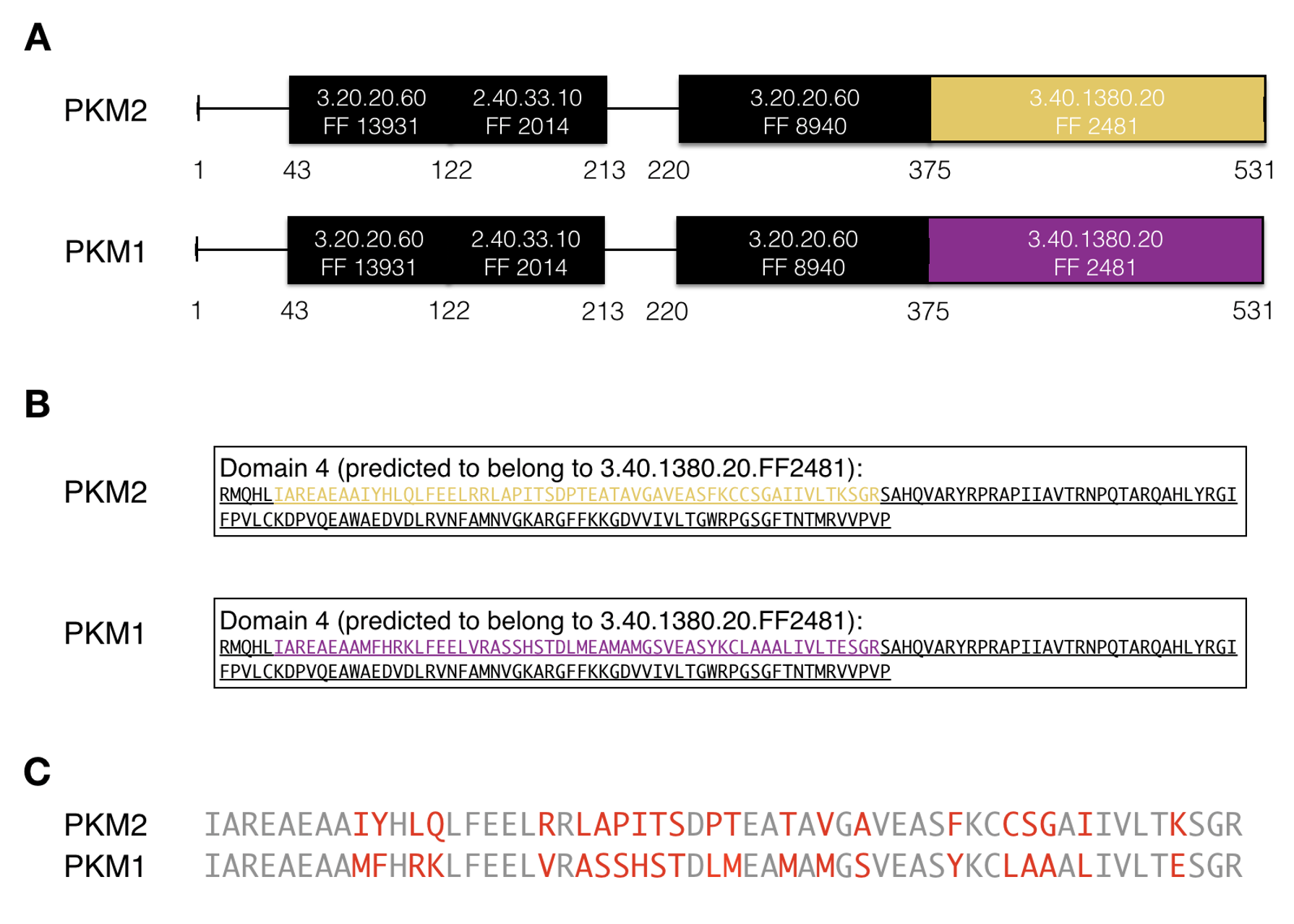

Supplement: S13 Fig — (A) Exon structures of PKM isoforms. (B) Sequences from domain 4 for the 2 isoforms. The splice regions are coloured purple or yellow. (C)The sequence alignment of the splice region. Variable residues are coloured as red. (TIF) [file pcbi.1008708.s013.tif]

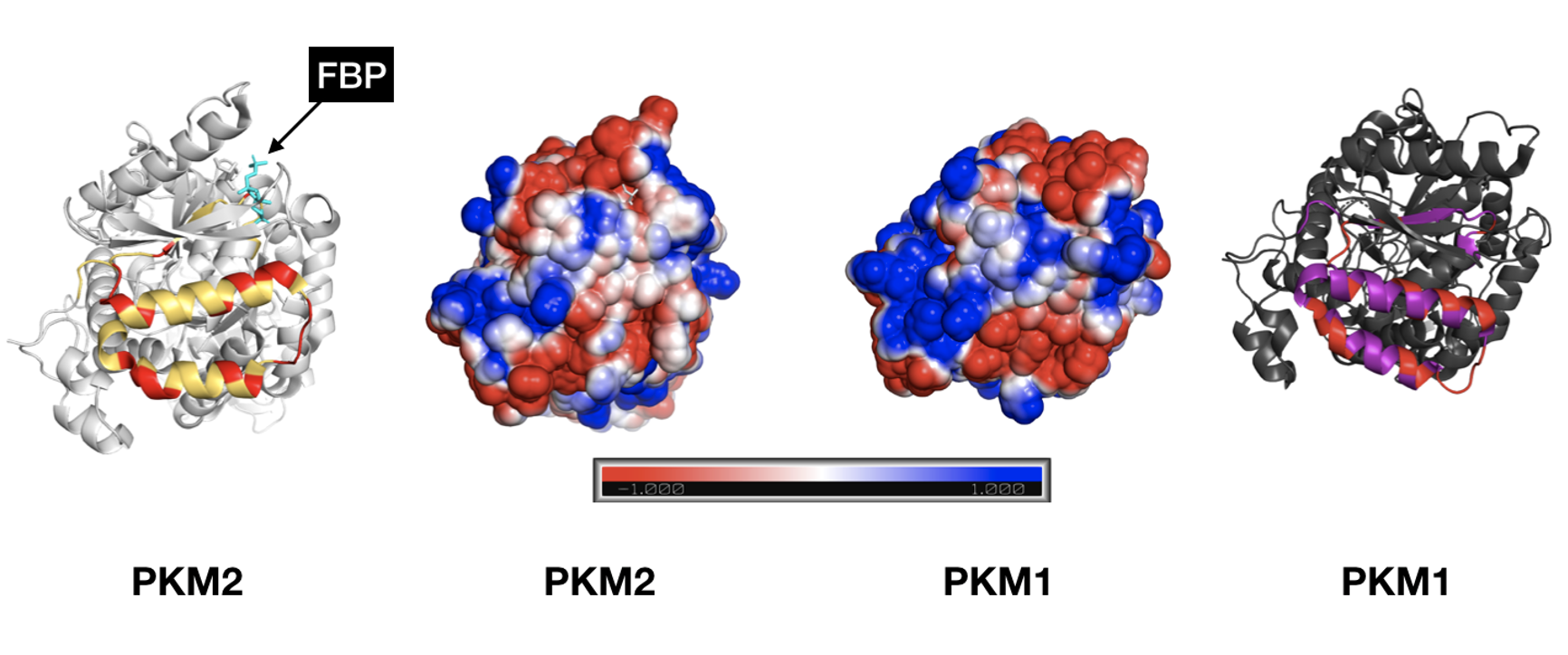

Supplement: S14 Fig — The FBP molecule is coloured in cyan. (TIF) [file pcbi.1008708.s014.tif]

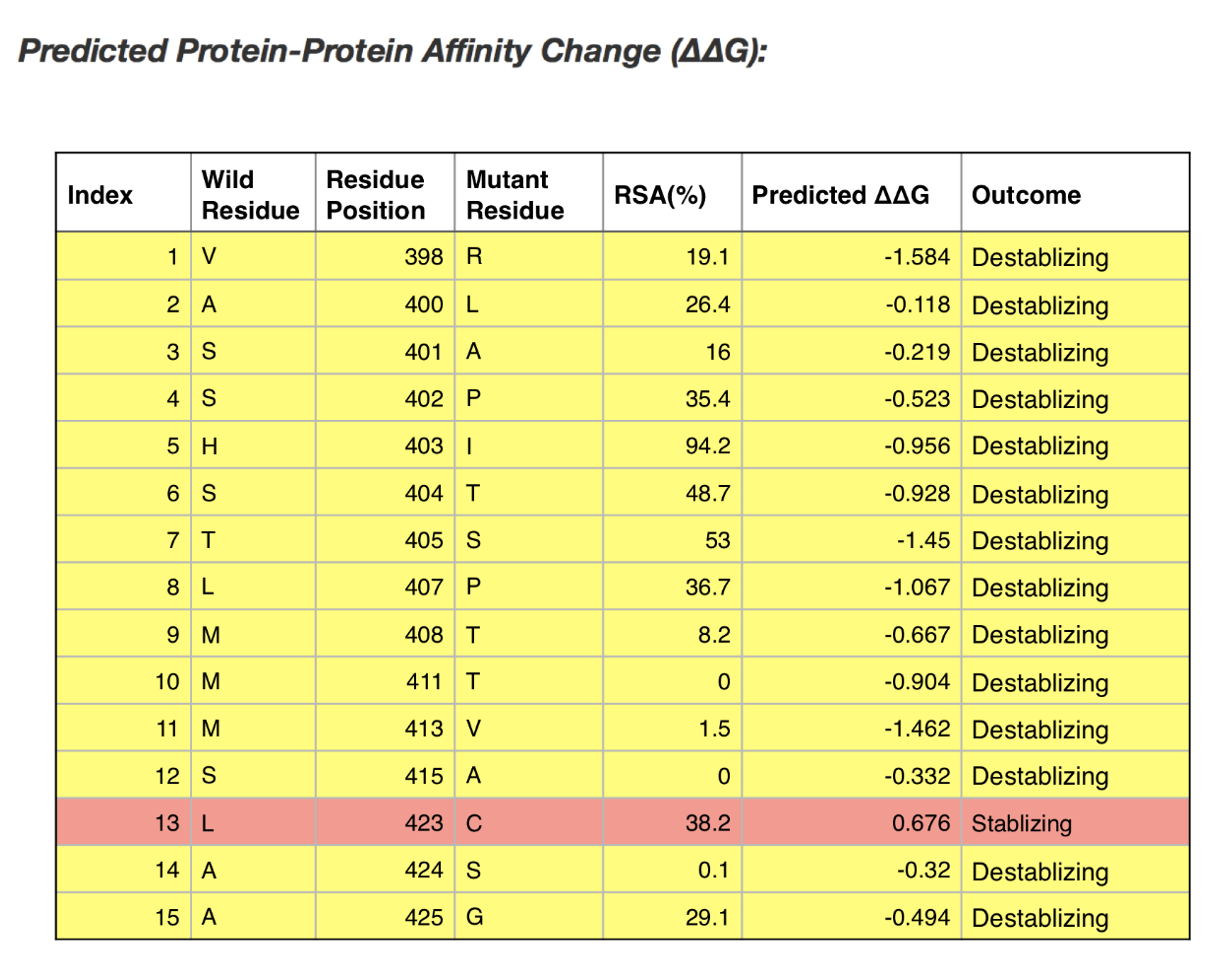

Supplement: S15 Fig — (TIF) [file pcbi.1008708.s015.tif]

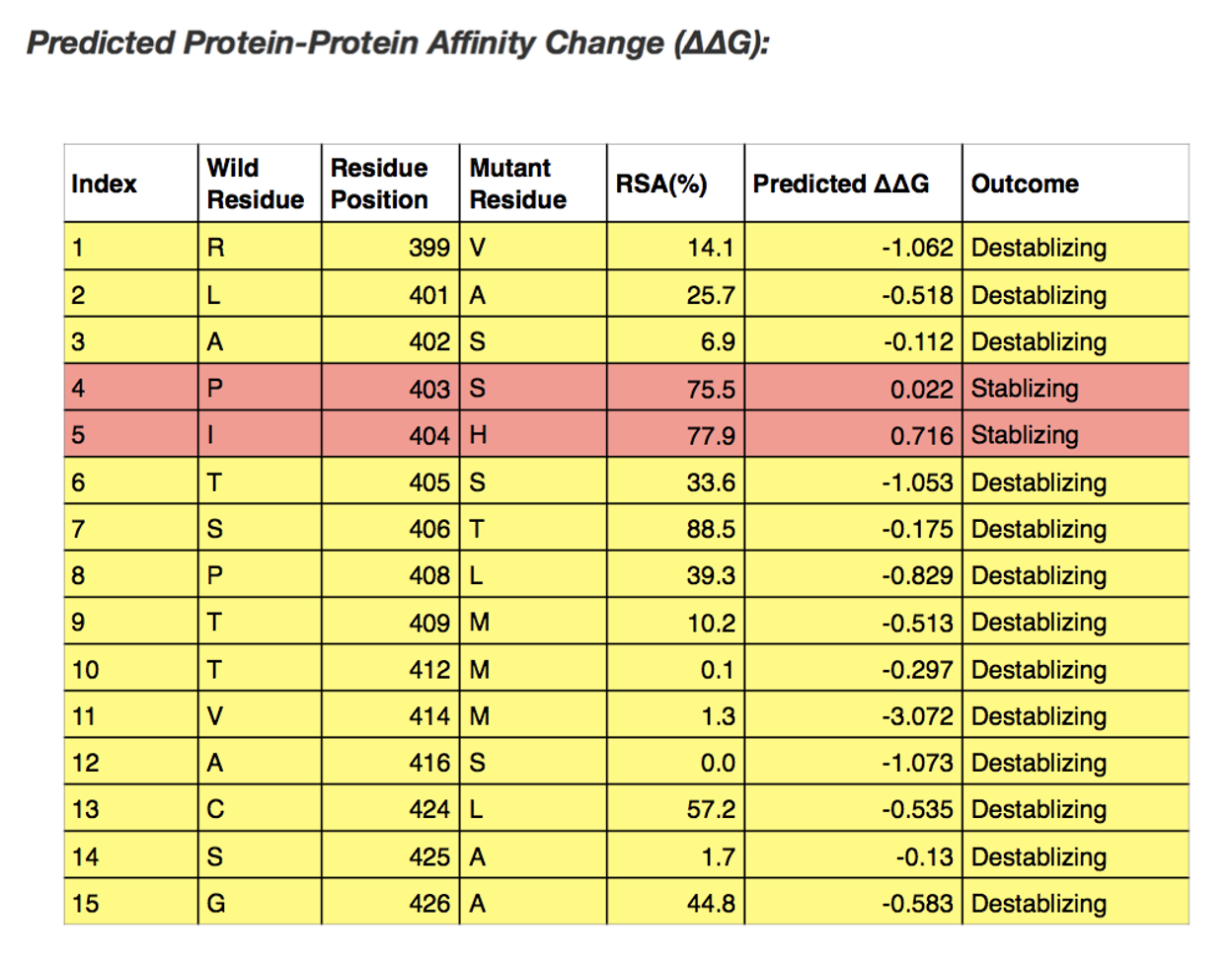

Supplement: S16 Fig — (TIF) [file pcbi.1008708.s016.tif]

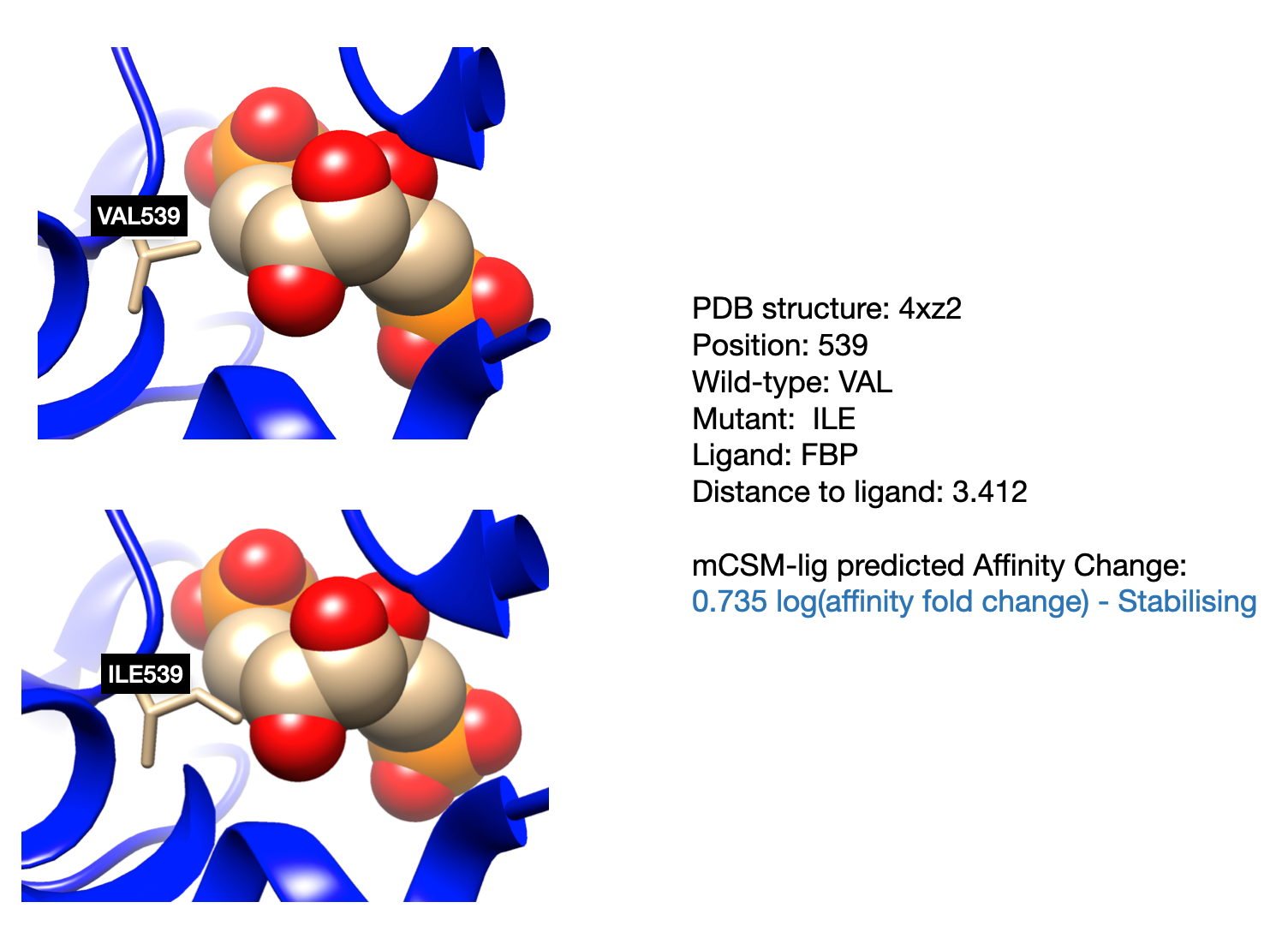

Supplement: S17 Fig — The analysis was done using PFKP structure (PDB id 4XZ2). (TIF) [file pcbi.1008708.s017.tif]

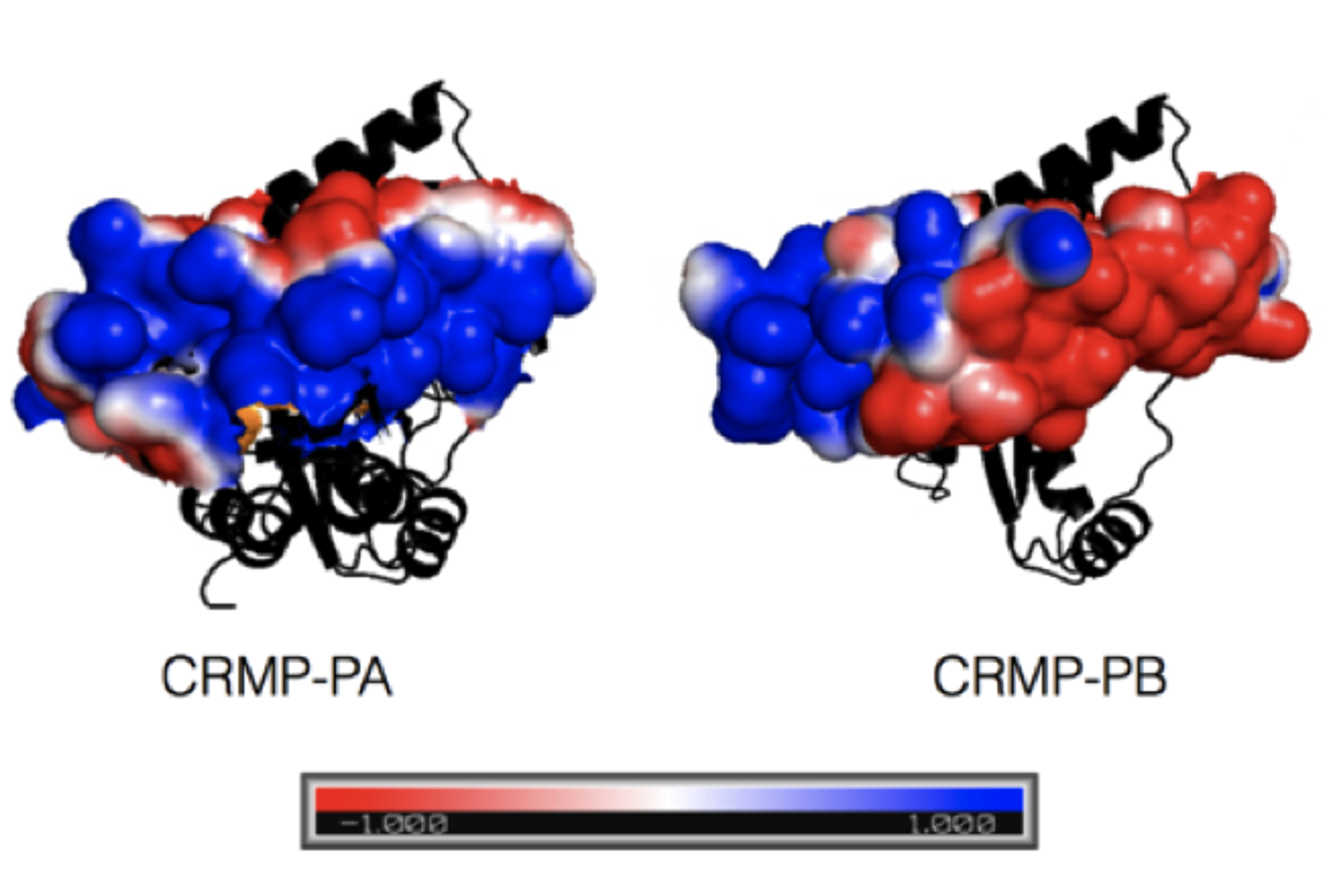

Supplement: S18 Fig — Positively charged residues are coloured as blue, negatively charged residues are coloured as red. (TIF) [file pcbi.1008708.s018.tif]

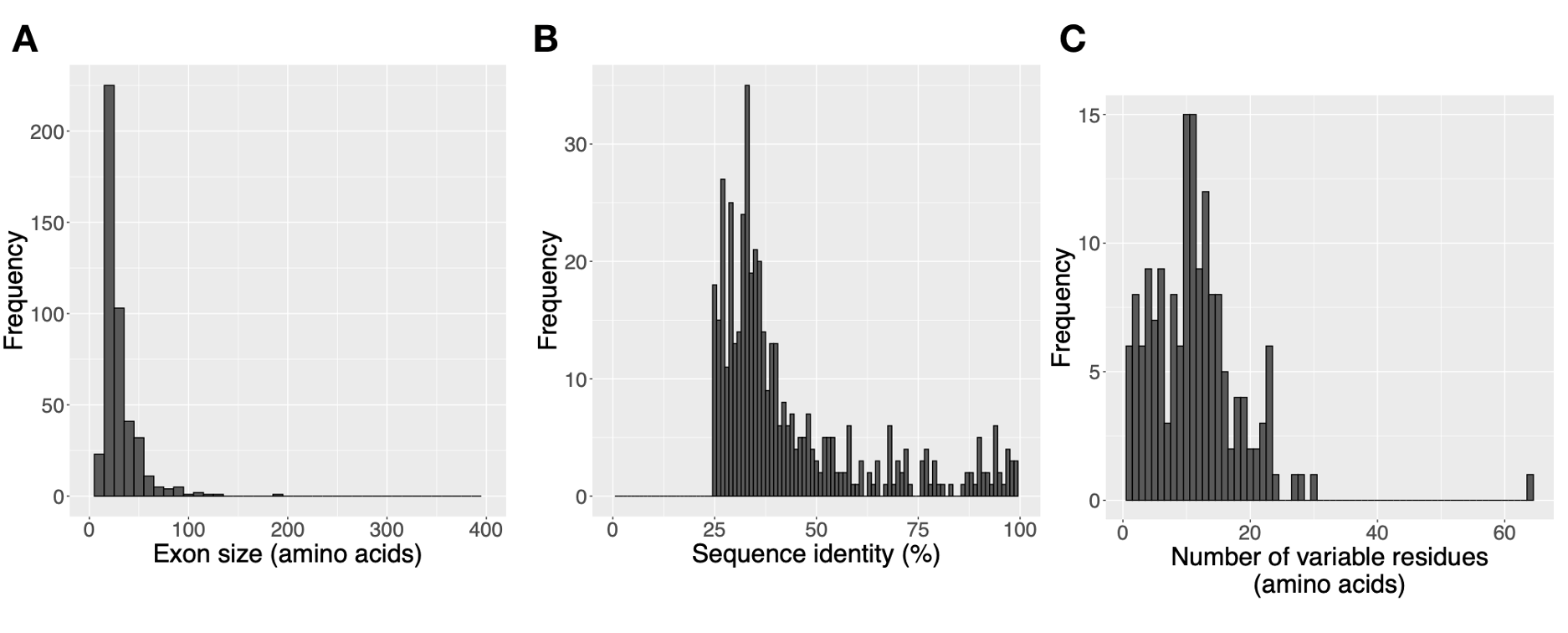

Supplement: S19 Fig — (A) Distributions of MXE sizes. (B) Sequence identity between MXE pairs. (C) Number of variable residues between MXE pairs. (TIF) [file pcbi.1008708.s019.tif]

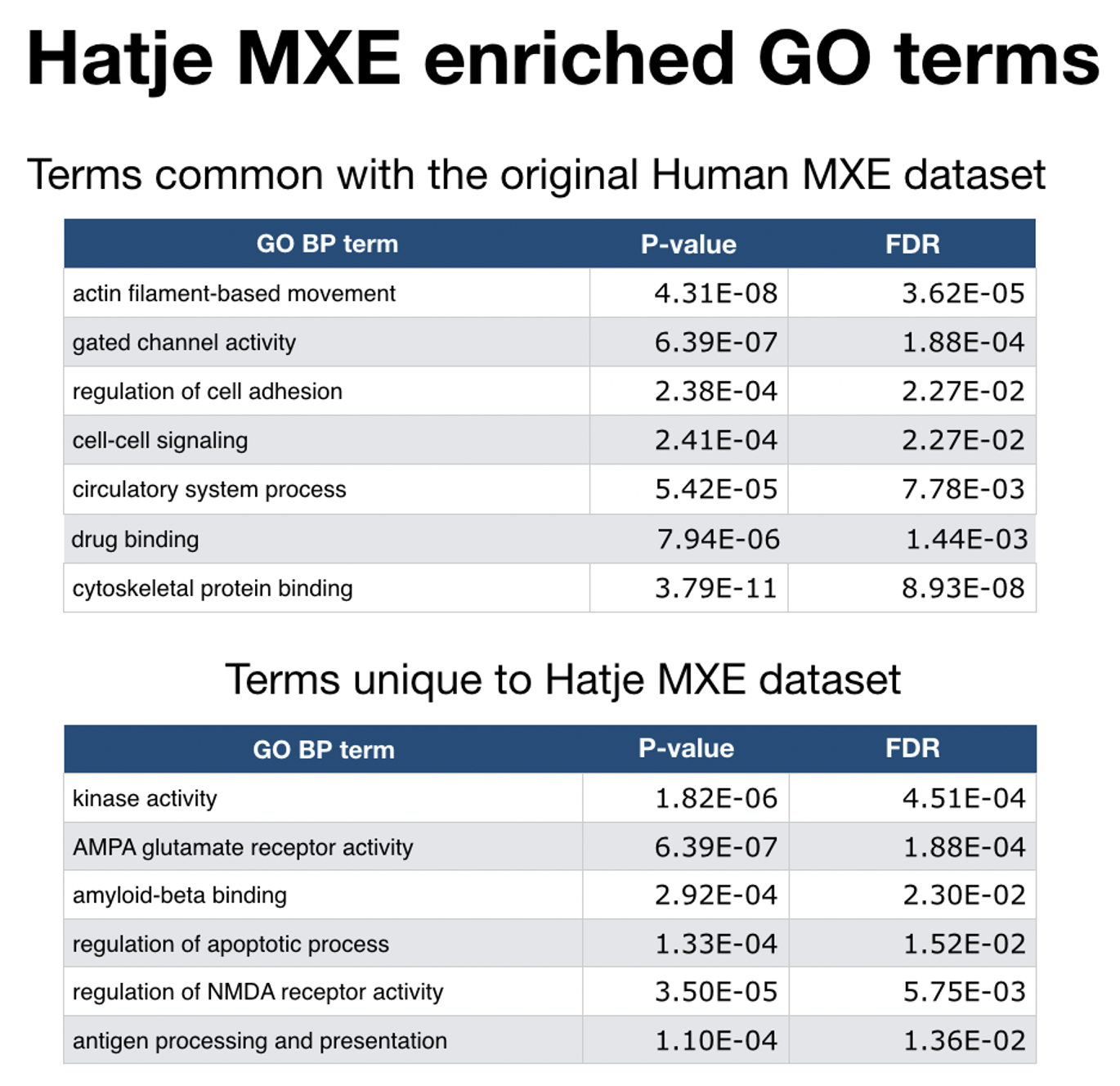

Supplement: S20 Fig — We used organism whole genome as background. (TIF) [file pcbi.1008708.s020.tif]

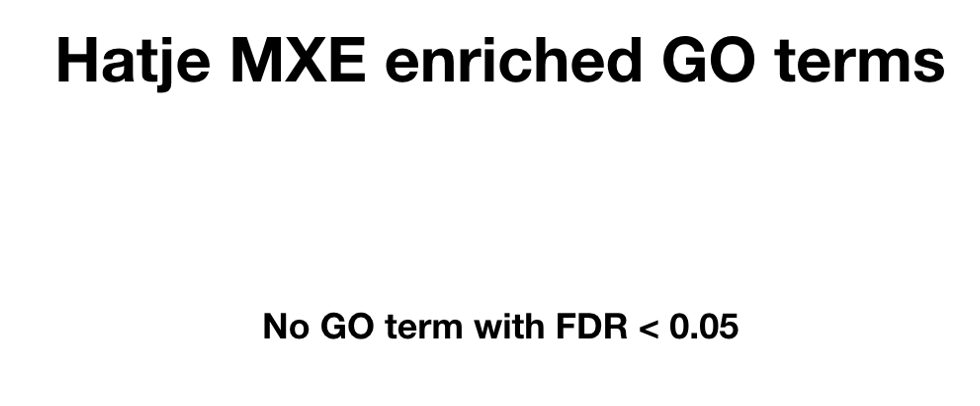

Supplement: S21 Fig — For our background dataset, we only used genes that have no paralogs. Removing the paralogs was to counter potential effects from MXE events being retained after a gene duplication, which could lead to overestimates of functional coherence of the gene set. (TIF) [file pcbi.1008708.s021.tif]

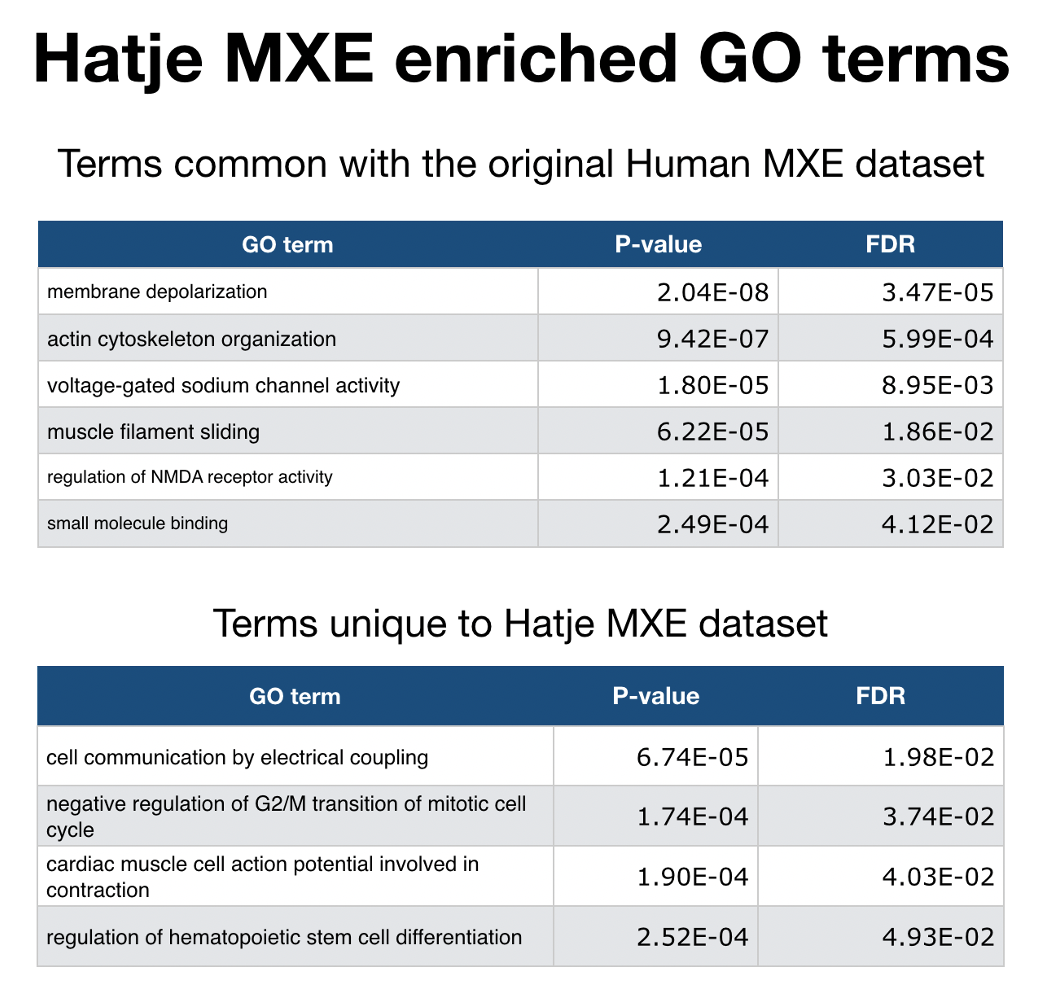

Supplement: S22 Fig — We used only multi-exon genes as background. Removing single exon genes was to counter potential effects of any functional bias, since by definition our MXE genes require more than one exon. (TIF) [file pcbi.1008708.s022.tif]

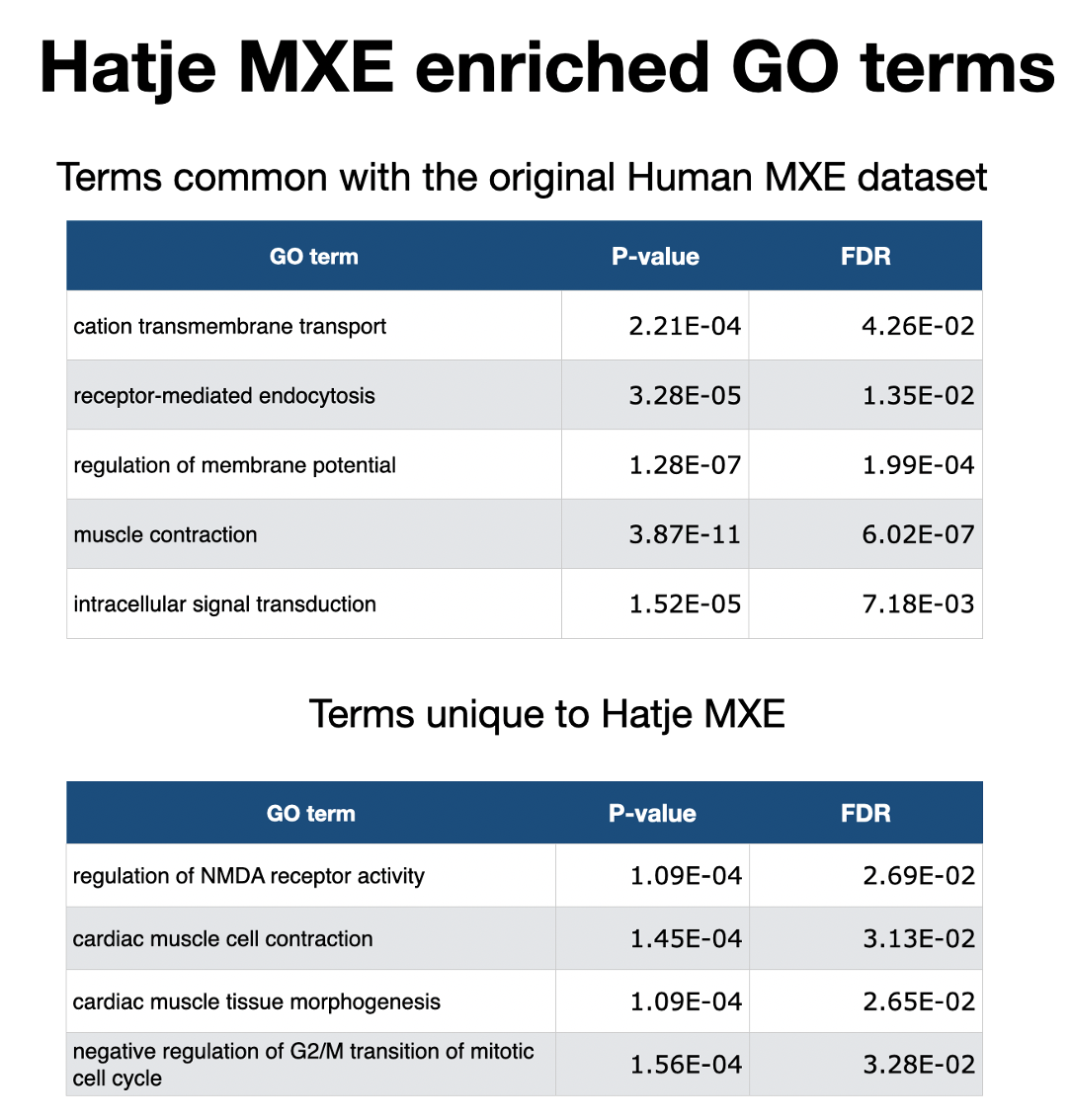

Supplement: S23 Fig — We used only multi-protein-isoform genes as background. Removing single protein-isoform exon genes was to counter potential effects of any functional bias, since by definition our MXE genes require more than one exon. (TIF) [file pcbi.1008708.s023.tif]

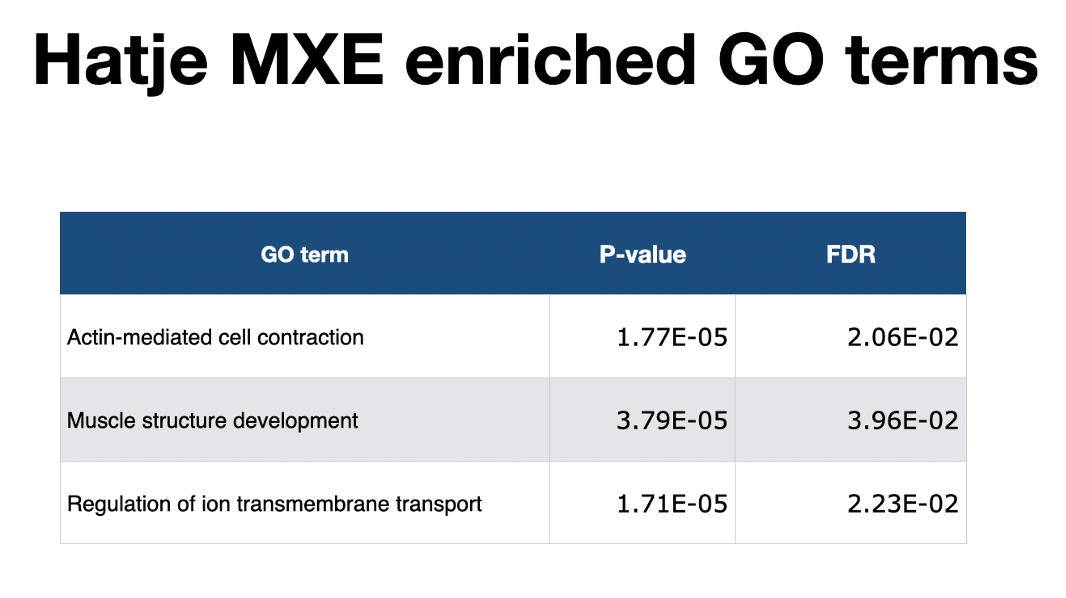

Supplement: S24 Fig — We used only MXE or CE genes as background to counter potential effects of any functional bias. (TIF) [file pcbi.1008708.s024.tif]

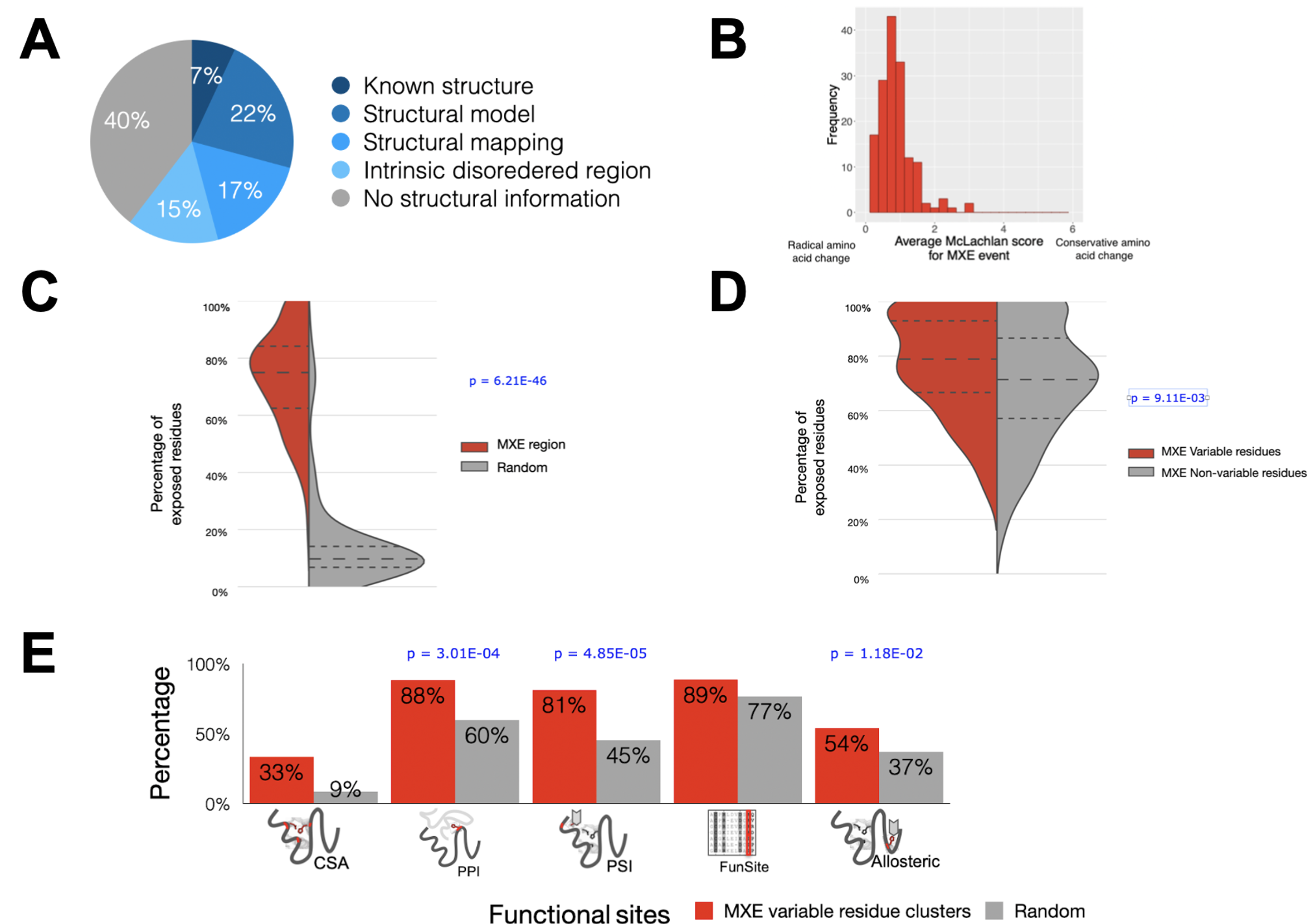

Supplement: S25 Fig — (A) Proportion of splice isoforms with structural information. (B) Distribution of McLachlan scores for the variable MXE residues. (C) The surface exposure of the MXE events compared to random expectation. (D)The surface exposure of the MXE variable residues compared to the MXE event as a whole. (E) Proximity of MXE variable residue clusters to amino acids assigned to different functional classes (indicated by Icons) CSA = catalytic residues from the Catalytic Site Atlas, PPI = protein interaction sites and PSI = protein-small molecule interactions). (TIF) [file pcbi.1008708.s025.tif]

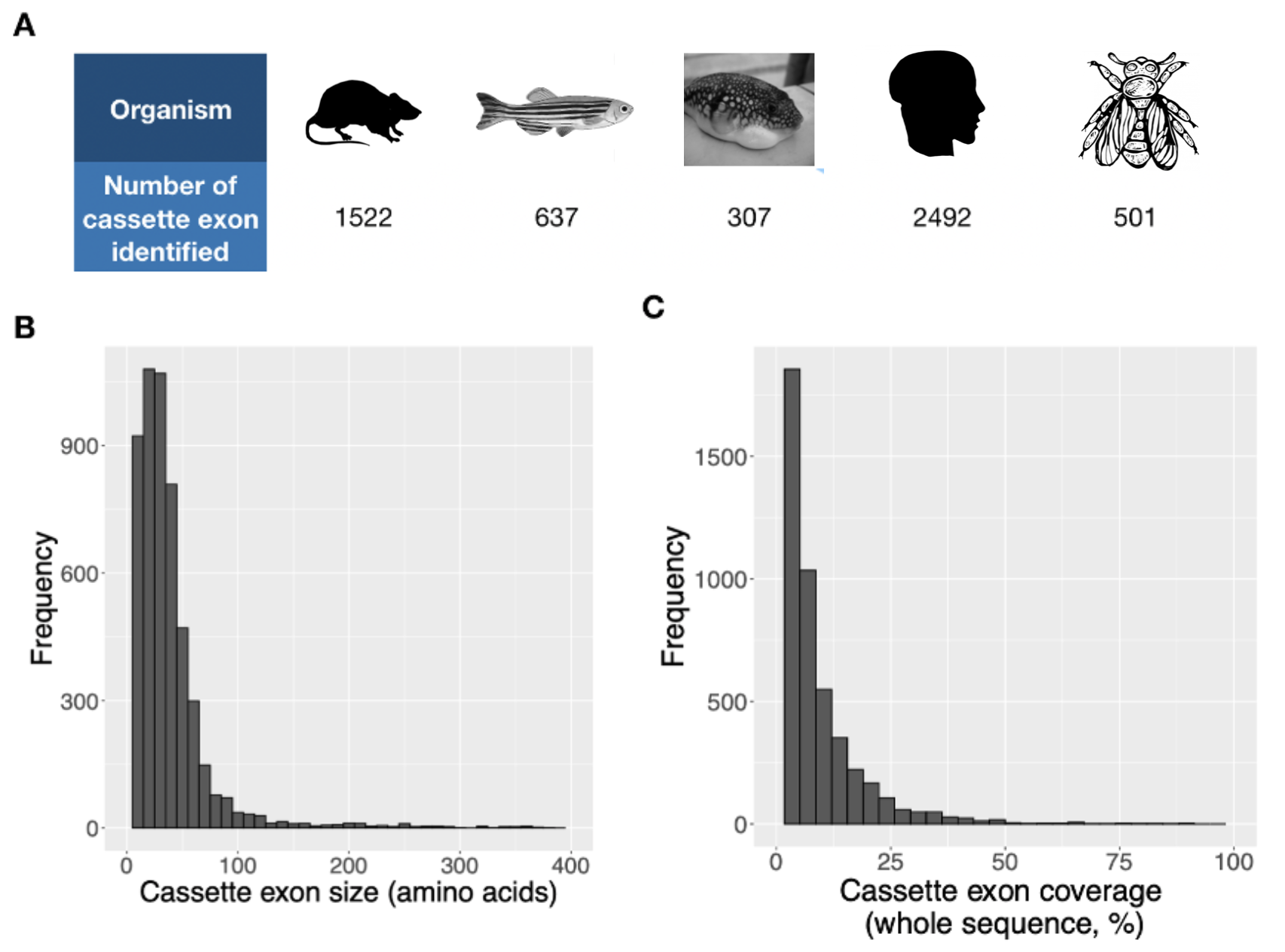

Supplement: S26 Fig — (A) Number of cassette exons identified (B) Distribution of cassette exon sizes. (C) Distribution of cassette exon coverage (whole sequence). (TIF) [file pcbi.1008708.s026.tif]

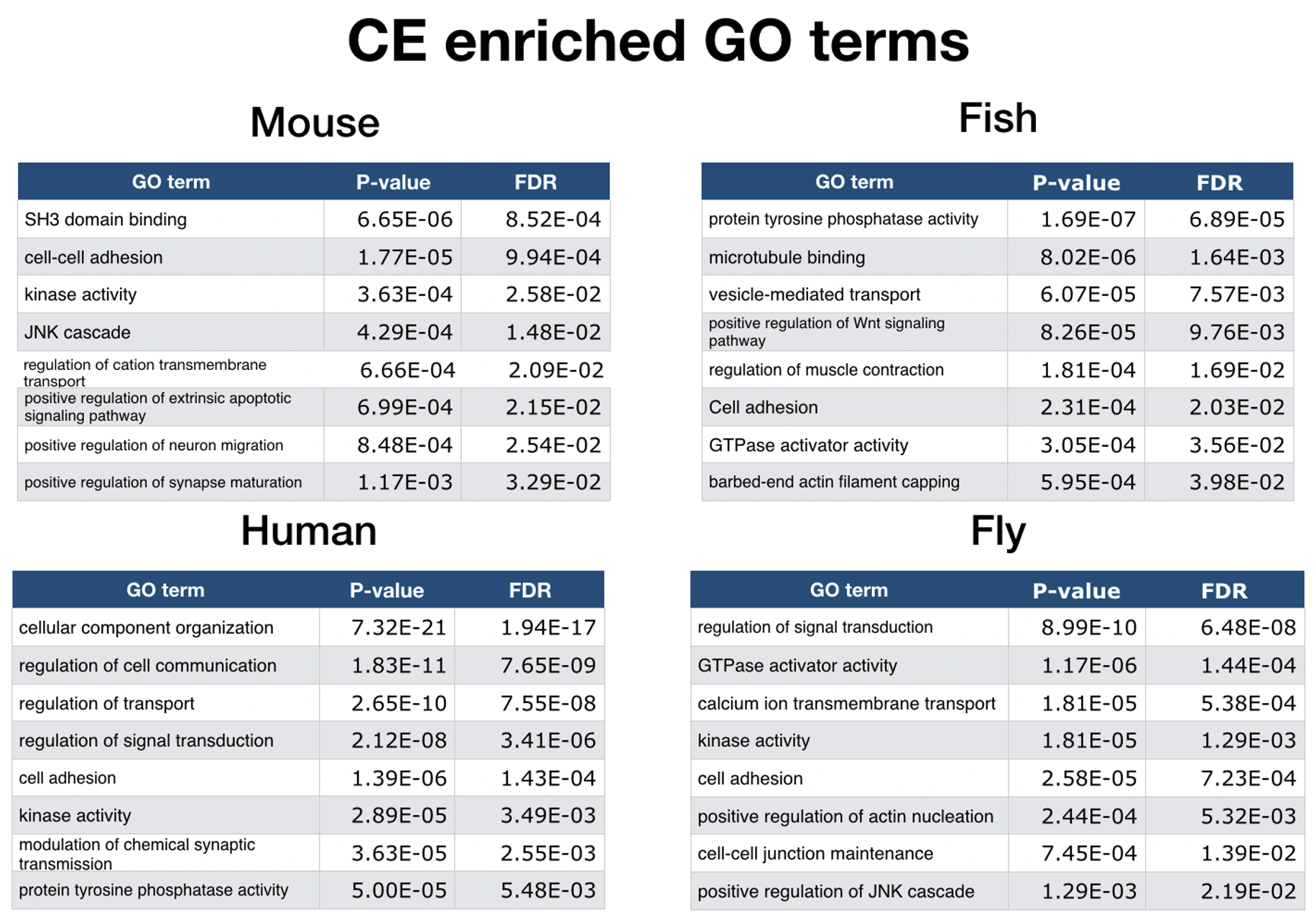

Supplement: S27 Fig — We used organism whole genome as background. (TIF) [file pcbi.1008708.s027.tif]

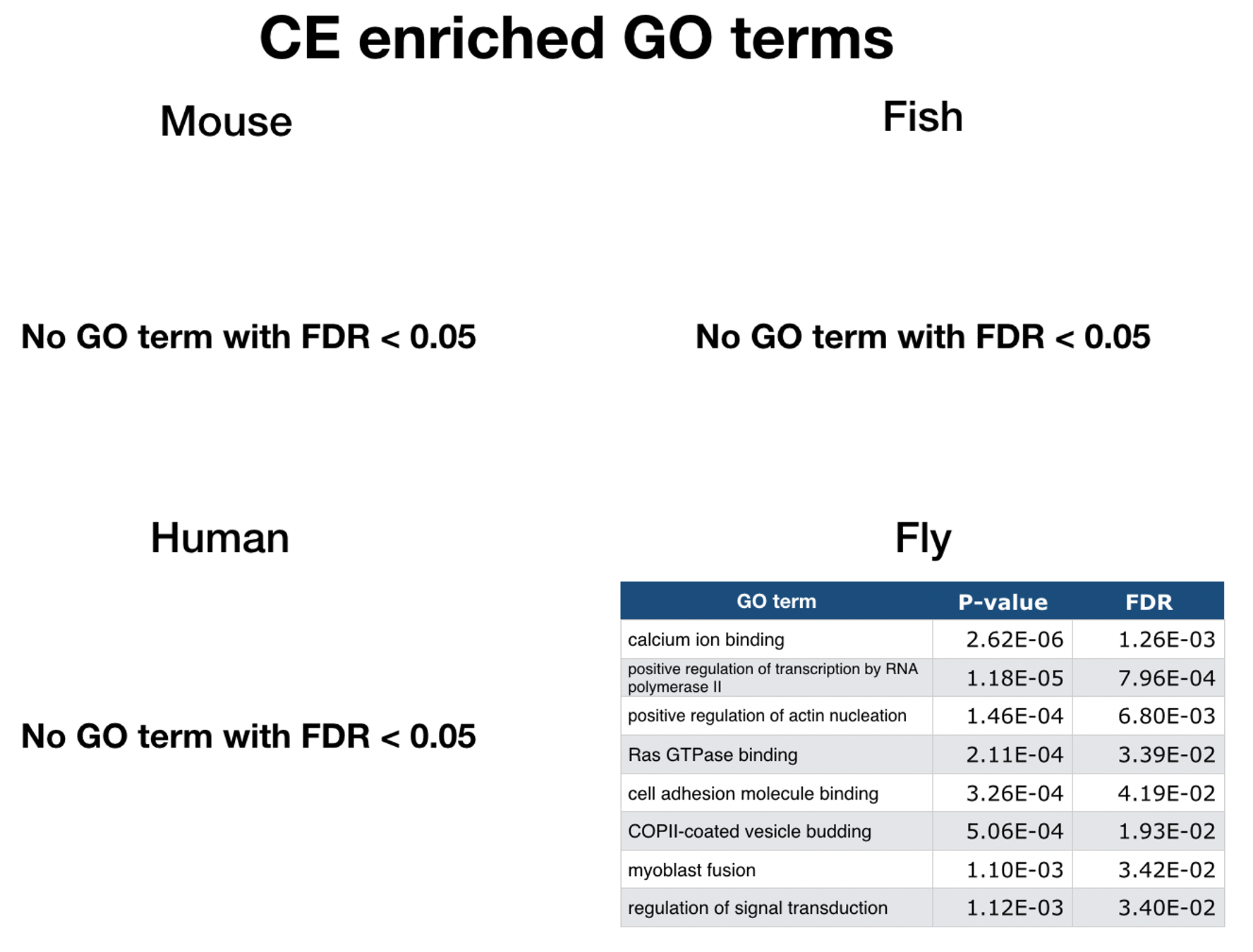

Supplement: S28 Fig — For our background dataset, we only used genes that have no paralogs. Removing the paralogs was to counter potential effects from MXE events being retained after a gene duplication, which could lead to overestimates of functional coherence of the gene set. (TIF) [file pcbi.1008708.s028.tif]

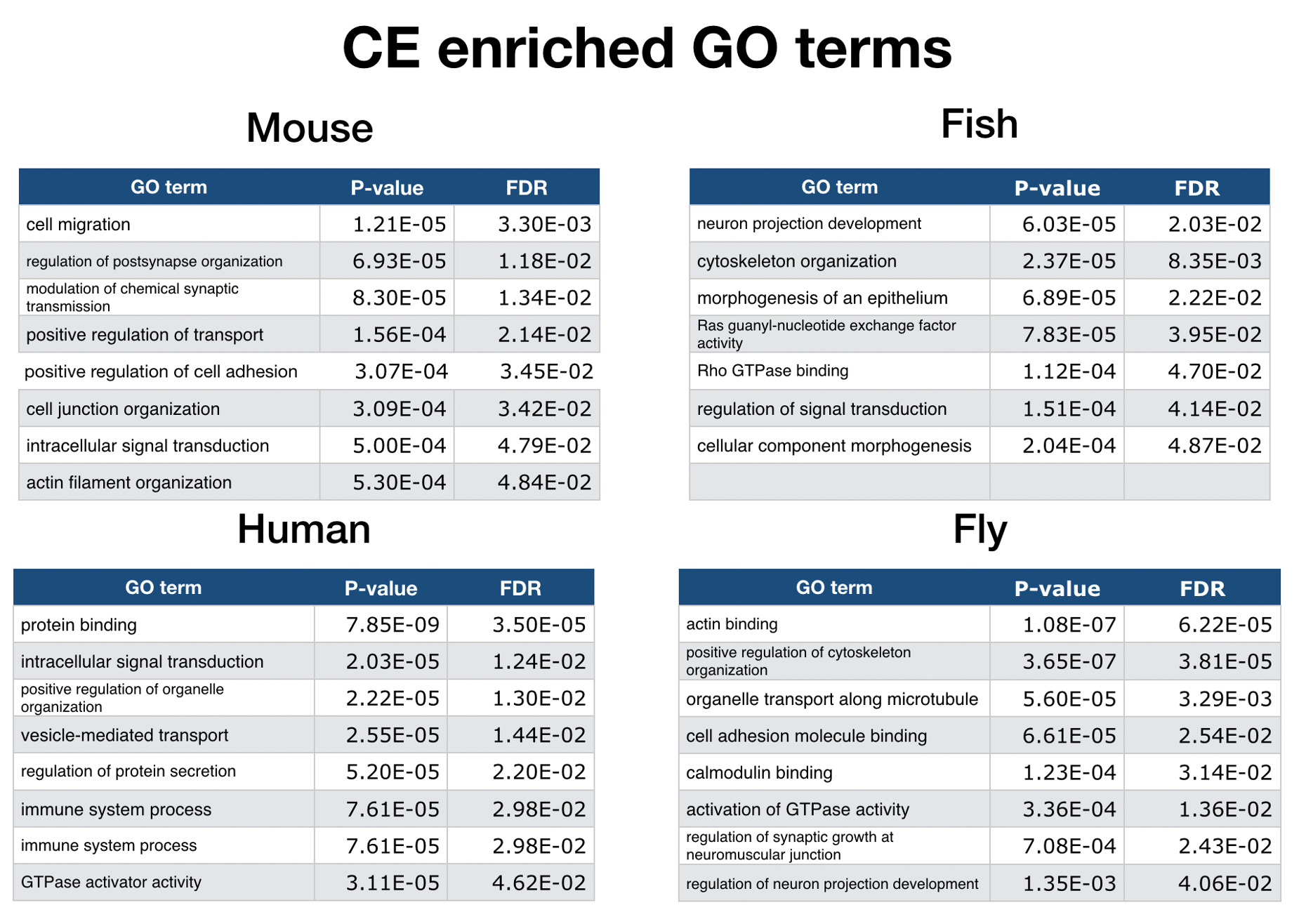

Supplement: S29 Fig — We used only multi-exon genes as background. Removing single exon genes was to counter potential effects of any functional bias, since by definition our MXE genes require more than one exon. (TIF) [file pcbi.1008708.s029.tif]

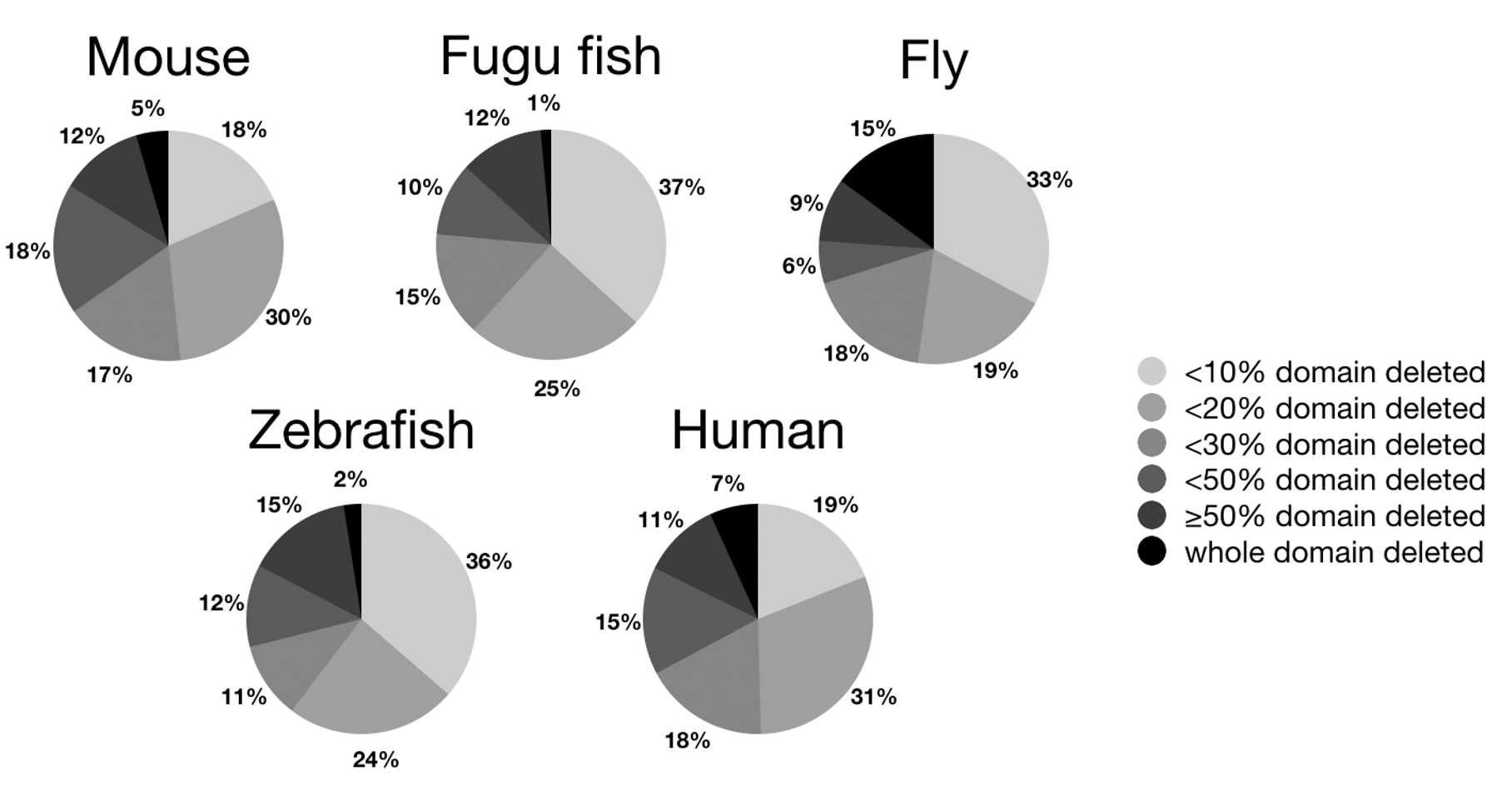

Supplement: S30 Fig — (TIF) [file pcbi.1008708.s030.tif]

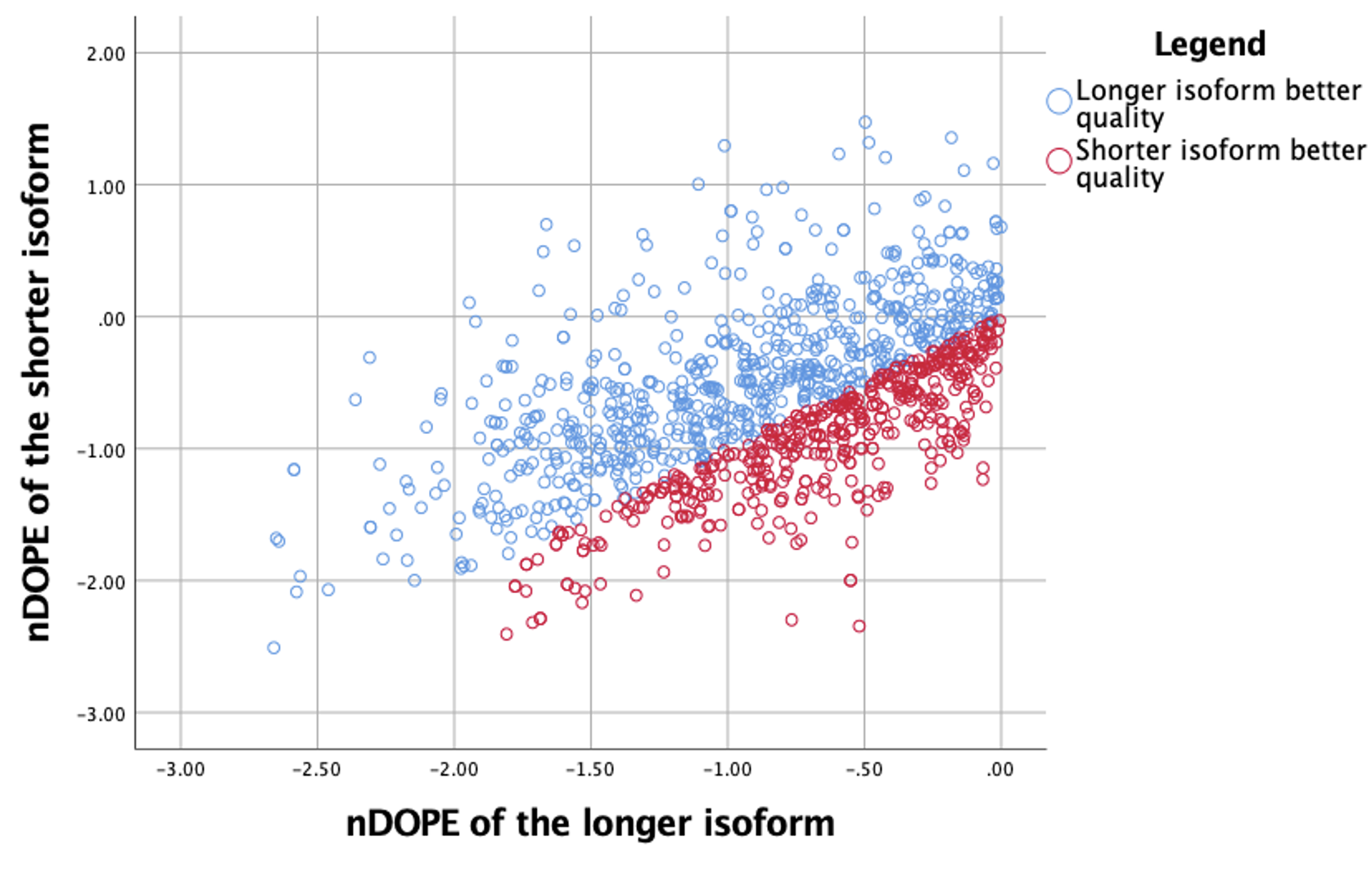

Supplement: S31 Fig — We only show cases where an acceptable quality model can be built for the longer isoform. (TIF) [file pcbi.1008708.s031.tif]

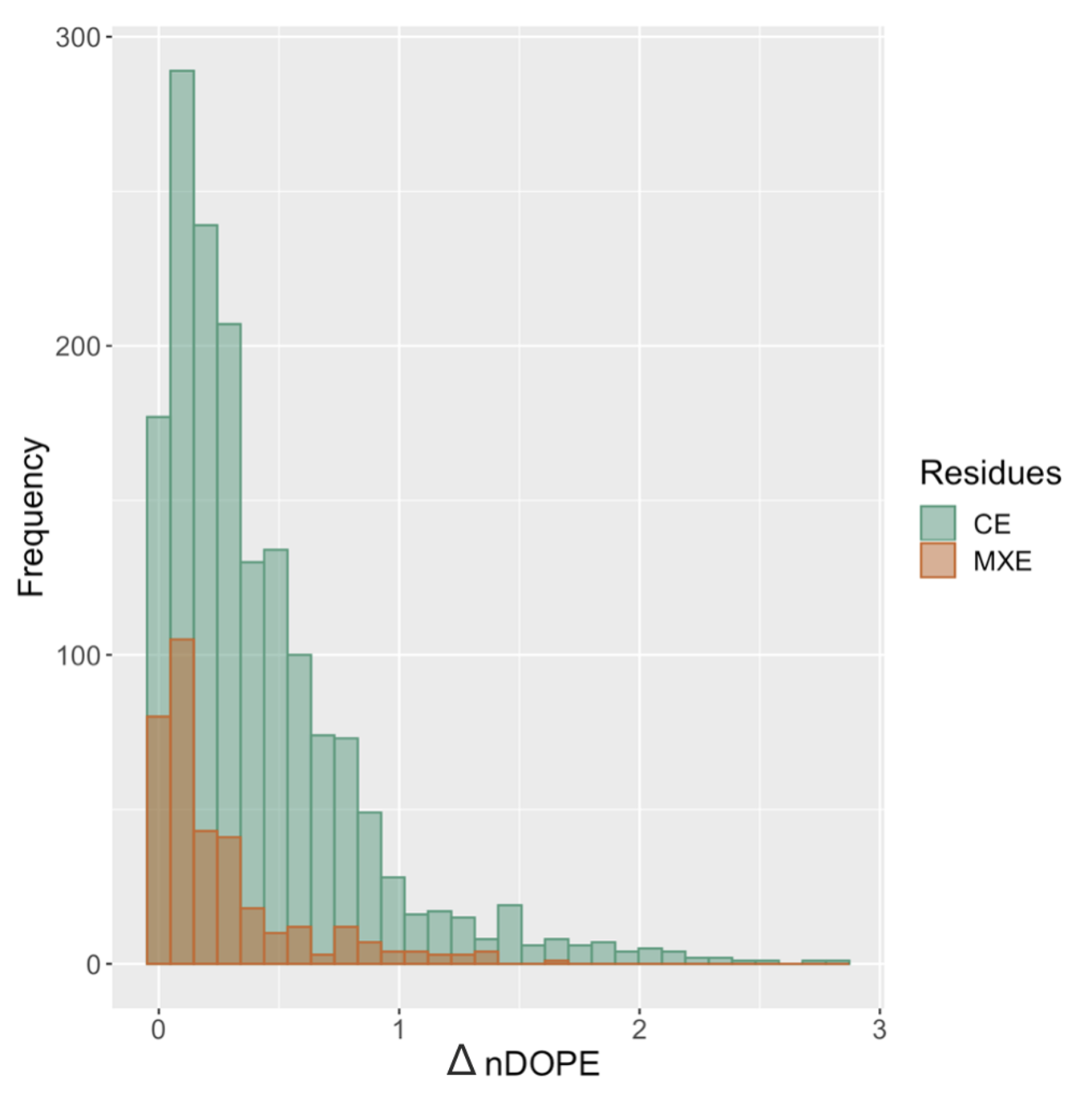

Supplement: S32 Fig — The difference in nDOPE score is given by a ΔnDOPE value (by subtracting the nDOPEs of the two isoforms and then taking the absolute value). (TIF) [file pcbi.1008708.s032.tif]

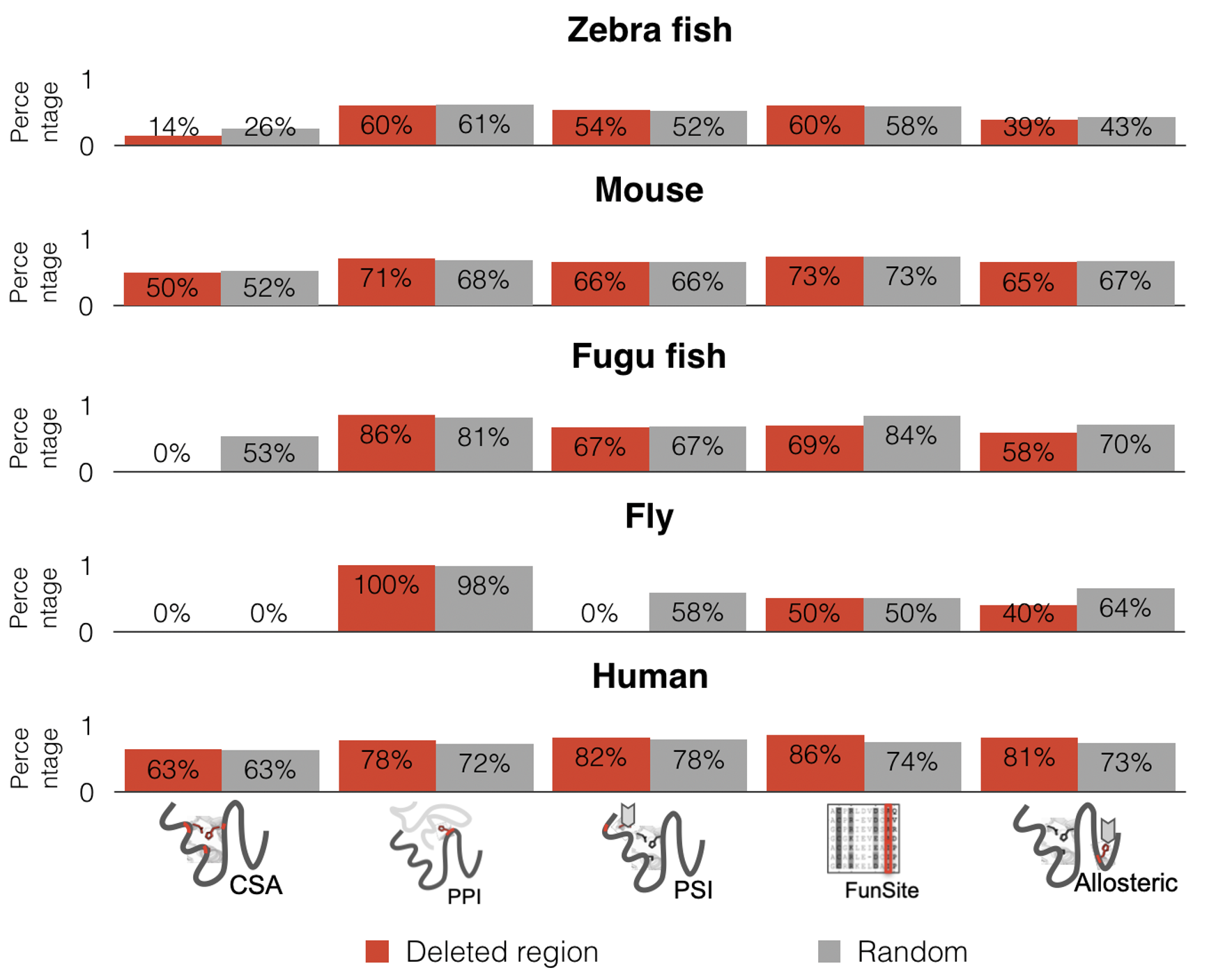

Supplement: S33 Fig — (TIF) [file pcbi.1008708.s033.tif]

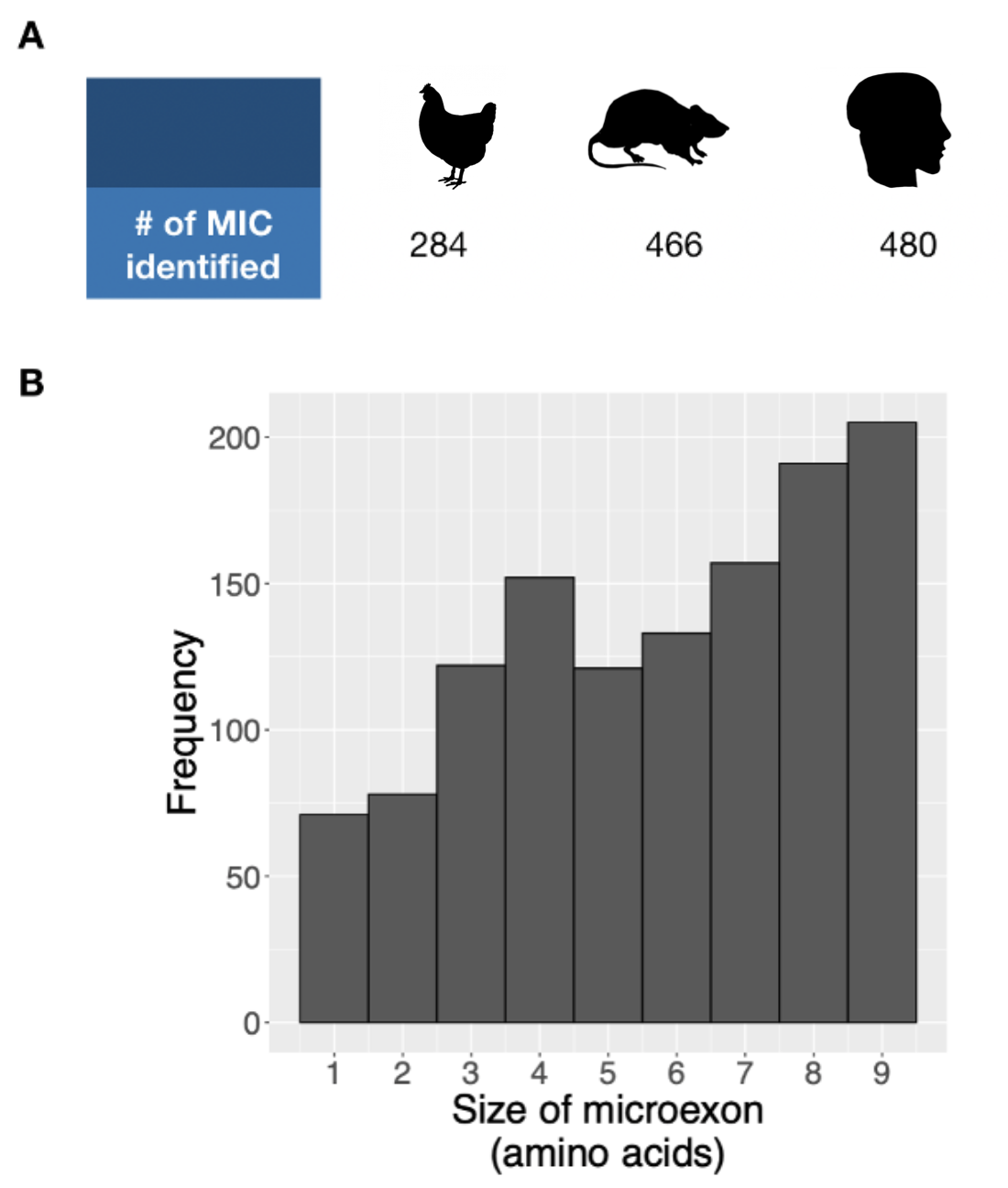

Supplement: S34 Fig — (A) Number of MIC events identified (B) Distributions of MIC size. (TIF) [file pcbi.1008708.s034.tif]

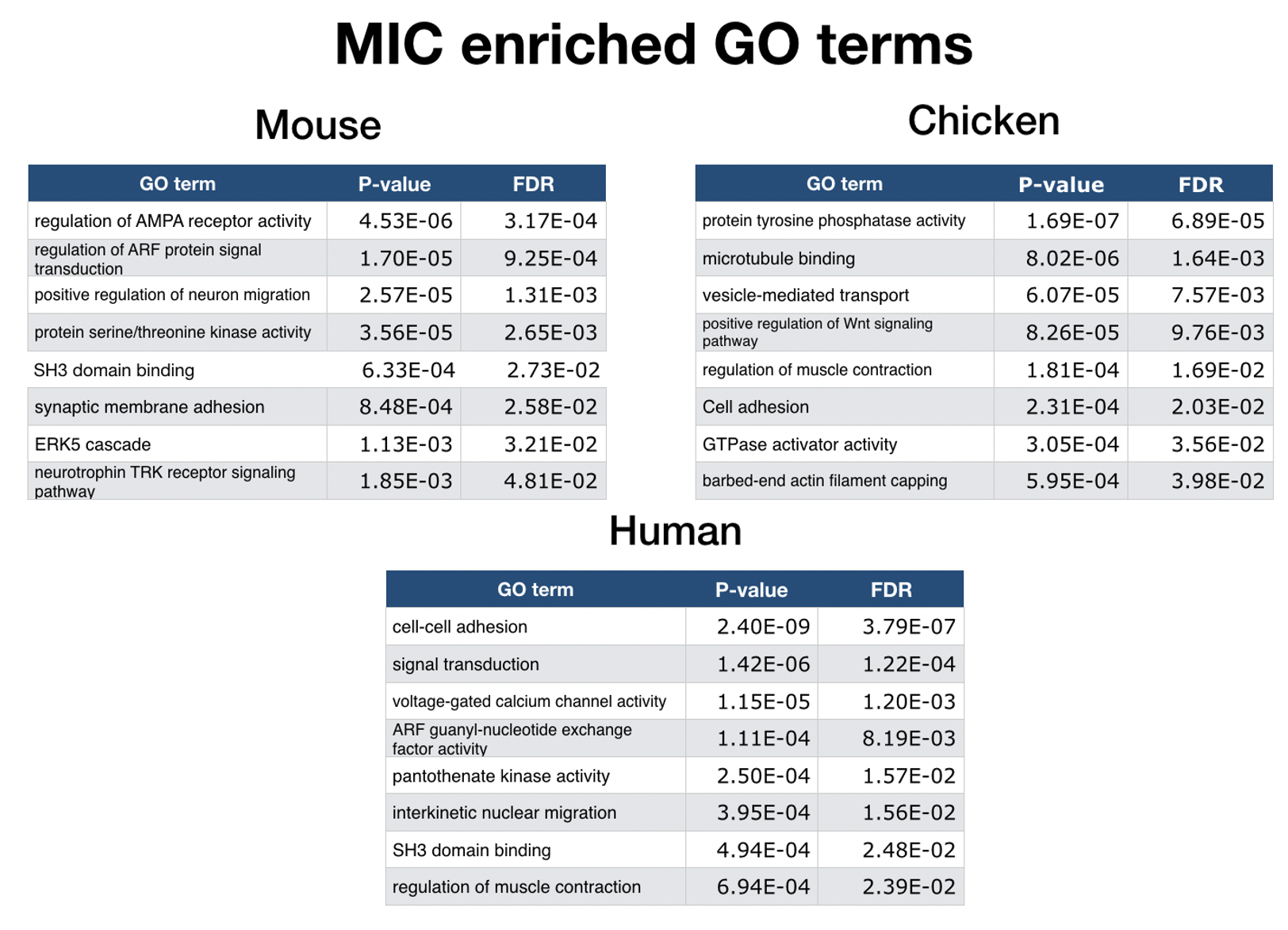

Supplement: S35 Fig — We used organism whole genome as background. (TIF) [file pcbi.1008708.s035.tif]

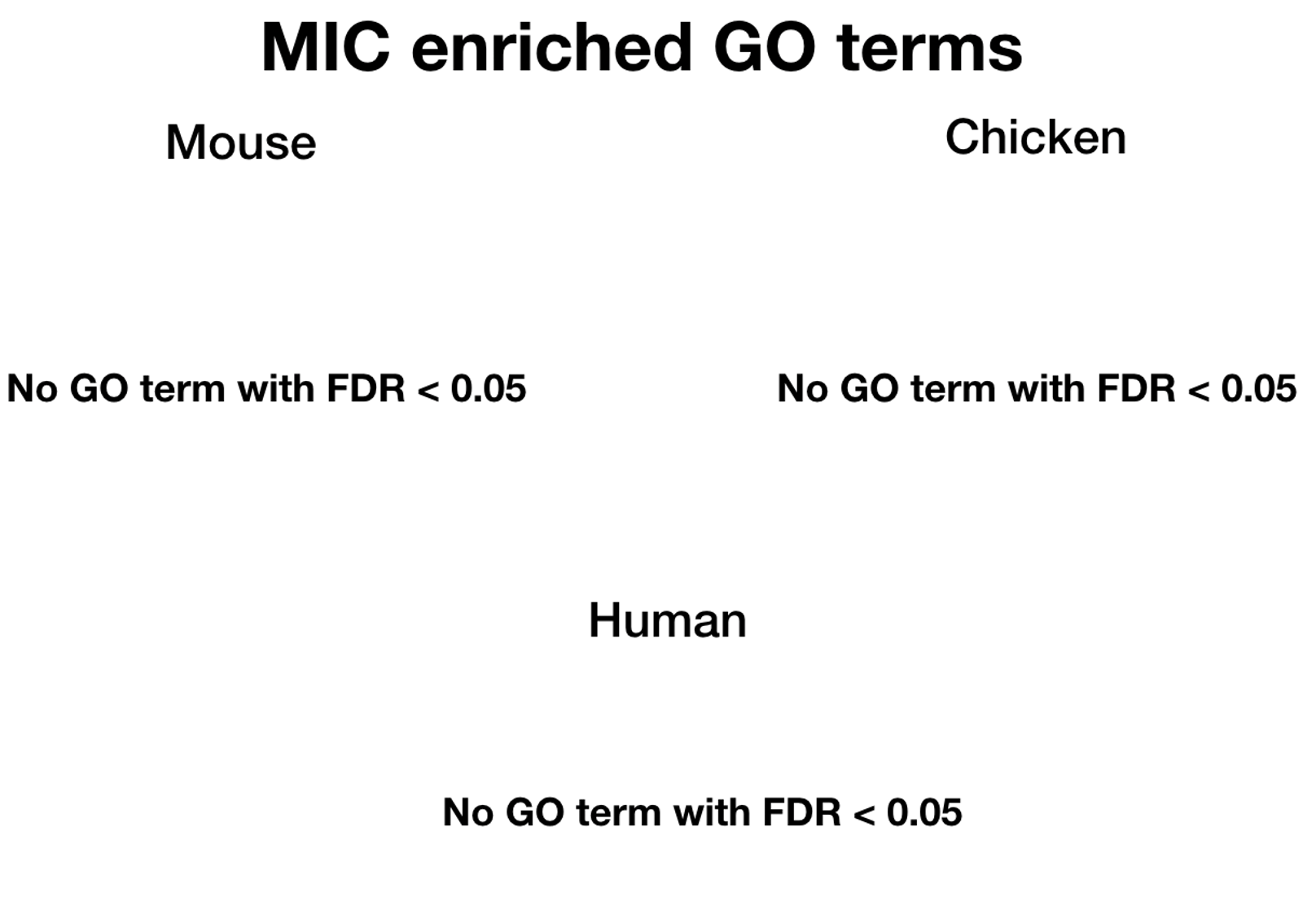

Supplement: S36 Fig — For our background dataset, we only used genes that have no paralogs. Removing the paralogs was to counter potential effects from MXE events being retained after a gene duplication, which could lead to overestimates of functional coherence of the gene set. (TIF) [file pcbi.1008708.s036.tif]

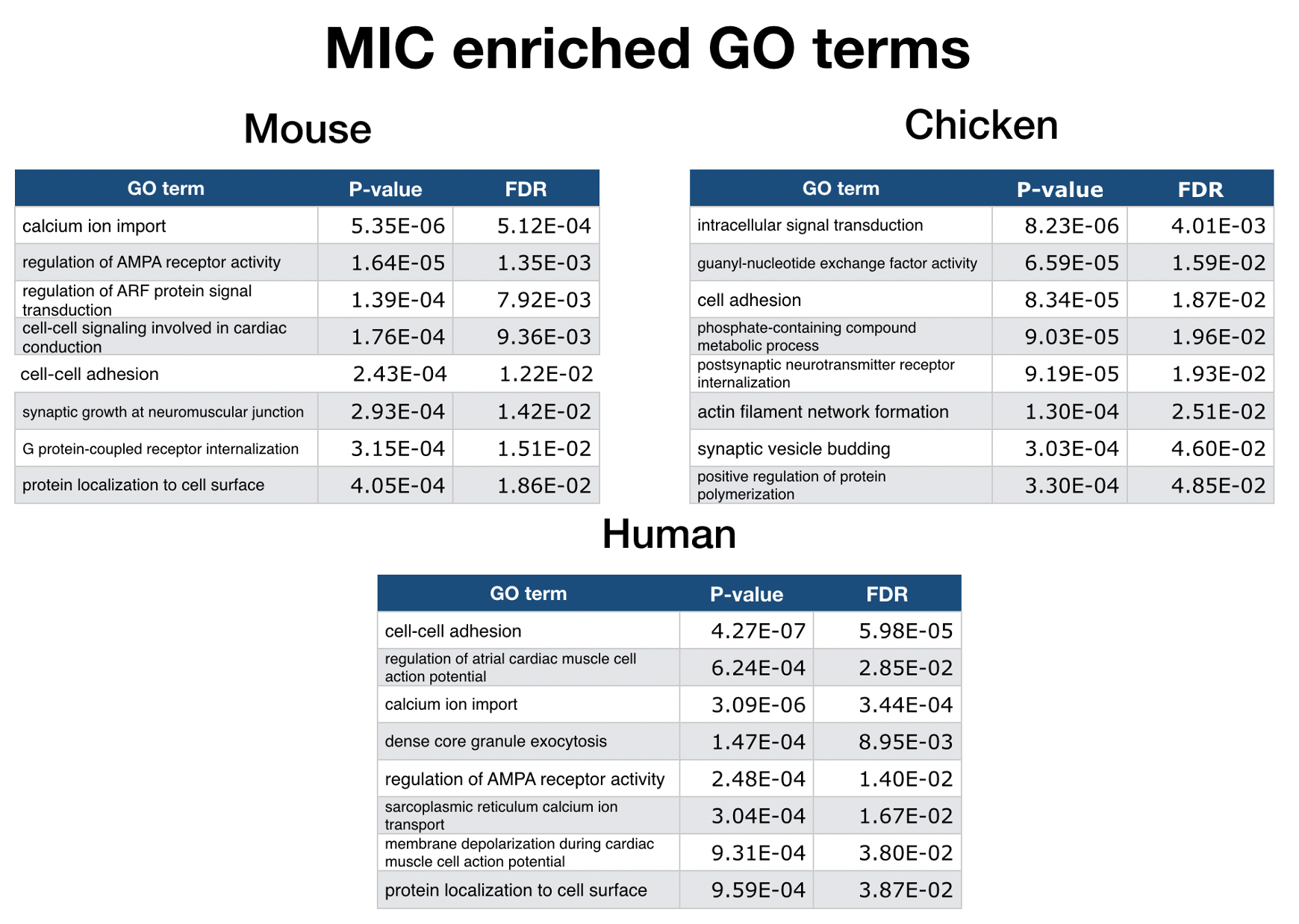

Supplement: S37 Fig — We used only multi-exon genes as background. Removing single exon genes was to counter potential effects of any functional bias, since by definition our MXE genes require more than one exon. (TIF) [file pcbi.1008708.s037.tif]

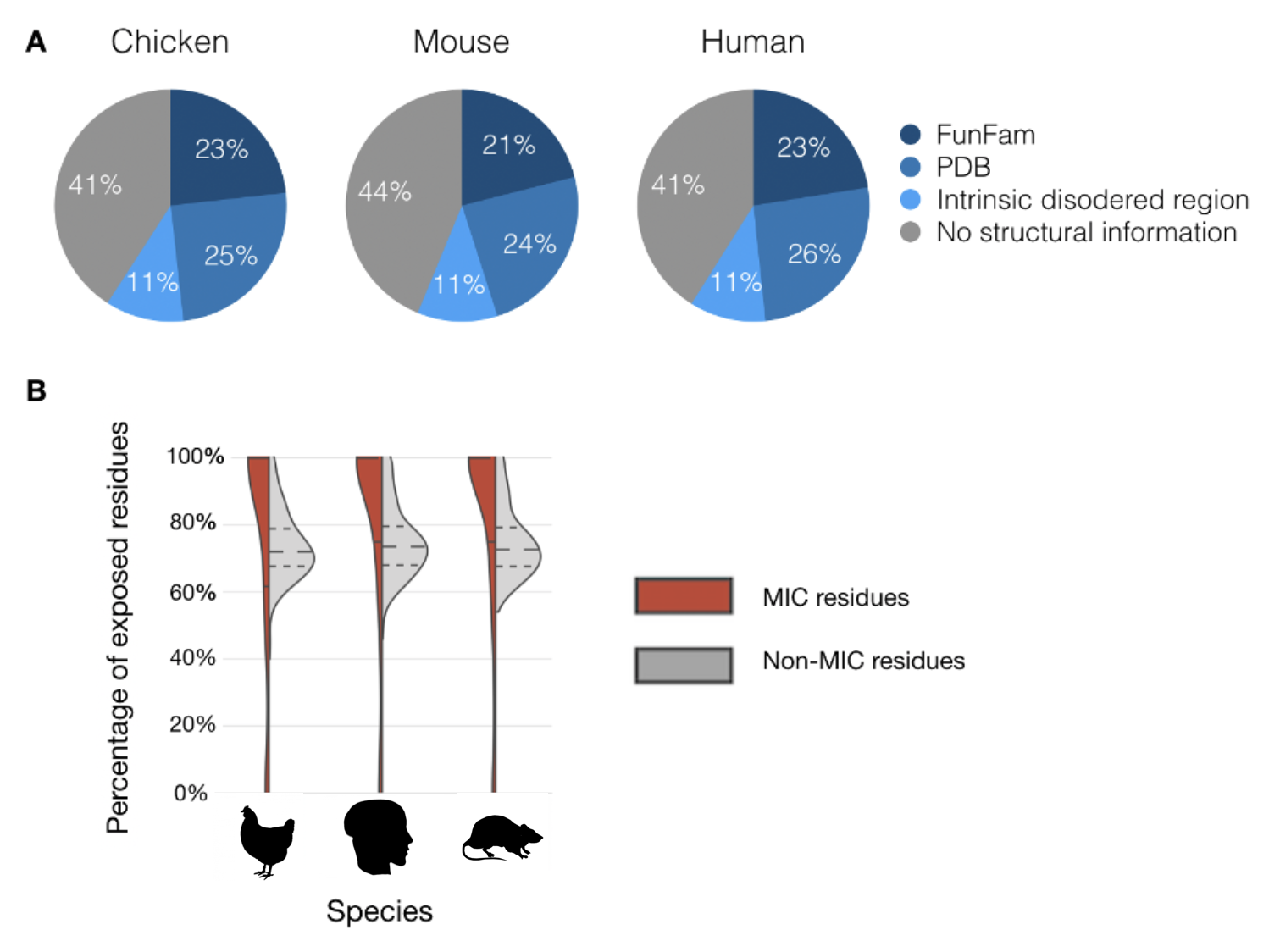

Supplement: S38 Fig — (A) Percentage of splice isoforms with structural information. (B) The surface exposure of the MIC residues compared to non-MIC residues. (TIF) [file pcbi.1008708.s038.tif]

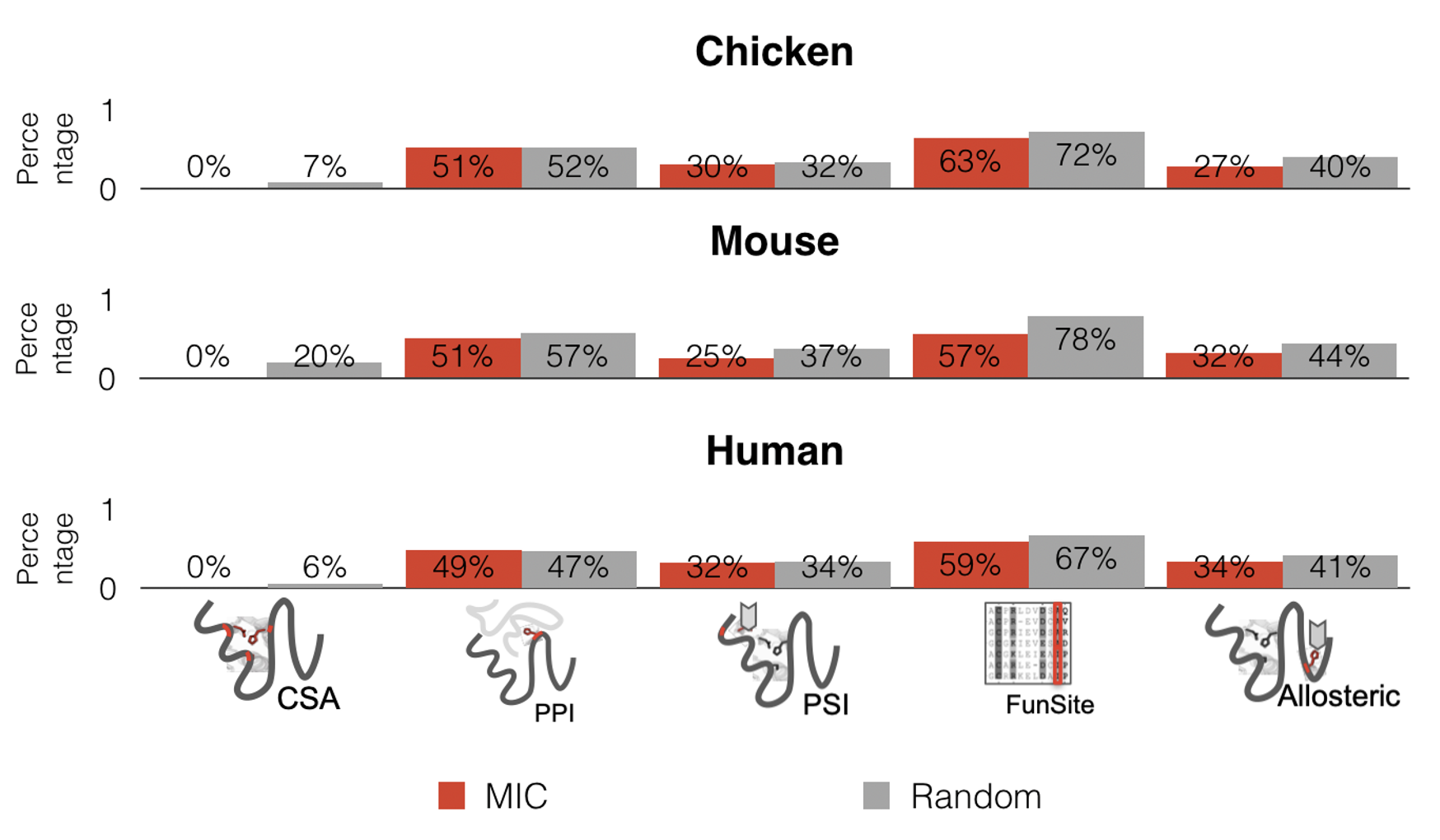

Supplement: S39 Fig — (TIF) [file pcbi.1008708.s039.tif]

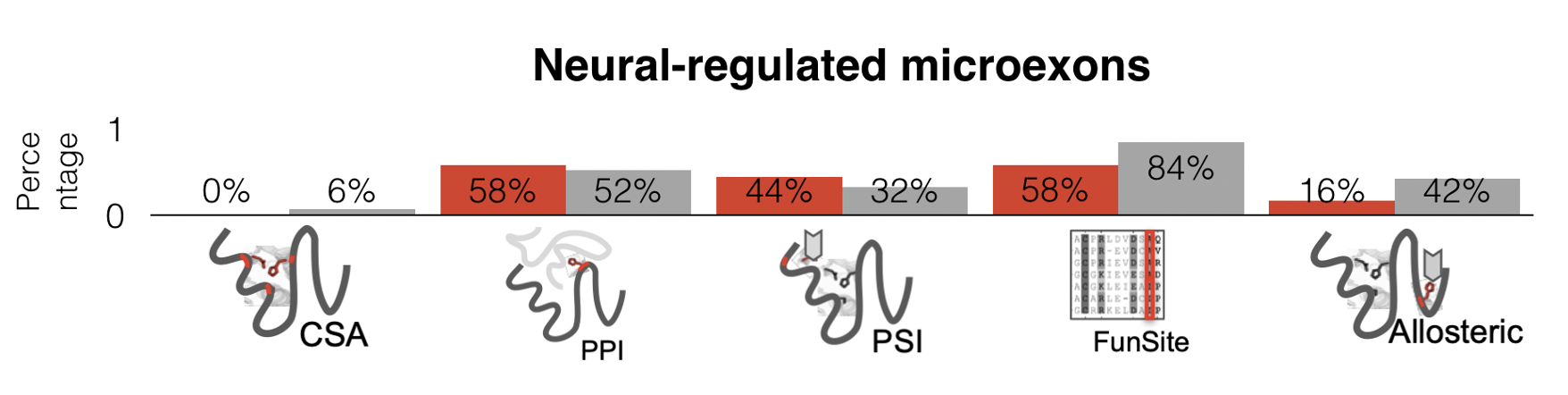

Supplement: S40 Fig — (TIF) [file pcbi.1008708.s040.tif]
