# Supplementary material for: Biological impact of mutually exclusive exon switching
Source: PLoS Comput Biol. 2021 Mar 2;17(3):e1008708. doi: 10.1371/journal.pcbi.1008708 (PMC7954323; doi:10.1371/journal.pcbi.1008708)
Supplement: S1 Text — (DOCX) [file pcbi.1008708.s041.docx]

**S1 Text. *The following section summarises the results analysing MXE events from the Hatje dataset.***

During the preparation of the manuscript, a large and extensive set of MXE-splicing annotations human became available (human validated-MXEs downloaded from the Kassiopeia website) [1]. We tested this additional dataset of RNAseq validated MXE-splicing events (that we refer to as the Hatje dataset). The Hatje dataset was generated using a MXE-splicing specific definition. Note for our analysis of the Hatje dataset we still filtered the events using the BLAST criterion **(see Materials and Methods section - Identifying MXEs and the amino acid sequence region affected by MXE events)** since we can only reliably perform the homology modelling and compare the isoforms by enforcing this.

The Hatje dataset contains a total of 455 Human MXEs (437 genes) with a median length of 25 amino acids (S19A Fig) and a sequence identity with a mean of 43% (S19B Fig). This value is lower than our dataset (sequence identity 67%). There are several differences in the definitions of the two datasets, one of which is that the Hatje dataset includes non-reference genome exons, which are supported by RNAseq data. Although providing more exons overall, one could speculate that including the non-reference genome exons may result in a higher rate of false positive MXE pairs, altering the sequence identity profile. Functional differences in protein sequences containing the MXE will arise from the variable residues. There are usually less than 13 variable residues (median = 11 residues) (S19C Fig).

The Hatje MXE regions structurally mapped to 75 of the CATH superfamilies. The MXE domain families are enriched in important Metazoan functions such as membrane proteins involved in membrane cell-cell adhesion and signal transduction. Functional analysis by the PANTHER pipeline showed additional functional enrichments, in membrane proteins (e.g. ion channels, synaptic vesicles and receptors) (FDR level <0.01; See S20-S24 Fig for details).

Our structural mapping approach provided structural information to assess 190 Hatje MXE splice events (S25A Fig). We observed that the percentage of exposed residues for a MXE region is significantly higher than what one would expect by chance (S25C Fig; p-value =6.21e^-46^, Wilcoxon signed-rank test). A more detailed analysis, involving buried surface area calculations for all the MXEs revealed that variable (i.e. MXE-specific) residues are much more significantly exposed than non-variable MXE residues (S25D Fig; p-value =9.1e^-3^, Wilcoxon signed-rank test). As with the analysis of our initial MXE dataset and associated results reported in Main, these findings collectively suggest a role for these surface variable regions in modulating protein functional sites.

For all the organism datasets, more than 90% of the MXE variable residues have a McLachlan score ≤2 (S25B Fig) indicating that, in general, the MXE events produce significant changes in physiochemical properties of the MXE regions. Such considerable changes suggest that these MXE events are likely to cause functional shifts between protein isoforms, particularly if they lie on or close to functional sites.

A comparison with a random model shows that there is a significant tendency for clusters of Hatje MXE variable residues to lie close to protein-protein interaction, protein-small molecule, specificity determining positions and allosteric sites for all species (S25E Fig). The signal was strongest for protein-small molecule interaction (PSI) and protein-protein interaction (PPI) functional sites (all with p-values <= 1.12e^-02^). We also performed the analysis without clustering the variable residues and obtained similar results.

We found microexons also had their highest enrichment in the brain with (the most enriched was for the middle temporal gyrus, FDR =5.63e^-51^, Fold enrichment = 1.67). The Hatje MXE genes had enrichments for the brain (with the top enrichment in the gyrus region, FDR =9.31e^-24^, Fold enrichment = 1.67). Analysis of our minimally disruptive cassette exon dataset showed the top enrichment to be “sensory system” (with a highly significant FDR = 3.16e^-74^ but low Fold enrichment = 1.21). Note that the point of the TopAnat analysis was to further characterise the high level functions of the MXE genes, rather than monitor which tissues their exons switch in.

Putative cancer driver genes from MutFams were mapped to 289 of our human MXE events. We could annotate 103 of these events with structures and we then determined if the variable residues/clusters in these MXE events were close to MutFam mutation residues. We found that 70 of the 103 MXE events had variable residues that were significantly closer (in terms of distance) to cancer mutations than expected by random (p-value <0.0001, see Materials and Methods). We obtained the same results for both per-residue and per-cluster analyses. We checked if these MXE events were close to functional residues and found 64 out of 103 of the events were close to functional residues. Hence, MutFam cancer mutations target a similar set of functional sites as are altered by MXE events, suggesting some advantage to the cancer in modifying the same set of specific functional residues on proteins that are dynamically regulated by MXE events.

References:

1. Hatje K, Rahman R, Vidal RO, Simm D, Hammesfahr B, Bansal V, et al. The landscape of human mutually exclusive splicing. Mol Syst Biol. 2017;13: 959. doi:10.15252/msb.20177728
