# Supplementary material for: Biological impact of mutually exclusive exon switching
Source: PLoS Comput Biol. 2021 Mar 2;17(3):e1008708. doi: 10.1371/journal.pcbi.1008708 (PMC7954323; doi:10.1371/journal.pcbi.1008708)
Supplement: S2 Text — (DOCX) [file pcbi.1008708.s042.docx]

**S2 Text. *The following section reports results obtained for the cassette exon analysis.***

To compare the structural and functional effects of MXEs with cassette exon (CE) splicing, we identified cassette single exon loss events (see **Materials and** **Methods**). Our dataset contains a total of 5459 CEs covering all the 5 organisms (S26A Fig) with a median length of 30 amino acids (S26B Fig). CEs cover about 5% of the whole sequence (S26C Fig).

MobiDB-lite was used to predict the residues that are disordered (CE regions with more than 50% of the residues predicted to be disordered are classified as disordered). We found that 15% of the cassette exons fall into disordered regions.

CE events (1323 events) can be mapped to 330 CATH superfamilies. This is a comparable proportion to MXE events (407 events mapped to 110 CATH superfamilies). Similar to MXE events, functional analysis by the PANTHER pipeline showed functional enrichments in membrane proteins (e.g. ion channels, synaptic vesicles and receptors) (FDR level <0.01, S27-29 Fig).

We also calculated the percentage of the domain which had been lost in the shorter isoform (using CATH-Gene3D domain assignments) (S30 Fig) and found that for 70% of the cases less than 30% of the domain had been deleted.

To compare the structural and functional effects of MXEs with cassette exon splicing we determined whether functional regions were proximal to the region of the structure that had been removed. We managed to model 18% of the CEs identified with acceptable models (nDOPE score below 0). For those that we managed to model, in 14% of the cases loss of an exon significantly affected the structure as evidenced by a reduction in the nDOPE structure quality score (i.e. giving a value above 0, see S31 Fig).

In addition to that, we compared the model quality of the models produced (between the isoforms) for MXE and CE events and found that difference in model quality (Δ nDOPE value) was larger for CE splicing than MXE splicing (S32 Fig*,* p-value = 7.0e^-19^, Mann-Whitney U Test). In other words, CE events are more likely to have a deleterious effect on the structure than MXE events.

For the cassette exon events where both isoform models had acceptable nDOPE scores (1003 events, 18%), we performed functional analysis and found no enrichment of MXEs close to functional sites (i.e. PPI, PSIs etc, S33 Fig).

It is worth pointing out that many cassette exon events are in disordered regions that we will not cover in our homology modelling approach and these would obviously be far less likely to damage the protein on removal.
