# Supplementary material for: Biological impact of mutually exclusive exon switching
Source: PLoS Comput Biol. 2021 Mar 2;17(3):e1008708. doi: 10.1371/journal.pcbi.1008708 (PMC7954323; doi:10.1371/journal.pcbi.1008708)
Supplement: S3 Text — (DOCX) [file pcbi.1008708.s043.docx]

**S3 Text. *The following section reports results obtained for the microexon analysis.***

To compare the structural and functional effects of MXEs with microexon splicing (MIC), we extracted the MIC events from VastDB (see **Materials and Methods**). Our dataset contains a total of 1230 MICs covering human, mouse and chicken (See S34A Fig). The MIC lengths spans from 1 to 9 amino acids, with a median length of 6 amino acids (S34B Fig). MobiDB-lite was used to predict the residues that are disordered and found 11% of the MICs fall into disordered regions.

270 MIC events can be mapped to 74 CATH superfamilies, a similar proportion to MXE and CE events. The MIC domain superfamilies are enriched in important Metazoan functions such as membrane proteins (actin-binding, channels, cytoskeletal maintenance, signalling (Pleckstrin homology-like (PH) domain)). Similar to MXE and CE events, functional analysis by the PANTHER pipeline showed functional enrichments in membrane proteins (e.g. ion channels, synaptic vesicles and receptors) (FDR level <0.01, S35-S37 Fig). In addition to that, MIC was found to be enriched with more specific terms such as SH3 domain and AMPA receptor (S35-S37 Fig). This result agrees with previous analyses that demonstrated that MIC events tend to be enriched with SH3 domains [1].

Our structural mapping approach provided structural information to assess 580 MIC splice events (S38A Fig). We observed that the percentage of exposed residues for MIC was significantly higher than for non-MIC residues (S38B Fig; p-value =1.3e^-09^, Wilcoxon signed-rank test).

For the MIC events where both isoform models had acceptable nDOPE scores, we performed functional analysis and found no enrichment of MICs close to functional sites (i.e. PPI, PSIs etc, S39 Fig).

Previous analyses had demonstrated that neural-regulated MICs are significantly enriched in domains that function in peptide and lipid-binding interactions [1]. We did another proximity analysis using only neural-regulated MICs and found no statistically significant enrichment close to PPIs and PSIs (S40 Fig).

References:

1. Irimia M, Weatheritt RJ, Ellis JD, Parikshak NN, Gonatopoulos-Pournatzis T, Babor M, et al. A Highly Conserved Program of Neuronal Microexons Is Misregulated in Autistic Brains. Cell. 2014;159: 1511–1523. doi:10.1016/j.cell.2014.11.035
