# Supplementary material for: Biological impact of mutually exclusive exon switching
Source: PLoS Comput Biol. 2021 Mar 2;17(3):e1008708. doi: 10.1371/journal.pcbi.1008708 (PMC7954323; doi:10.1371/journal.pcbi.1008708)
Supplement: S4 Text — (DOCX) [file pcbi.1008708.s044.docx]

**S4 Text. *The following sections give expanded details on the structural analysis of examples presented in the main text with some more details on methods.***

***Pyruvate Kinase*** The PKM gene codes for the dominant muscle isoform form of pyruvate kinase (PK) expressed in all human tissue except liver and red blood cells. There are two isoforms associated with an MXE splicing event, known as PKM1 and PKM2. Most PKM expressing tissues, (irrespective of whether they are healthy or cancerous, embryonic or adult), express the M2 isoform of PK, while skeletal muscle and some other cell types that require a very high glycolytic flux, express the constitutively active PKM1 isoform. Both the isoforms comprise 4 domains (See S13 Fig). The only difference between the isoforms is the MXE event located in the fourth domain (See S13 Fig). PKM1 is known to be a non-allosteric constitutively active tetramer [1]. By contrast, PKM2 has been detected as an inactive monomer, a less active dimer, and a tetramer (inactive T-state and active R-state), which are regulated by metabolic intermediates and post-translational modifications [2,3]. Activity of the R-state tetramer PKM2 depends on an allosteric effector molecule fructose 1,6-bisphosphate (FBP), a situation that has been associated to the feed-forward regulation of glycolysis.

The regulation of pyruvate kinase activity plays an essential role in metabolism and is particularly important in the growth of cancer cells [3]. PKM2 dimers and tetramers possess low and high levels of enzyme activity, respectively. Due to the key position of pyruvate kinase within glycolysis, the ability of PKM2 to switch between dimer and tetramer allows the tumour to modulate the flux of glycolysis. Low activity in PKM2 combined with higher glucose uptake pushes glucose carbons into anabolic pathways helping to support growth in proliferating cells.

There are known structures available for both PKM1 (PDB id 3SRF) and PKM2 (PDB id 1T5A) and domain 4 corresponds to CATH domains 3srfA01 and 1t5aA01. The overall sequence identity between the domains is high (87%) and the SSAP score between the PKM1 and PKM2 domains is very good (SSAP score 95). See Figure 5A for the structural superposition. It is known that PKM2 is activated by FBP but PKM1 is not.

Studies have shown that the FBP-binding residue 433 can be lysine, glutamate or threonine in different isoforms [4]. There is a change in position 433 between PKM1 and PKM2, with a lysine in PKM2 and a glutamate in PKM1. Mutation of either threonine (yeast pyruvate kinase - PK) or lysine (PKM2) to glutamate abolishes the FBP binding ability [5,6]. Mutation of glutamate in PKM1 to lysine enables the PKM1 mutant to bind FBP [7], presumably because FBP is negatively charged and thus a positively charged or neutral amino acid at this position would facilitate FBP binding whilst negatively charged amino acids would inhibit FBP binding and activation [4]. Therefore, the presence of lysine in PKM2 facilitates the binding of FBP.

To form the tetramer, domain 4 of the PKM monomers interact. The tetramer interface is located within the MXE region. We used the program Adaptive Poisson-Boltzmann Solver [8] to calculate surface potentials of MXE regions (See S14 Fig). 50% of the PKM1 MXE region has a strong negative net charge and 30% a strong positive net charge. We speculated this surface to be more complementary, facilitating tetramer formation. In contrast, PKM2 MXE region has a less complementary surface that is mainly neutral (40% of the surface neutral and 30% with a strong negative net charge).

Although surfaces in the PKM2 interface are less complementary, studies have demonstrated that allosteric binding of FBP in PKM2 induces movement/rotations of the helices in the interface and the FBP activating loop that brings the tetramer binding residues into correct alignment for forming the tetramer [2,9]. Therefore, the lack of interface complementarity in PKM2 without FBP bound and the increase when FBP is bound facilitates the switch of PKM2 between dimer (without FBP) and tetramer (with FBP bound).

There are 15 variable residues within the MXE that lie within the tetramer interface formed by domain 4. We used mCSM [10] to assess the effect of the MXE variable residue changes on protein-protein affinity. A large number of the variable residues (14 out of the 15) changing from PKM1 to PKM2 were found to be destabilising to the tetramer complex (See S15 Fig). Similarly, 13 out of the 15 variable residues from PKM2 to PKM1 were found to be destabilising to the tetramer complex (See S16 Fig). Our findings suggest that this may be due to co-evolution of packing (i.e. for both PKM1 and PKM2, the interfaces have co-evolved to be complementary).

Both the MXE regions of PKM1/PKM2 were found to overlap with pan-cancer mutations in structural regions close to functional sites, reinforcing the idea that mutations in these regions impact on the function of the proteins.

***Phosphofructokinase-platelet*** As demonstrated already in this paper, MXE events appear to be associated with functional switching events. However, MXE events are also rare, occurring in only a small percentage of genes. As highlighted above glycolysis is of major importance and has disease associations with cancer. PKM has already been shown above to have MXE splicing with possible relevance to regulation, where the MXE event provides a mechanism for allosteric activation of this enzyme. Surprisingly, when we looked at the other major allosterically regulated enzyme in glycolysis, phosphofructokinase (PFK) we also found a MXE event. Phosphofructokinase, PFK is a tightly regulated enzyme (e.g. sensitive to ATP/ADP levels in the cell) and is the first “committed” step in glycolysis (effectively an irreversible reaction, that commits the sugar into the pathway) by catalysing the phosphorylation of fructose-6-phosphate to fructose-1,6-bisphosphate (FBP).

We find several variable residues between the MXE isoforms of PFK-platelet (PFKP) including a change close to the Fructose-2-6-Bisphosphate allosteric effector binding site (V->I) at residue 539 (See Fig 5B). Although this is a conservative mutation, single amino acid changes from Valine to Isoleucine have previously been shown to change enzyme specificities [11]. When we computed the change of FBP ligand binding affinity on mutating V to I at residue 539 with mCSM-lig [12], we found that the PFKP-205 isoform with the isoleucine residue binds FBP more efficiently (See S17 Fig). We therefore suggest that this may affect the interaction of this isoform with FBP. Given the role of PFK in disease and its key role in glycolysis the PFKP MXE could provide an interesting experimental study.

***Collapsin Response Mediator Protein*** CRMP (FBgn0023023) is a dihydropyrimidinase enzyme (EC 3.5.2.2) that has been implicated in biological processes such as centrosome localization, pyrimidine nucleobase biosynthetic process, and positive regulation of Notch signalling pathway. There is a MXE event detected for the fly gene but currently no experimental structures for the fly isoforms. The MXE exons can be mapped to FunFam (3.20.20.140.FF40287) which is populated with dihydropyrimidinase sequences. We modelled both of the isoforms using the same template (CATH domain 1gkpC02, also a dihydropyriminidase enzyme). The models generated have a GA341 score of 1 and normalised DOPE score of -0.71 suggesting they are of good quality. The structures share a high structural similarity (SSAP Score above 91) and sequence similarity (79%) (See Fig 5C for a structural superposition with the splice regions highlighted). We used the program Adaptive Poisson-Boltzmann Solver to calculate surface potentials of the two isoforms.

Over 80% of the exposed splice region in CRMP-PA is positively charged. In contrast, the surface potential for CRMP-PB has a more negative charge that covers 60% of the exposed splice region (See S18 Fig). We found out that there is also a change in the zinc-binding residue 192, with a histidine in isoform CRMP-PA and a glycine in isoform CRMP-PB. This change is likely to disrupt the binding of the zinc molecule in the CRMP-PB isoform. Zinc molecules act as cofactors in dihydropyrimidinase enzymes. CRMP isoforms have been shown to have different tissue distributions and functions in fly [13].

References:

1. Israelsen WJ, Vander Heiden MG. Pyruvate kinase: Function, regulation and role in cancer. Semin Cell Dev Biol. 2015;43: 43–51. doi:10.1016/j.semcdb.2015.08.004

2. Wang P, Sun C, Zhu T, Xu Y. Structural insight into mechanisms for dynamic regulation of PKM2. Protein Cell. 2015;6: 275–287. doi:10.1007/s13238-015-0132-x

3. Wong N, Ojo D, Yan J, Tang D. PKM2 contributes to cancer metabolism. Cancer Letters. 2015. pp. 184–191. doi:10.1016/j.canlet.2014.01.031

4. Dombrauckas JD, Santarsiero BD, Mesecar AD. Structural Basis for Tumor Pyruvate Kinase M2 Allosteric Regulation and Catalysis. Biochemistry. 2005;44: 9417–9429.

5. Bond CJ, Jurica MS, Mesecar A, Stoddard BL. Determinants of allosteric activation of yeast pyruvate kinase and identification of novel effectors using computational screening. Biochemistry. 2000;39: 15333–15343. doi:10.1021/bi001443i

6. Lyssiotis CA, Anastasiou D, Locasale JW, Vander Heiden MG, Christofk HR, Cantley LC. Cellular control mechanisms that regulate pyruvate kinase M2 activity and promote cancer growth. Biomed Res. 2012;23: 213–217.

7. Ikeda Y, Taniguchi N, Noguchi T. Dominant negative role of the glutamic acid residue conserved in the pyruvate kinase M1 isozyme in the heterotropic allosteric effect involving Fructose-1,6-bisphosphate. J Biol Chem. 2000;275: 9150–9156. doi:10.1074/jbc.275.13.9150

8. Baker NA, Sept D, Joseph S, Holst MJ, McCammon JA. Electrostatics of nanosystems: application to microtubules and the ribosome. Proc Natl Acad Sci. 2001;98: 10037–10041. doi:10.1073/pnas.181342398

9. Morgan HP, O’Reilly FJ, Wear MA, O’Neill JR, Fothergill-Gilmore LA, Hupp T, et al. M2 pyruvate kinase provides a mechanism for nutrient sensing and regulation of cell proliferation. Proc Natl Acad Sci. 2013;110: 5881–5886. doi:10.1073/pnas.1217157110

10. Pires DE V, Ascher DB, Blundell TL. mCSM: predicting the effects of mutations in proteins using graph-based signatures. Bioinformatics. 2014;30: 335–342. doi:10.1093/bioinformatics/btt691

11. Yuan X, Yin P, Hao Q, Yan C, Wang J, Yan N. Single amino acid alteration between valine and isoleucine determines the distinct pyrabactin selectivity by PYL1 and PYL2. J Biol Chem. 2010;285: 28953–28958. doi:10.1074/jbc.M110.160192

12. Pires DEV, Blundell TL, Ascher DB. MCSM-lig: Quantifying the effects of mutations on protein-small molecule affinity in genetic disease and emergence of drug resistance. Sci Rep. 2016;6: 29575. doi:10.1038/srep29575

13. Morris DH, Dubnau J, Park JH, Rawls JM. Divergent functions through alternative splicing: The Drosophila CRMP gene in pyrimidine metabolism, brain, and behavior. Genetics. 2012;191: 1227–1238. doi:10.1534/genetics.112.141101
